# Umbelliferyloxymethyl phosphonate compounds-weakly binding zinc ionophores with neuroprotective properties†

**DOI:** 10.1039/d1dt02298a

**Published:** 2021-11-04

**Authors:** Sebastien Guesne, Laura Connole, Stephanie Kim, Majid Motevalli, Lesley Robson, Adina T. Michael-Titus, Alice Sullivan

**Affiliations:** Dept. of Chemistry, Queen Mary University of London Mile End Road London E1 4NS UK a.c.sullivan@qmul.ac.uk; Blizard Institute of Cell and Molecular Science, Barts and The London School of Medicine and Dentistry, Queen Mary University of London 4 Newark Street Mile End Road London E1 4NS UK

## Abstract

Umbelliferone is a member of the coumarin family of compounds which are known for diverse pharmacological activity including in targets relevant to Alzheimers disease, AD. The toxicity associated with some forms of the amyloid protein, Aβ, and the role of Zn^2+^ (and other biometals) dyshomeostasis in this, are of great interest in AD and make metal ionophore capability desirable in so called multi target drug ligands MTDLs. A new series of umbelliferyloxymethyl phosphonic acid diethylester compounds (umbelliferyloxymethyl phosphonates) bearing a phosphonate at the 7-position (compounds **1**, **3–6**), hydrolysis products **2**, **2a** and **2b** from **1** and analogues **7** and **8** of **1** with 7-O to 7-S and 1-O to 1-NH substitutions, are reported. Single crystal X-ray structures of compounds **1**, **2** and **2a** were determined. In terms of neuroprotective properties, the compounds **1**, **2**, **3**, **4**, **5** and **6** at 1 μM concentration, inhibited the toxicity of Aβ1-42 (Aβ42) in both toxic Amyloid Derived Diffusible Ligand (ADDL) and fibrillar (fibril) forms towards rat hippocampal cells. Compound **7** displayed cytotoxicity and **8** failed to inhibit Aβ42 toxicity. Concerning compound-metal ionophore activity (assessed using chemical experiments), despite weak binding to Zn^2+^ determined from ^31^P NMR titration of **1** and **2** by ZnCl_2_, compounds **1**, **3**, **4**, **5** and **6** demonstrated ionophore assisted partition of Zn^2+^ from water to octanol at micromolar concentrations with efficacy on a par with or better than the chelator MTDL clioquinol (5-chloro-7-iodo-8-hydroxyquinoline). Partition was assessed using furnace Atomic Absorption Spectroscopy (AAS). In further experiments interaction of compound **1** with Zn^2+^ or it's pathways was inferred by (i) delayed fluorescence response with added Zn^2+^ in cells treated with FluoZin-3 and (ii) by suppression of Zn^2+^ promoted aggregation of Aβ42.

## Abstract 2

Umbelliferyloxymethyl phosphonates:non-chelating ionophores that **A** protect rat hippocampal cells from toxic Aβ42 **B** partition Zn from water to octanol **C** inhibit Zn promoted aggregation of Aβ42, **D** delay Zn signaling by FluoZin-3 in neuronal cells.

## Graphical abstract

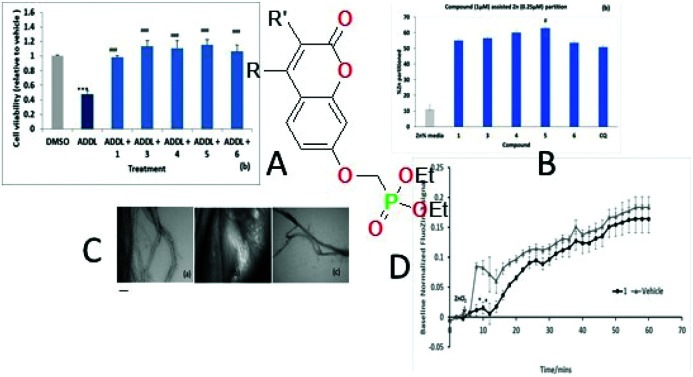

## Introduction

**Fig. 1:**
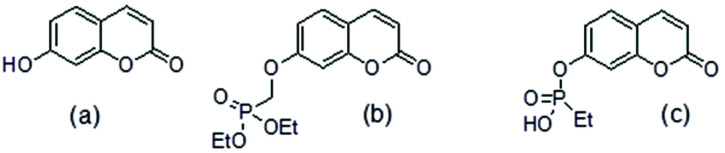
(a) umbelliferone (b) umbelliferyloxymethyl phosphonic acid diethylester (c) ethylphosphonic acid umbelliferyl ester.

Molecular ligands that can affect more than one AD target pathway, so called multi target directed ligands, MTDLs,^1^ are of interest as potential therapeutic agents. Several umbelliferone (Fig. 1(a)) compounds have been reported to be active towards more than one AD target such as Aβ42 aggregation, Aβ42 cytotoxicity to P12 neuroblastoma cells, inhibition of enzymes such as human mono amine oxidases, MAOs, cholinesterase and β-secretase. As such they have characteristics of MTDLs. Examples of these compounds are in Table 1.

**Table 1:** Umbelliferone compounds with MTDL properties

Compounds	AD targets	Ref.
6-Formyl-umbelliferone	Inhibitors for *h*MAO-A, lipid peroxidation, self-aggregation of Aβ(25–35), cholinesterase and BACE1	2 and 3
4-Methyl-umbelliferyloxymethyl-1(3-methylbenzyl)pyridin-1-ium bromide	Inhibitors for *h*MAO-B, self-aggregation of Aβ42, cholinesterase	4
6-Methoxy-umbelliferone (scopoletin)	Inhibitor for self-aggregation of Aβ42, cholinesterases and 69% protection against Aβ42 in PC12 cells at 40 μM	5

Aβ42 peptide assemblies such as ADDLs (amyloid derived diffusable ligands) and soluble fibril forms are examples of toxic forms of β-amyloid that can be used for assessing the activity of MTDLs *in vitro*.^6^ Small oligomeric assemblies of β-amyloid cause synaptic loss, reduction in dentritic spines, suppression of LTP (long term potentiation) and blocking of various neuronal signaling pathways.^7^ Furthermore biometals such as Zn^2+^ affect the different aggregation states and toxicity of Aβ peptides and dyshomeostasis of these metals is strongly linked to AD pathology.^8–11,12^ Molecular chelators have been shown to demonstrate metal sequestration, translocation and associated neuroprotective benefits from this while also inhibiting other AD hallmarks.^10^ Possible modes of action include (i) inhibition of specific toxic oligomers of Aβ or the formation of these and (ii) roles as receptor antagonists and/or ionophore interactions with Zn^2+^ that promote neuroprotective enzyme activity. The hydroxyquinoline chelator compounds 5-chloro-7-iodo-8-hydroxyquinoline (clioquinol or CQ) and 2-methyl(dimethyl)amino-5,7-dichloro-8-hydroxyquinoline (PBT2) are MTDLs that displayed very promising performance against hallmark AD targets in mouse models and were tested in small human cohort phase II clinical trials. Outcomes were only partially positive against the trial AD test targets but despite this the strategy of small molecule metal ionophores with effects on hallmark AD targets remains a very compelling approach.^8,12–14^ In addition to the hydroxyquinolines and aminoquinolines,^13^ many other chelating motifs displaying MTDL properties have been reported,^10^ including for example derivatives of pyrazoles,^15^ phenol-triazole,^16^ hydrazones,^17^ and thioflavin T.^18^

In this paper we report on work that aimed to extend the variety and scope of multi target drug ligand (MTDL) umbelliferyl compounds, targeting Alzheimer's disease hallmarks. Thus, the new umbelliferyloxymethyl phosphonate compounds and analogues **1–8** were synthesised and evaluated in the first instance as inhibitors of cytotoxic Aβ42 in both ADDL and fibril forms. In terms of small molecule MTDLs as potential metal ionophores in AD, chelator ligands are extensively studied^10,12^ but monodentate weakly binding ligands may also have ionophore activity and potentially also assist free metal translocation without systemic depletion. The monodentate compound 1,3,5-triaza-7-phosphaadamantane (PTA) was shown to remove copper from Aβ16 and prevent ROS production and also to inhibit aggregation of Aβ40.^19^

We were motivated to assess by physico-chemical means the ligand ionophore chemistry of non-chelating umbelliferyloxymethyl phosphonates with respect to Zn^2+^. The effect of **1** on Zn^2+^ promoted aggregation of Aβ42 and on Zn^2+^ signaling by cell internalized FluoZin-3 is also reported. The results of our synthetic, biological and chemical experiments are reported here.

## Results and discussion

### Compound synthesis and characterisation

**Fig. 2:**
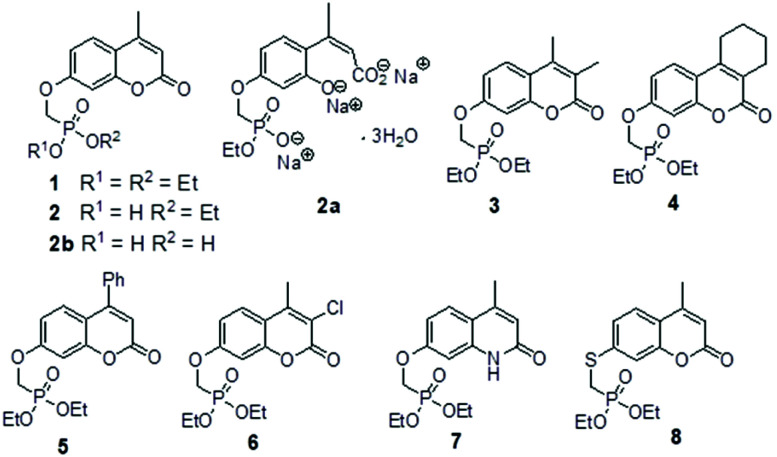
Chemical structure of compounds **1**, **2**, **2a**, **2b**, **3**, **4**, **5**, **6**, **7** and **8**.

The umbelliferyloxymethyl phosphonic acid diethylester compounds described in this paper have hydrolytically and metabolically stable phosphonate connectivity umbelliferyl-O-CH_2_-P(O)(OEt)_2_ as in Fig. 1 structure (b). Umbelliferyl phosphonate derivatives such as ethylphosphonic acid umbelliferyl ester Fig. 1 structure (c) with more easily hydrolysed umbelliferyl-phosphonate connectivity umbelliferyl-O–P(O)(OR)_2_ have been reported.^20^ Phosphonate diesters RPO(OR′)_2_ are commonly studied in the context of prodrugs.^21–23^ Phosphonate ester susceptibility to phosphatase hydrolysis varies and simple alkyl esters, methyl or ethyl, are more resistant.^21,22^ Synthetic schemes for the new umbelliferyl phosphonate derivatives and analogues (structures in Fig. 2) are given in the ESI (S3–S5†).

In each case the diethyl phosphonate moiety was introduced at the 7-position of the parent umbelliferone or its analogues by nucleophilic substitution employing diethyl tosyloxymethylphosphonate, DETOMP. The mono acid derivative **2** was obtained from parent **1** by alkaline (pH > 10) phosphonate ester hydrolysis at 50 °C followed by acidification (pH ∼ 1). An intermediate compound **2a** from the alkaline hydrolysis of the lactone moiety and phosphonate ester in **1** (carried out at 70 °C without the acidification step) was also isolated and characterised by X-ray crystallography. The phosphonic acid **2b** was formed by Me_3_SiI mediated hydrolysis of **1**. The compounds were characterised by NMR (^1^H, ^13^C, ^31^P) and IR spectroscopy and high resolution mass spectrometry. Details are given in the Experimental section and (S6–S39†). A combination of one and two-dimensional experiments involving long-range (multiple bond) ^1^H–^13^C correlation spectroscopy were used to confirm the NMR assignments (example given S34 and S35†). The composition of the new compounds was confirmed by the appearance of molecular ions in their high-resolution mass spectra.

**Fig. 3:**
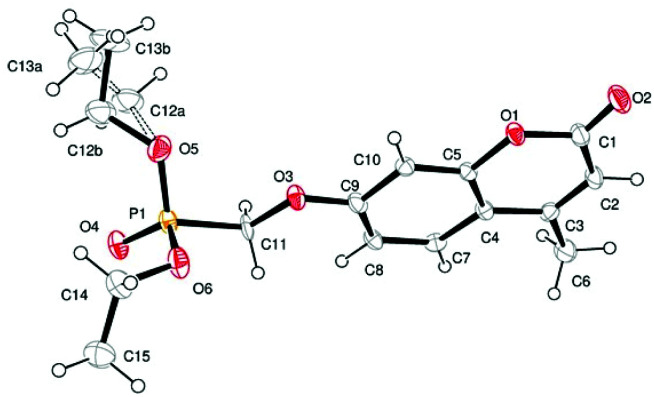
ORTEP image of compound **1** showing modelled disorder in one ethyl ester group.

**Fig. 4:**
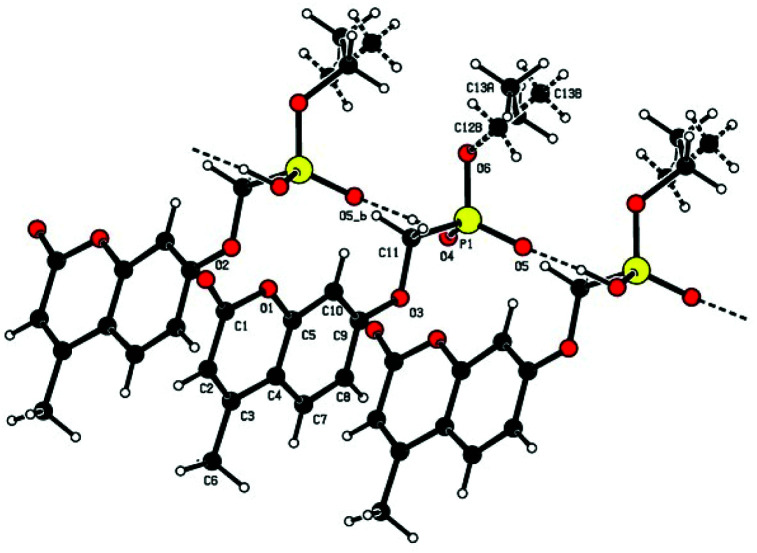
Chain structure in **2** with intermolecular P=O⋯HO–P hydrogen bonding and showing modelled disorder of the ethyl ester group.

**Fig. 5:**
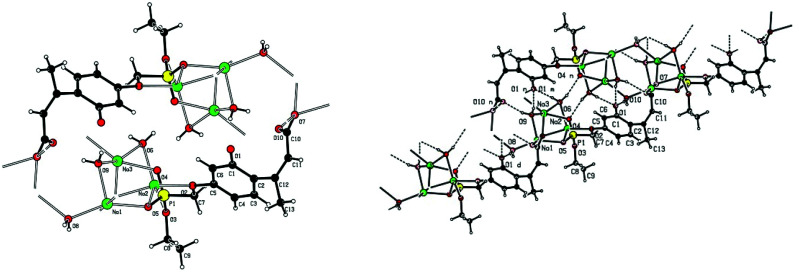
X-ray structure of **2a** trisodium 3-[4-(ethoxy-phosphonatomethoxy)-2-phenolato]-but-2-enoate trihydrate. Left: showing Na^+^–O–P, Na^+^–O–C and Na^+−^OH_2_ bonding. Right: the extended hydrogen bonded chain structure.

Single crystal X-ray structures of **1**, **2** and **2a** are shown in Fig. 3, 4 and 5.

Crystallographic data including selected bond lengths and angles are given in (S40–S43†). Diester phosphonate compound **1**Fig. 3, crystallised as discrete molecules in the solid state with no close intermolecular contacts. Monoacid compound **2** displayed a hydrogen-bonded chain structure wherein each phosphonate was hydrogen bonded to two others *via* the phosphonyl oxygen P=O and the acid P–OH moieties as shown in Fig. 4.

The trianionic units of **2a** (Fig. 5) are arranged in linear chains top to tail with Na contacts linking the carboxylate end of one molecule to the phosphonate end of the next. Connections between adjacent chains are facilitated by a sandwiched layer of hydrated sodium ions that connect anionic phosphonate or carboxylate oxygens on one chain to acceptor oxygen sites on the adjacent chain such as, P–O^−^⋯^+^Na—OH_2_⋯O–P or Aryl-O⋯H_2_O—Na^+^⋯^−^O—P. Adjacent chains are related by a two-fold symmetry axis and a mirror plane. The non-polar phosphonate ethyl ester moiety and the methyl substituent of the but-2-enoate fragments are directed away from the polar hydrated sodium centre of the double chain structure.

### Evaluation of compound neuroprotective effects towards toxic Aβ42 ADDLs and fibrils

**Fig. 6:**
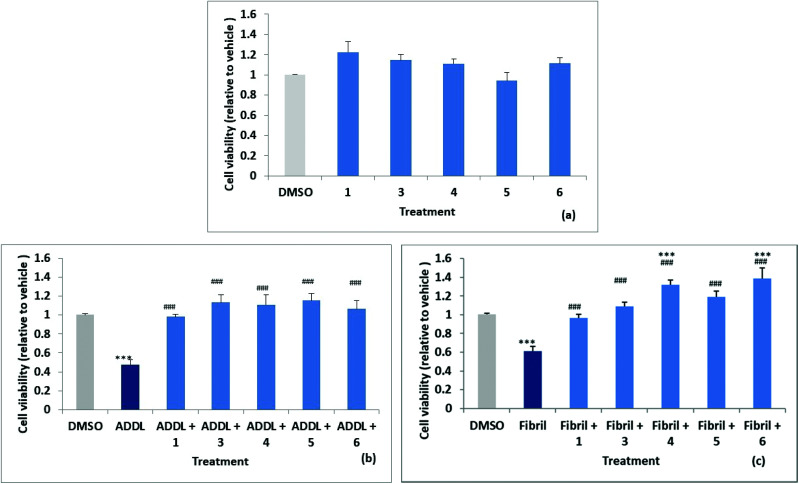
MTT assays on hippocampal cell viability (a) effect of compounds after 72 h incubation. Data (*n* = 3) are expressed as mean ± SEM and analysed using a one-way ANOVA. Effect of compounds on ADDL (b) and fibril (c)-induced cytotoxicity after 72 h incubation. Data (*n* = 3) are expressed as mean ± SEM and analysed using a one-way ANOVA with a Newman–Keuls multiple comparison *post-hoc* test (***P < 0.05 *vs.* vehicle; ###*P* < 0.05 *vs.* ADDL or Fibril.

Stock solutions of amyloid-derived diffusible ligand (ADDL) and fibril forms of Aβ42 were prepared as described in the experimental (and S52†) following reported protocols.^6^ A reverse peptide, Aβ42-1, was prepared in an identical manner and used as a control for non-specific effects. The effects of the reverse peptide on cell viability were not significant compared to the vehicle control (0.1% DMSO) (results not shown). Rat primary hippocampal neurons were treated with vehicle control (0.1% DMSO) and the two forms of Aβ42, ADDL and fibril, in the absence and in the presence of the compounds, for 72 h. Treatments were performed in triplicate wells and repeated in three separate biologically independent cultures. MTT (3-(4,5-dimethylthiazol-2-yl)-2,5-diphenyltetrazolium bromide) assays, which measure the reduction of the yellow MTT dye to purple formazan by mitochondrial enzymes in healthy cells, was used to assess cytotoxicity. The results are summarised in Fig. 6a, where cell viability values are expressed relative to the vehicle control. The cell survival data for compounds **1**, **3**, **4**, **5** and **6**, showed no significant difference relative to the vehicle control. Significant neuronal cell death was observed relative to the vehicle when cultures were exposed to ADDLs or fibrils in the absence of compound with viability reduced to 53 ± 5% and 62 ± 5% respectively, see Fig. 6b and c. Cell viability was restored however, when cells were exposed to ADDLs or fibrils in the presence of compounds **1**, **3**, **4**, **5** or **6**, demonstrating the protective effect of these compounds. For compounds **4** and **6** there was also some indication of cell proliferation in the tests with fibrils Fig. 6c.

We can also report (data not shown) that cell viability was not affected by mono acid **2** but viability restoration by **2** in the presence of ADDL or fibril at 83 ± 5% and 86 ± 6% respectively was less than observed for the parent **1** which completely inhibited the cytotoxic Aβ42. Predicted log *D* values (from Optibrium Stardrop software) for compounds **1** 2.323, **2** 0.461 and **2b** 0.14 were consistent with decreasing lipophilicity from the diester to the diacid. This may cause poorer cell membrane penetration for **2** and contribute to the lower observed protective effect. Compounds **1** and **3–6** with different substituents at the 3,4-position have predicted log *D*s in the range 2.303–3.355.

Compound **7** (with 1-NH substitution) exhibited significant reduction in cell viability and while **8** (with 7-S substitution) did not statistically affect cell viability, it failed to inhibit ADDL or fibril toxicity.

### Evaluation of compound affinity and ionophore efficacy for Zn

As stated in the introduction management of Zn^2+^ homeostasis compromised by Aβ oligomers in AD is postulated as a possible role for small molecule ionophores.^8–13^ Despite likely low ligand denticity, this motivated us to investigate affinity and ionophore activity of our phosphonate compounds towards Zn^2+^. Phosphonate diester metal compounds with metal to phosphonyl oxygen (P=O⋯M) coordination have been reported both by us and others.^24,25^ Compounds **1**, **3**, **4**, **5** and **6** differ in terms of non-coordinating substituents at the 3,4-ring positions and **1** was selected from this group to evaluate possible P=O⋯M complexation with Zn^2+^. Compounds **7** and **8** having failed to display neuroprotective activity were omitted from further study. However, attempts to isolate metal complexes from solutions of Zn^2+^ salts treated with **1** in several stoichiometric ratios afforded only starting materials. The divalent metal ion Zn^2+^ had very little effect on the intrinsic fluorescence of compound **1** in fluorescence titration experiments (see S44a†). Similarly, the electronic absorption bands of **1** were not significantly perturbed by added ZnCl_2_ in UV-Vis titration experiments. No ligand to zinc charge transfer bands were detected so this method did not prove useful to study zinc binding (see S44b†). ^31^P NMR titration experiments of **1** (14 mM) with ZnCl_2_ (using 0.05 molar equivalent titre aliquots) in CDCl_3_ displayed small incremental down-field shifts peaking at 0.25 equivalents of added ZnCl_2_ (maximum shift 0.5 ppm S45†). This was indicative of a species involving ZnL_4_ dominating in solution. If we consider the possibility of an L_4_ assembly in solution acting as a discrete host L′ for ZnCl_2_ then analysis of the ^31^P NMR titration data using Benesi–Hildebrand method^26^ for ZnL′ formation gives *K*_a_ 866 ± 52 M^−1^ (S45†). The ^1^H NMR of **1** : 0.25ZnCl_2_ solution showed the oxymethyl proton resonance (umbelliferyl-OC**H**_**2**_P(O)(OEt)_2_) shifted downfield while all other resonances remained unchanged (S45†). This is in-keeping with a Zn to phosphonyl oxygen interaction Zn—O=P(CH_2_O—umbelliferyl)(OEt)_2_. In contrast to **1**, ^31^P NMR titration of compound **2** (14 mM) in CDCl_3_ displayed up field shifts up to 1 equivalent of added ZnCl_2_ (maximum shift 3.4 ppm). This is indicative of deprotonation of the mono acid and possible Zn^2+^ binding at this site. Analysis of the titration data using the Benesi–Hildebrand method^26^ for a 1 : 1 complex gave *K*_a_ 546 ± 33 M^−1^ for **2** (S46†) indicative of weak binding to Zn^2+^.

**Fig. 7:**
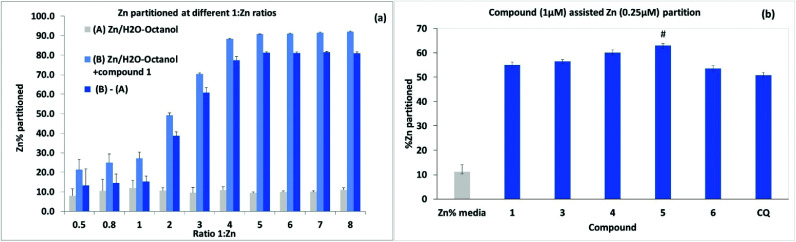
Percentage aqueous metal partitioned after 24 h in 1 : 1 aqueous–octanol (a) with 4 μM **1** for **1** : Zn^2+^ ratios 0.5–8. (b) with 1 μM compound and 0.25 μM Zn^2+^. The mean value for the media control (Zn^2+^ in 1 : 1 aqueous–octanol) is shown in (b) and was subtracted from compound data. Data (*n* = 3) are expressed as mean ± SD analysed using one way ANOVA and *post hoc* comparison: # *P* < 0.05 *vs.***4**.

Further experiments to assess metal ionophore activity for the umbelliferyloxymethyl phosphonates were carried out using furnace Atomic Absorption Spectroscopy (AAS) to measure compound assisted partition of Zn^2+^ from water to octanol in biphasic mixtures. Details of calibration and experimental protocols are given in the Experimental section (and S47–S51†). Trace amounts of zinc in the ultrapure water used were factored into solution preparation. The initial experiments were run using a fixed concentration of 4 μM **1** and varying Zn^2+^ to give ratios of **1** : Zn^2+^ between 0.5 and 8.0. Data presented in Fig. 7(a) are based on the means from measurements taken on five samples from each different Zn^2+^ concentration replicated in three separate experiments. Results showed compound assisted Zn^2+^ partition peaking at close to 80% for 4 μM **1** and 1 μM Zn^2+^. The ^31^P NMR titration also peaked close to **1** : Zn^2+^ ratio of 4. When the partition experiment was repeated for 4 μM **1** and 1 μM Cu^2+^ the equivalent compound assisted partition was 42.7 ± 4% indicating significantly higher efficacy for Zn^2+^. Zinc partition was subsequently assessed for the set of compounds **1**, **3**, **4**, **5** and **6** (and Clioquinol, CQ, as a known chelating ionophore) employing 1 μM compound and 0.25 μM metal (Fig. 7). Under these more dilute conditions Zn^2+^ partition by compound **1** was lower as expected (by ∼22%) than for 4 μM **1** : 1 μM Zn^2+^. Overall, the compounds **1**, **3**, **4**, **5**, **6** and CQ markedly increased Zn^2+^ partition relative to the media with slightly higher efficacy for compound **5** which also had the highest predicted value for log *D* 3.355. It is notable that the ionophore activity of the umbelliferyloxymethyl phosphonate compounds was on a par or slightly better than that of the chelator MTDL CQ in these experiments.

### Evidence for compound **1** interacting with Zn or Zn pathways in experiments involving hippocampal cells and Aβ42 fibrils

Further work is required to establish whether the compounds can assist metal transport in biological systems. We have observed however, in two biological experiments, that compound **1** can modify Zn^2+^ activity.

**Fig. 8:**
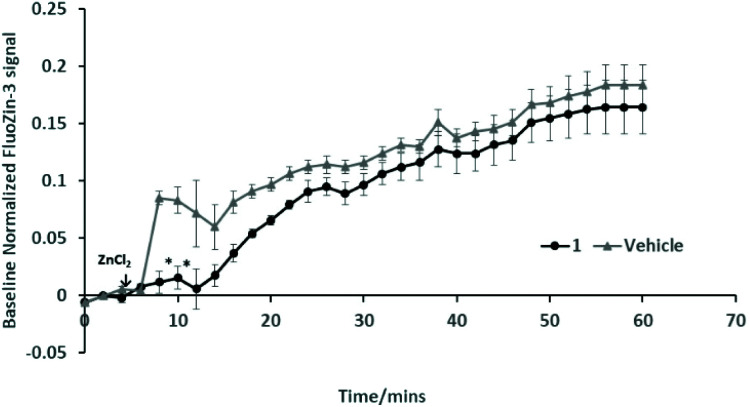
Effect of compound **1** on stimulated Zn^2+^ signaling by FluoZin-3. Fluorescence (Ex/Em 494/516) was measured at 2 min intervals for 60 min with addition of ZnCl_2_ (30 μM) at 6 min. Average baseline values were recorded for the first 6 min for each treatment and used to normalize fluorescence at all time points. The data (*n* = 3) are analysed using a two-way ANOVA with Bonferroni *post-hoc* test (*P < 0.05 *vs.* vehicle).

**Fig. 9:**
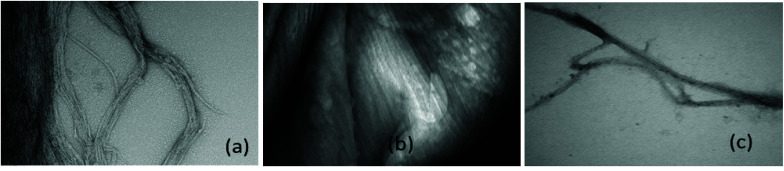
SEMs after treatment Aβ42 (5 μM) as follows (a) constant agitation 72 hours pH 7.4 @ 37 °C (b) as in (a) including 5 μM Zn^2+^ (c) as in (a) including Zn^2+^ (5 μM) and compound 1 μM **1**. Bar = 100 nm. Magnification 49 000×.

In the first case, the aim was to observe the effect of compound **1** on Zn signaling in FluoZin-3 treated hippocampal cells. The cells were incubated with the high affinity Zn^2+^ specific probe FluoZin-3, washed twice and then further incubated in fresh media with either compound **1** (1 μM) or vehicle DMSO. The probe fluorescence was monitored for 1 h for both compound **1** and vehicle treated cells before and after treatment with ZnCl_2_ at the 6 min time point (Fig. 8). The zinc probe fluorescence response was lower for 16 min after addition of ZnCl_2_ to cells incubated with compound **1** compared to the vehicle control. The delayed response may be interpreted as interaction between compound **1** and the ZnCl_2_ or alternatively the metal's transmembrane pathway to the internalized probe. In the second example the aim was to observe the effect of compound **1** on well documented Zn^2+^ promoted aggregation of Aβ42.^27,28^ Thus, solutions of Aβ42 (5 μM) (a) neat, (b) with added Zn^2+^ (5 μM) and (c) with Zn^2+^ (5 μM) and compound **1** (1 μM) were agitated for 72 h and subsequently examined by SEM Fig. 9. The extensive aggregation observed for treatment (b) was not observed for treatments (a) or (c) confirming that the Zn^2+^ promoted aggregation of Aβ42 where the latter is a hallmark in AD was impeded by compound **1**. The observation may indicate an interaction between compound **1** and Zn^2+^ or **1** and aggregation nucleation sites on the amyloid.

## Conclusions

Umbelliferone compounds are widely reported to be biologically active towards a range of targets including some that are relevant in AD. A new family of umbelliferyloxymethyl phosphonic acid diethyl ester compounds, hydrolysis derivatives and analogues have been synthesised and characterised. With the exception of analogue compounds **7** and **8**, containing key heteroatom substitutions in the umbelliferone moiety, 7-S for 7-O and 1-NH for 1-O respectively, the compound set was found to be protective in rat hippocampal cells against toxic fibrillar and oligomeric (ADDL) Aβ42. With respect to Zn^2+^ interactions, the ^31^P NMR study suggested weak Zn^2+^ binding affinity while the AAS study confirmed Zn^2+^ ionophore activity for our compounds on a par with the chelator ionophore CQ. Compound **1** caused delay in Zn^2+^ stimulated signaling response from FluoZin-3 probe incubated in rat hippocampal cells, pointing to interaction of **1** with Zn^2+^ or the metal transmembrane pathways. ESEM images provided qualitative evidence that compound **1** impeded Zn^2+^ promoted aggregation of Aβ42. Collectively the synthesis, biological and chemical data we report in this paper extends the variety and chemistry of MTDL umbelliferone derivatives and data related to these that is of interest to AD. The study can be a basis for further evaluation of the compounds for Zn^2+^ transport in biological systems and as inhibitors of other AD hallmarks.

## Experimental

### Materials

Solvents, dimethylformamide DMF, tetrahydrofuran THF, dichloromethane DCM, diethyl ether and toluene were purified on a MBraun SPS-800 Solvent Purification System. Dimethyl sulfoxide DMSO and DCM were dried over and distilled from molecular sieves and calcium hydride respectively and stored over molecular sieves. Chemical and biological reagents, standards and solvents were purchased and used as supplied from the following suppliers: Sigma Aldrich, Alfa Aeser, Lancaster, Appolo Scientific, Avocado, PCAS seqens, California Peptide Research, Inc. (California, USA), Cambridge Bioscience Ltd, Generon Ltd, Cambridge Isotope Laboratories, Invitrogen, VWR and Fisher Scientific.

Instrumentation used: (a) Varian 220FS SpectrAA Atomic Absorption Spectrometer with GTA 110. (b) Bruker 270 or 400 MHz NMR spectrometers; reference chemical shifts (*δ* ppm) employed residual protonated solvent in, CDCl_3_ (^1^H, 7.26 ppm, ^13^C, 77.16 ppm) and DMSO-*d*_6_ (^1^H, 2.50 ppm, ^13^C, 39.52 ppm). (c) SHIMADZU FTIR 8300 equipped with an ATR (Attenuated Total Reflectance) attachment in the range of 4000–600 cm^−1^. (e) Yvon Jobin Fluoromax 3. (f) NSI-High Resolution Mass Spectra (NSI-HRMS) were recorded on an LTQ Orbitrap XL at the EPSRC Mass Spectrometry service. (g) FEI Quanta ESEM.

Full NMR, IR and Mass Spectra are available in ESI S6–S39.† Crystallography: Details of instrumentation, crystallographic refinement data and selected bond lengths and angles are given in ESI S40–S43.†

Chromatographic purifications were performed on Silica gel 40–63 μm for flash chromatography. Melting points were observed on an Electrothermal melting point apparatus. Log *D* values were predicted using Optibrium Stardrop software.

All animal procedures were approved by the Animal Welfare and Ethical Review Body at Queen Mary University of London, and the UK Home Office, in accordance with the EU Directive 2010/63/EU.

#### 4-Methyl-umbelliferyloxymethylphosphonic acid diethylester (**1**)

A magnetically stirred suspension of sodium hydride (1.68 g, 60% dispersion in mineral oil, 42.0 mmol, 1.05 eq.) in dry DMSO 20 mL was cooled to 0 °C under nitrogen and a solution of 4-methyl-umbelliferone (7.05 g, 40.0 mmol, 1.00 eq.) in dry DMSO 20 mL was added dropwise. The foaming reaction mixture was allowed to warm to room temperature, stirred for a further 30 min until foaming had ceased and then diethyl tosyloxymethylphosphonate, DETOMP, (13.54 g, 42.0 mmol, 1.05 eq.) in 10 mL of dry DMSO was added dropwise. The reaction mixture was stirred overnight and then quenched in cold water 250 mL. The aqueous layer was extracted with ethyl acetate (3 × 100 mL) and the combined organic layers washed with saturated aqueous NaCl 100 mL, dried over magnesium sulfate and concentrated under reduced pressure to a yellow oil. The oil was purified by column chromatography on silica gel using ethyl acetate : hexane 1 : 1 then MeOH in ethyl acetate 1%. After removal of the solvent the pale-yellow oil crystallised on standing overnight. The crystals were dried under high vacuum to give 7.83 g of the titled compound **1** (24.0 mmol, 60%) as a white solid. M.p.: 96 °C; NSI-HRMS^+^ found: 327.100, C_15_H_20_O_6_P [M + H]^+^ requires 327.099; ^1^H NMR (400 MHz, CDCl_3_) *δ* ppm 7.50 (1H, d, *J* = 8.8 Hz, H5), 6.92 (1H, dd, *J* = 8.8, 2.5 Hz, H6), 6.85 (1H, d, *J* = 2.5 Hz, H8), 6.14 (1H, d, *J* = 1.0 Hz, H3), 4.30 (2H, d, *J* = 10.4 Hz, OCH_2_P), 4.17–4.27 (4H, m, OCH_2_CH_3_), 2.38 (3H, s, Ar–CH_3_), 1.35 (6H, t, *J* = 8.0 Hz, OCH_2_CH_3_); ^13^C NMR (101 MHz, CDCl_3_) *δ* ppm 161.48 (d, *J* = 13.9 Hz, C7), 161.05 (s, C2), 155.14 (s, C9), 152.40 (s, C4), 125.84 (s, C5), 114.60 (s, C10), 112.69 (s, C3), 112.33 (s, C6), 101.92 (s, C8), 63.17 (d, *J* = 6.6 Hz, OCH_2_CH_3_), 62.55 (d, *J* = 170.0 Hz, OCH_2_P), 18.77 (s, Ar–CH_3_), 16.57 (d, *J* = 5.1 Hz, OCH_2_CH_3_); ^31^P NMR (162 MHz, CDCl_3_) *δ* ppm 18.26 (s); IR (*ν*/cm^−1^) 2363, 1718 (C=O), 1615 (C=C), 1386 (CH_3_), 1319, 1291, 1237 (C–O), 1150 (P=O), 1016 (P–O), 971 (P–O), 842, 720, 568, 456. Predicted log *D* 2.303.

Crystal structure determination of compound **1**: Crystal data. C_15_H_19_O_6_P, *M* = 326.27, monoclinic, *a* = 16.6014(10), *b* = 8.812(2), *c* = 11.132(2) Å, *U* = 1572.2(5) Å^3^, *T* = 160 K, space group *P*2_1_/*n*, *Z* = 4, 3800 reflections measured, 3585 unique (*R*^int^ = 0.0130), which were used in all calculations. The final w*R*(*F*^2^) was 0.2212 (all data). CCDC no. 892006.†

#### 4-Methyl-umbelliferyloxymethylphosphonic acid ethyl ester (**2**)

A stirred solution of **1** (0.490 g, 1.50 mmol) in ethanol (25 mL) at 0 °C was treated dropwise with 2.0 M aqueous sodium hydroxide (7.5 mL). The stirred reaction mixture was left to reach room temperature and then held at 50 °C for 5 h. The ethanol was removed, the residual aqueous fraction cooled to 0 °C and acidified (pH ∼ 1) with aqueous HCl 2 M. Ethyl acetate 20 mL was added and the stirred biphasic mixture allowed to reach room temperature. The organic layer was separated and the aqueous layer extracted with ethyl acetate (3 × 20 mL). The combined organic layers were dried over magnesium sulfate then concentrated under vacuum to give 420 mg of the titled compound **2** (1.41 mmol, 94%). M.p.: 177 °C; NSI-HRMS^+^ found: 299.0676, C_13_H_16_O_6_P [M + H]^+^ requires 299.0685; ^1^H NMR (400 MHz, DMSO-*d*_6_) *δ* ppm 8.96 (1H, br. s), 7.79 (1H, d, *J* = 12.0 Hz, H5), 7.18 (1H, s, H8), 7.13 (1H, dd, *J* = 8.8, 2.5 Hz, H6), 6.32 (1H, d, s, H3), 4.48 (2H, d, *J* = 12.0 Hz, OCH_2_P), 4.16 (2H, m, OCH_2_CH_3_), 2.50 (3H, s, Ar–CH_3_), 1.34 (3H, t, *J* = 8.0 Hz, OCH_2_CH_3_); ^13^C NMR (101 MHz, DMSO-*d*_6_) *δ* 161.6 (d, *J* = 13.2 Hz, C7), 160.0 (C2), 154.6 (C9), 153.3 (C4), 126.4 (C5), 113.6 (C10), 112.4 (C3), 111.4 (C6), 101.5 (C8), 62.9 (d, *J* = 161 Hz, OCH_2_P), 61.4 (d, *J* = 10 Hz, OCH_2_CH_3_), 18.1 (Ar–CH_3_), 16.4 (d, *J* = 5.9 Hz, OCH_2_CH_3_). ^31^P NMR (162 MHz, DMSO-*d*_6_) *δ* ppm 15.85 (s); IR (*ν*/cm^−1^) 2325 (O–H), 1721 (C=O), 1609 (C=C), 1508 (C=C), 1389, 1371 (CH_3_), 1261 (O–H), 1156 (C–O), 1075 (P=O), 1002 (C–O), 955 (P–O), 840, 794, 748, 530, 441; *λ*_ex_ (PBS, nm) 328, *λ*_em_ 384. Predicted log *D* 0.461.

Crystal structure determination of compound **2**: Crystal data. C_13_H_15_O_6_P, *M* = 298.22, monoclinic, *a* = 7.0955(3), *b* = 24.2238(10), *c* = 8.2176(4) Å, *U* = 1353.27(10) Å^3^, *T* = 120(2) K, space group *P*2_1_/*c*, *Z* = 4, 10 427 reflections measured, 2846 unique (*R*^int^ = 0.0601), which were used in all calculations. The final w*R*(*F*^2^) was 0.1864 (all data). CCDC no. 892005.†

#### 3-[4-(Ethoxy-hydroxy-phosphorylmethoxy)-2-hydroxy-phenyl]-but-2-enoic acid (**2a**)

An aqueous ethanol solution of compound **1** was basified with 2 M NaOH and stirred at 70 °C for 5 h. The solvent was evaporated and the residual orange oil which crystallised on standing was identified as **2a**, Fig. 1, by single crystal X-ray crystallography (see Fig. 5 and S40–S43†).

Crystal structure determination of compound **2a**: Crystal data. C_13_H_20_Na_3_O_10_P, *M* = 436.23, monoclinic, *a* = 14.7973(8), *b* = 6.4554(3), *c* = 20.7665(11) Å, *U* = 1899.72(17) Å^3^, *T* = 100(2) K, space group *P*2_1_/*n*, *Z* = 4, 24 285 reflections measured, unique 4355 (*R*^int^ = 0.0448), which were used in all calculations. The final w*R*(*F*^2^) was 0.0828 (all data). CCDC no. 892004.†

#### 4-Methyl-umbelliferyloxymethylphosphonic acid (**2b**)

A stirred solution of **1** (0.36 g, 1.2 mmol) in dry dichloromethane (8 mL) under nitrogen was treated with trimethylsilyl iodide (0.85 mL, 5.6 mmol). The red solution was stirred 2 h, methanol (15 mL) added and solvents removed under reduced pressure after a further 2 h. Water (20 mL) was added to the residue and the aqueous mixture subsequently concentrated under reduced pressure. The last step was repeated four times to give **2b** as a white solid (0.26 g, 95%). M.p.: 185–187 °C; NSI-HRMS^−^ found: 269.0215 C_11_H_10_O_6_P [M − H]^−^ requires 269.0220; ^1^H NMR (400 MHz, DMSO-*d*_6_) *δ* ppm 8.01 (2H, br. s), 7.67 (1H, d, *J* = 8.0 Hz, H5), 6.96–7.08 (2H, m, H5, H8), 6.21 (1H, s, H3), 4.24 (2H, d, *J* = 12.0 Hz, OCH_2_P), 2.39 (3H, s, ArCH_3_). ^13^C NMR (101 MHz, DMSO-*d*_6_) *δ* ppm 161.8 (d, *J* = 13.2 Hz, C7), 160.1 (C2), 154.6 (C9), 153.3 (C4), 126.4 (C5), 113.5 (C10), 112.4 (C3), 111.4 (C6), 101.4 (C8), 64.0 (d, *J* = 163.6 Hz, OCH_2_P), 18.1 (ArCH_3_).^31^P NMR (162 MHz, DMSO-*d*_6_) *δ* ppm 13.91 (s); IR (*ν*/cm^−1^) 3400 (OH), 1677 (C=O), 1606 (C=C), 1392 (CH_3_), 1271 (OH), 1350, 1137, 1075 (P=O), 1018 (C–O), 857 (P–O), 830, 750, 520, 432. Predicted log *D* 0.14.

#### 3,4-Dimethyl-umbelliferyloxymethylphosphonic acid diethylester (**3**)

A stirred mixture of DETOMP (1.49 g, 4.63 mmol, 1.2 eq.) and dry DMSO 8 mL was treated successively with 3,4-dimethyl umbelliferone (0.73 g, 3.84 mmol, 1.0 eq.) prepared as described^29^ and potassium carbonate (0.637 g, 4.61 mmol, 1.20 eq.). The resultant yellow mixture was stirred at 50 °C overnight. The reaction was quenched with cold water (20 mL). After work up and purification in the manner described for **1** the title compound **3** 670 mg was obtained (1.96 mmol, 51%) as a white solid. M.p.: 90–94 °C; NSI-HRMS^+^ found: 341.1151, C_16_H_22_O_6_P [M + H]^+^ requires 341.1149; ^1^H NMR (400 MHz, CDCl_3_) *δ* ppm 7.52 (1H, d, *J* = 8.8 Hz, H5), 6.93 (1H, dd, *J* = 8.9, 2.6 Hz, H6), 6.86 (1H, d, *J* = 2.5 Hz, H8), 4.31 (2H, d, *J* = 10.3 Hz, OCH_2_P), 4.25 (4H, dq, *J* = 8.3, 7.0 Hz, OCH_2_CH_3_), 2.38 (3H, s, C_4_–CH_3_), 2.19 (3H, s, C_3_–CH_3_), 1.38 (6H, t, *J* = 7.0 Hz, OCH_2_CH_3_); ^13^C NMR (101 MHz, CDCl_3_) *δ* ppm 162.25 (s, C2), 160.38 (d, *J* = 13.9 Hz, C7), 153.40 (s, C9), 146.06 (s, C4), 125.54 (s, C5), 119.74 (s, C3), 115.21 (s, C10), 112.12 (s, C6), 101.61 (s, C8), 63.14 (d, *J* = 6.1 Hz, OCH_2_CH_3_), 62.48 (d, *J* = 170.0 Hz, OCH_2_P), 16.57 (d, *J* = 5.1 Hz, OCH_2_CH_3_), 15.20 (s, C_4_–CH_3_), 13.29 (s, C_3_–CH_3_); ^31^P NMR (162 MHz, CDCl_3_) *δ* ppm 18.46 (s); IR (*ν*/cm^−1^) 2990, 2953, 1706 (C=O), 1611 (C=C), 1509, 1448, 1446, 1386 (CH_3_), 1383, 1360, 1351, 1286, 1234 m (C–O), 1177 (P=O), 1175, 1145, 1071, 1022 (P–O), 979 (P–O), 972, 954, 854, 834, 821, 786, 760, 724. Predicted log *D* 2.769.

#### 3,4-Tetrahydrobenzo-umbelliferyloxymethylphosphonic acid diethylester (**4**)

Compound **4** was prepared as described for **3** from 3,4-tetrahydrobenzo-umbelliferone^29^ (0.658 g, 3.03 mmol, 1.0 eq.), potassium carbonate (0.505 g, 3.65 mmol, 1.20 eq.), DETOMP (1.18 g, 3.66 mmol, 1.2 eq.) and equivalent amounts of DMSO. The reaction afforded 690 mg of the titled compound **4** (1.88 mmol, 62%) as a white solid. M.p.: 114–118 °C; NSI-HRMS^+^ found: 367.1307, C_18_H_24_O_6_P [M + H]^+^ requires 367.1305; ^1^H NMR (400 MHz, CDCl_3_) *δ* ppm 7.46 (1H, d, *J* = 8.8 Hz, H5), 6.89 (1H, dd, *J* = 8.7, 2.4 Hz, H6), 6.83 (1H, d, *J* = 2.5 Hz, H8), 4.29 (2H, d, *J* = 10.4 Hz, OCH_2_P), 4.17–4.27 (4H, m, OCH_2_CH_3_), 2.68–2.77 (2H, m, C_3_–CH_2_CH_2_), 2.49–2.57 (2H, m, C_4_–CH_2_CH_2_), 1.73–1.87 (4H, m, C_3_–CH_2_CH̲_2_CH̲_2_CH_2_–C_4_), 1.36 (6H, t, *J* = 7.1 Hz, OCH_2_CH_3_); ^13^C NMR (101 MHz, CDCl_3_) *δ* ppm 161.97 (s, C2), 160.29 (d, *J* = 14.6 Hz, C7), 153.38 (s, C9), 147.11 (s, C4), 124.41 (s, C5), 121.35 (s, C3), 114.81 (s, C10), 112.03 (s, C6), 101.68 (s, C8), 63.14 (d, *J* = 6.6 Hz, OCH_2_CH_3_), 62.50 (d, *J* = 170.0 Hz, OCH_2_P), 25.31 (s, C_3_–C̲H_2_CH_2_), 23.95 (s, C_4_–C̲H_2_CH_2_), 21.71 (s, C_3_–CH_2_C̲H_2_CH_2_CH_2_–C_4_), 21.42 (s, C_3_–CH_2_CH_2_C̲H_2_CH_2_–C_4_), 16.58 (d, *J* = 6.1 Hz, OCH_2_CH_3_); ^31^P NMR (162 MHz, CDCl_3_) *δ* ppm 18.50 (s); IR (*ν*/cm^−1^) 3000, 2948, 1717 (C=O), 1606 (C=C), 1389 (CH_3_), 1277 (C–O), 1247, 1167, 1168 (P=O), 1017 (P–O), 963 (P–O), 830, 802, 727, 636. Predicted log *D* 3.31.

#### 4-Phenyl-umbelliferyloxymethylphosphonic acid diethylester (**5**)

Compound 5 was prepared in the manner described for **3** from 4-phenyl-umbelliferone (0.467 g, 1.96 mmol, 1.0 eq.), potassium carbonate (0.304 g, 2.20 mmol, 1.12 eq.), DETOMP (0.774 g, 2.40 mmol, 1.2 eq.) and equivalent amounts of DMSO. The title compound **5** 323 mg was obtained as a white solid (0.82 mmol, 42%). M.p.: 78–82 °C; NSI-HRMS^+^ found: 389.1149, C_20_H_22_O_6_P [M + H]^+^ requires 389.1149; ^1^H NMR (400 MHz, CDCl_3_) *δ* ppm 7.47–7.53 (3H, m, H5 and HPh), 7.38–7.45 (3H, m, HPh), 6.94 (1H, d, *J* = 2.5 Hz, H8), 6.86 (1H, dd, *J* = 8.8, 2.5 Hz, H6), 6.23 (1H, s, H3), 4.32 (2H, d, *J* = 10.4 Hz, OCH_2_P), 4.19–4.29 (4H, m, OCH_2_CH_3_), 1.37 (6H, t, *J* = 7.1 Hz, OCH_2_CH_3_); ^13^C NMR (101 MHz, CDCl_3_) *δ* ppm 161.64 (d, *J* = 13.9 Hz, C7), 161.01 (s, C2), 155.86 (s, C9), 155.68 (s, C4), 135.46 (s, CPh), 129.79 (s, CPh), 128.98 (s, CPh), 128.46 (s, CPh), 128.30 (s, C6), 113.57 (s, C3), 112.64 (s, C10), 112.39 (s, C6), 102.18 (s, C8), 63.20 (d, *J* = 6.6 Hz, OCH_2_CH_3_), 62.60 (d, *J* = 170.0 Hz, OCH_2_P), 16.60 (d, J = 5.1 Hz, OCH_2_CH_3_); ^31^P NMR (162 MHz, CDCl_3_) *δ* ppm 18.19 (s); IR (*ν*/cm^−1^) 3088, 2995, 2920, 1727 (C=O), 1613 (C=C), 1374 (CH_3_), 1294 m, 1256 (C–O), 1152 (P=O), 1146, 1047, 1020 (P–O), 973 (P–O), 850, 779, 713, 712. Predicted log *D* 3.355.

#### 3-Chloro-4-methyl-umbelliferyloxymethylphosphonic acid diethylester (**6**)

Compound **6** was prepared in the manner described for **3** from 3-chloro-4-methyl-umbelliferone (0.210 g, 1.00 mmol, 1.0 eq.), potassium carbonate (0.152 g, 1.10 mmol, 1.10 eq.), DETOMP (0.387 g, 1.20 mmol, 1.2 eq.) and equivalent amounts of DMSO. The title compound **6** 235 mg was obtained as a white solid (0.65 mmol, 65%). M.p.: 108–112 °C; NSI-HRMS^+^ found: 361.0606, C_15_H_19_ClO_6_P [M + H]^+^ requires 361.0602; ^1^H NMR (400 MHz, CDCl_3_) *δ* ppm 7.53 (1H, d, *J* = 8.8 Hz, H5), 6.97 (1H, dd, *J* = 9.0, 2.7 Hz, H6), 6.87 (1H, d, *J* = 2.5 Hz, H8), 4.31 (2H, d, *J* = 10.1 Hz, OCH_2_P), 4.17–4.28 (4H, m, OCH_2_CH_3_), 2.53 (3H, s, Ar–CH_3_), 1.36 (6H, t, *J* = 7.1 Hz, OCH_2_CH_3_); ^13^C NMR (101 MHz, CDCl_3_) *δ* ppm 161.38 (d, *J* = 13.9 Hz, C7), 157.24 (s, C2), 152.96 (s, C9), 147.81 (s, C4), 126.19 (s, C5), 118.57 (s, C3), 114.31 (s, C10), 113.05 (s, C6), 101.88 (s, C8), 63.21 (d, *J* = 6.6 Hz, OCH_2_CH_3_), 62.64 (d, *J* = 170.0 Hz, OCH_2_P), 16.59 (d, *J* = 5.1 Hz, OCH_2_CH_3_), 16.31 (s, Ar–CH_3_); ^31^P NMR (162 MHz, CDCl_3_) *δ* ppm 18.07 (s); IR (*ν*/cm^−1^) 2991, 2915, 1736 (C=O), 1616 (C=C), 1557, 1516, 1391 (CH_3_), 1296, 1252 m (C–O), 1157 (P=O), 1022, 1021 (P–O), 978 (P–O), 950, 943, 864, 824, 775, 752, 727, 625. Predicted log *D* 2.692.

#### Diethyl 7-oxymethylphosphonate-4-methylquinolin-2(1*H*)-one (**7**)

7-Hydroxy-4-methylquinolin-2(1*H*)-one^30^ (0.350 g, 2.00 mmol) in DMSO 4 mL was combined with sodium hydride (0.05 g, 2.10 mmol) under nitrogen and the reaction mixture stirred for 30 min until generation of hydrogen gas bubbles ceased. DETOMP (0.77 g, 2.39 mmol) was added, the mixture was stirred under N_2_ at 50 °C for 6 h then cooled and quenched with distilled water. The aqueous layer was extracted with ethyl acetate (3 × 6 ml) and the combined organic layers, dried and concentrated. Column chromatography of the crude product on silica gel eluted biproduct fractions from ethyl acetate : MeOH (8 : 2) and 570 mg of slightly impure (by ^31^P NMR) product from ethyl acetate : MeOH (9 : 1). This was further purified on a second column using DCM : MeOH 98 : 2 followed by DCM : MeOH 95 : 5 to give the title compound **7** (0.10 g, 0.31 mmol, 15.4%) as a white powder. M.p.: 163–165 °C; NSI-HRMS^+^ found: 326.1146 C_15_H_21_NO_5_P [M + H]^+^ requires 326.1152; ^1^H NMR (400 MHz, CDCl_3_) *δ* ppm 12.62 (1H, br. s., NH), 7.58 (1H, d, *J* = 8.6 Hz, H5), 6.74–6.94 (2H, m, H6 and H8), 6.46 (1H, s, H3), 4.29 (2H, d, *J* = 10.4 Hz, OCH_2_P), 4.25 (4H, m, CH_3_CH_2_O), 2.47 (3H, s, CH_3_), 1.38 (6H, t, *J* = 7.1 Hz, CH_3_CH_2_O); ^13^C NMR (101 MHz, CDCl_3_) *δ* ppm 164.95 (s, C2), 160.61 (s, C7), 149.2 (s, C4), 140.1 (s, C9), 126.1 (s, C5), 118.3 (s, C3), 115.7 (s, C10), 111.9 (s, C8), 99.7 (s, C6), 63.17 (d, *J* = 6.6 Hz, CH_3_CH_2_O), 62.35 (d, *J* = 164.7 Hz, OCH_2_P), 19.30 (s, CH_3_), 16.63 (d, *J* = 5.9 Hz, CH_3_CH_2_O); ^31^P NMR (162 MHz, CDCl_3_) *δ* ppm 18.82 (s); *λ*_ex_ (ethanol, nm) 340, *λ*_em_ 365; IR (*ν*/cm^−1^) 2985, 2902, 2830, 1615 (C=O), 1556, 1523, 1300 (P=O), 1165 (P–O), 810, 800. Predicted log *D* 1.676.

#### 4-Methyl-umbelliferylthiomethylphosphonic acid diethylester (**8**)

Compound **8** was prepared in the manner described for **3** from 7-mercapto-4-methyl-umbelliferone (0.576 g, 3.00 mmol, 1.0 eq.) potassium carbonate (0.456 g, 3.3 mmol, 1.10 eq.), DETOMP (1.16 g, 3.6 mmol, 1.2 eq.) and equivalent amounts of DMSO. The title compound **8** 731 mg was obtained as a white solid (2.14 mmol, 71%) M.p.: 64–66 °C; NSI-HRMS^+^ found: 343.0761 C_15_H_20_O_5_PS [M + H]^+^ requires 343.0764; ^1^H NMR (400 MHz, CDCl_3_) *δ* ppm 7.49 (1H, d, *J* = 8.1 Hz, H5), 7.28 (1H, s, H8), 7.25 (1H, d, *J* = 8 Hz, H6), 6.23 (1H, s, H3), 4.17 (4H, quin, *J* = 8.0 Hz, OCH_2_CH_3_), 3.24 (2H, d, *J* = 16.0 Hz, SCH_2_P), 2.40 (3H, s, ArCH_3_), 1.32 (6H, t, *J* = 8.0 Hz, OCH_2_CH_3_); ^13^C NMR (101 MHz, CDCl_3_) *δ* ppm 160.46 (s, C2), 153.81 (s, C4), 152.04 (s, C9), 141.55 (d, *J* = 5.9 Hz, C7), 124.87 (s, C5), 123.49 (s, C6), 118.03 (s, C10), 115.24 (s, C8), 114.53 (s, C3), 63.11 (d, *J* = 6.6 Hz, OCH_2_CH_3_), 27.24 (d, *J* = 151.5 Hz, SCH_2_P), 18.65 (s, Ar–CH_3_), 16.51 (d, *J* = 5.9 Hz, OCH_2_CH_3_); ^31^P NMR (162 MHz, CDCl_3_) *δ* ppm 21.90 (s); IR (*ν*/cm^−1^) 2989, 2914, 1737 (C=O), 1725, 1616 (C=C), 1380 (CH_3_), 1296, 1253 (C–O), 1227, 1157 (P=O), 1055, 1023 (P–O), 955 (P–O), 824, 774, 769, 626. Predicted log *D* 2.881.

### Preparation of ADDL and fibrillated β-amyloid

ADDLs stock 100 μM in neurobasal medium without Phenol Red from Aβ42 was freshly prepared and used as previously described.^6^ Fibrillated Aβ (Fig. 9(a)) was prepared by incubating the same 100 μM stock at 37 °C with constant agitation for 72 h (for details see S52†).

### Hippocampal cell cultures

Primary hippocampal neuronal cells were isolated from adult Sprague-Dawley rat dams (200–250 g) at embryonic day 18 (E18). Individual hippocampi were dissected and stored in ice-cold Hanks balanced salt solution (HBBS) without Mg^2+^/Ca^2+^ and supplemented with sodium bicarbonate (4.2 mM). The tissue was then incubated in 0.05% trypsin at 37 °C for 10 min. The HBBS was then removed and replaced with Neurobasal medium without Phenol red, containing 5% fetal bovine serum (FBS). The trypsinised tissue was then triturated using a fire polished Pasteur pipette and centrifuged for 5 min at 500 g. The supernatant was removed and replaced with Neurobasal medium without Phenol red, supplemented with 0.5 mM glutamine, 2% B-27 supplement, 2% penicillin/streptomycin. Cells were plated on poly-d-lysine coated 96-well plates at a density of 1.52 × 10^5^ cells per well and maintained at 37 °C with 5% CO_2_. Half of the medium was changed every 3 days. On day 3 *in vitro* (DIV 3) 5 μM of cytosine arabinoside (ara-C) was added to the medium to prevent proliferation of non-neuronal cells. At DIV 7, the B-27 supplement was replaced with B-27 minus anti-oxidants supplement.

### Compound treatments of hippocampal cultures

Compounds 20 mM in DMSO were diluted to 100 μM with Neurobasal medium without Phenol red and filter sterilised. The following cell treatments were applied at DIV 7 (a) vehicle DMSO (0.1%) and in seperate experiments compounds **1–8** (1 μM); (b) ADDLs (5 μM), fibrils (5 μM), (c) ADDLs (5 μM) with compounds (1 μM) for **1–6** and **8**; (d) fibrils (5 μM) with compounds (1 μM) for **1–6** and **8**. Cells were incubated in their respective treatments for 72 h. Each treatment was performed in triplicate and repeated in three biologically independent experiments.

### MTT cell viability assay

A 5 mg mL^−1^ stock solution of 3-(4,5-dimethylthiazol-2-yl)-2,5-diphenyltetrazolium bromide (MTT) was prepared with phosphate buffered saline (PBS) and filter sterilized. The MTT solution was added to the medium to reach a final concentration of 0.5 mg mL^−1^ and incubated at 37 °C for 4 h. MTT, a yellow-coloured solution, is reduced to purple formazan in living cells. After 4 h incubation, the medium was removed and the purple formazan was solubilised by adding a solvent (absolute isopropanol containing 0.04 M HCl and 10% Triton X-100) and placed on an orbital shaker overnight to ensure the converted dye completely dissolved. Absorbance was measured using a plate reader at 570 nm and 690 nm and data expressed relative to DMSO vehicle control.

### Zinc signaling

At DIV 8, hippocampal cells were washed with a Tyrode solution then incubated for 30 min in Tyrode solution containing 5 μM FluoZin-3 in darkness. Cells were washed twice and incubated for 20 min in Tyrode solution. Cells were then incubated with compound **1** 1 μM or vehicle DMSO for 10 min. Fluorescence was imaged at Ex/Em = 494/516 every 2 min for 60 min with baseline values recorded in the first 6 min and addition of ZnCl_2_ 30 μM at 6 min. Images were processed with Metamorph™ software.

### Compound assisted partition of Zn^2+^ from water to octanol by furnace AAS measurements

#### Calibration

Calibration curves were generated for zinc and copper using standards for AAS and ultrapure water (see S47–S51†). For zinc in water, three different dilution regimes were run and no inconsistencies due to dilution were observed. Five measurements were made at each metal concentration. Further calibration curves for zinc in octanol saturated water and zinc in octanol saturated water spiked with compound **1** in DMSO were developed to rule out matrix effects on analyte absorbance. The aqueous metal calibration curves were used in partition experiments. Working solution concentrations were adjusted to account for trace zinc present in the ultrapure water.

#### Partition experiments

(i) Concentration dependent Zn^2+^ partition in aqueous–octanol 1 : 1 assisted by compound **1**.

A series of solutions **A** of Zn^2+^ in water, 10 concentrations, were prepared from which series **B** (1 mL in 1.5 mL microtubules) of Zn^2+^ in aqueous–octanol 1 : 1 and series **C** Zn^2+^ aqueous spiked with solution of compound **1** in DMSO to give 4 μM **1** and 0.01% DMSO and **D** Zn^2+^ aqueous–octanol 1 : 1 spiked with solution of compound **1** in DMSO to give 4 μM **1** and 0.01% DMSO were prepared in triplicate. Zn^2+^ concentrations were 0.5 μM, 0.8 μM and 1 μM–8 μM (10 samples in each). The test ratio **1**:Zn^2+^ in **C** and **D** covered the range 0.5–8.0. All test samples were agitated in a shaker at room temperature for 24 h. The Zn^2+^ concentration in series **A**, **C** and aqueous layers of **B** and **D** of each sample was analysed with five samples measured for each concentration. The % Zn^2+^ partitioned to octanol in series **B** and **D** was determined with reference to **A** and **C**, respectively. Data presented for % Zn^2+^ partitioned assisted by compound **1** less the media only values is shown in Fig. 7a.

(ii) Zn^2+^ 0.25 μM partition in aqueous–octanol 1 : 1 assisted by compound **1**, **3**, **4**, **5**, **6** and Clioquinol at 1 μM.

Samples of Zn^2+^ 0.25 μM in aqueous–octanol 1 : 1 (1 mL in 1.5 mL microtubules-reference samples) and separate experimental samples spiked with **1**, **3**, **4**, **5**, **6** or Clioquinol in DMSO to give 1 μM compound were prepared in triplicate. A fresh reference sample was prepared for each experiment. The biphasic samples were treated and Zn^2+^ measured as described in (i). The % Zn partitioned in the experimental samples was determined by comparison with the reference samples and data presented represents % Zn^2+^ partitioned assisted by compound less the media only values.

(iii) Cu^2+^ 1 μM partition in aqueous–octanol 1 : 1 assisted by compound **1** 4 μM.

Reference samples of Cu^2+^ 1 μM in aqueous–octanol 1 : 1 (1 mL in 1.5 mL microtubules) and separate experimental samples spiked with **1** in DMSO to give 4 μM compound were prepared in triplicate. A fresh reference sample was prepared for each experiment. The biphasic samples were treated and Cu^2+^ measured as described for Zn^2+^ in (i). The %Cu partitioned in the experimental samples was determined with respect to the reference samples.

## Author contributions

Authors were involved in conception, design, experimental, interpretation and assembly of data to various extents.

## Conflicts of interest

There are no conflicts to declare.

## Supplementary Material

DT-050-D1DT02298A-s001

DT-050-D1DT02298A-s002
