# Supplementary material for: Umbelliferyloxymethyl phosphonate compounds-weakly binding zinc ionophores with neuroprotective properties
Source: Dalton Trans. 2021 Nov 4;50(46):17041–51. doi: 10.1039/d1dt02298a (PMC8631114; doi:10.1039/d1dt02298a)
Supplement: DT-050-D1DT02298A-s001 [file DT-050-D1DT02298A-s001.pdf]

Electronic Supplementary Material (ESI) for Dalton Transactions.
This journal is © The Royal Society of Chemistry 2021

Umbelliferyloxymethyl Phosphonate Compounds-weakly binding zinc ionophores with neuroprotective properties

Sebastien Guesne,[a †] Laura Connole,[b †] Stephanie Kim,[a] Majid Motevalli,[a] Lesley Robson,[b] Adina T. Michael-Titus[b §] and Alice Sullivan[a §*]

Supporting Information

* Corresponding author, †Joint first authors, § AS PI Chemistry, ATMT PI Biology

Table of Contents

**Synthetic Schemes for compound synthesis**	**S3-S5**
**NMR, High Resolution Mass Spectrometry and IR Spectra**	**S6-S39**
Compound 1	S6-S8
Compound 2	S9-S12
Compound 2b	S13-S16
Compound 3	S17-S21
Compound 4	S22-S26
Compound 5	S27-S29
Compound 6	S30-S32
Compound 7	S33-S36
Compound 8	S37-S39
**Single Crystal X-ray Structural Data for compounds 1, 2, 2a**	**S40-S43**
**Fluorescence and UV-Vis titrations 1 with zinc**	**S44**
**^{31}P NMR titration of compounds 1 and 2 with Zn^{2+}**	**S45-S46**
**AAS instrument reports for Zinc and Copper calibration**	**S47-S51**
**Procedure for preparation of ADDLs and fibrillated Aβ(42)**	**S52**

Synthetic Schemes for compound synthesis

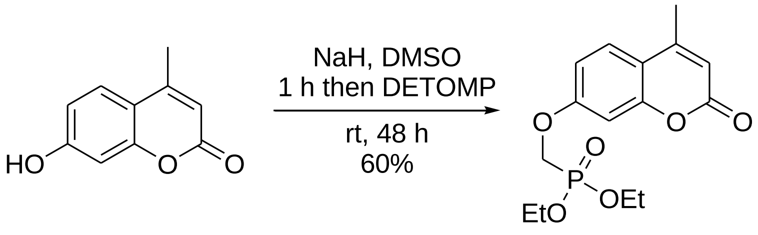

Umbelliferone **1**

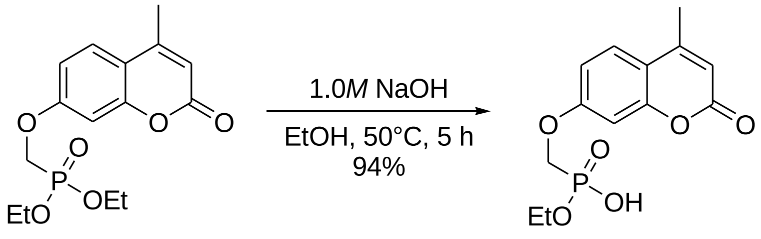

1 **2**

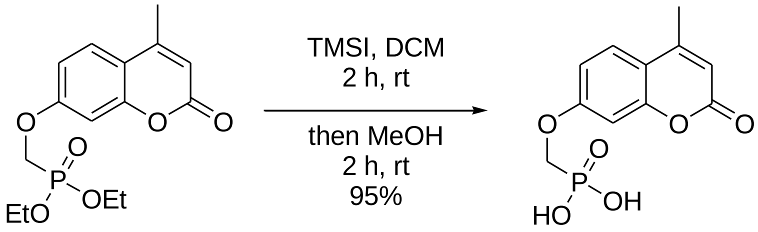

1 **2b**

Other analogues of **1** were synthesised as described below.

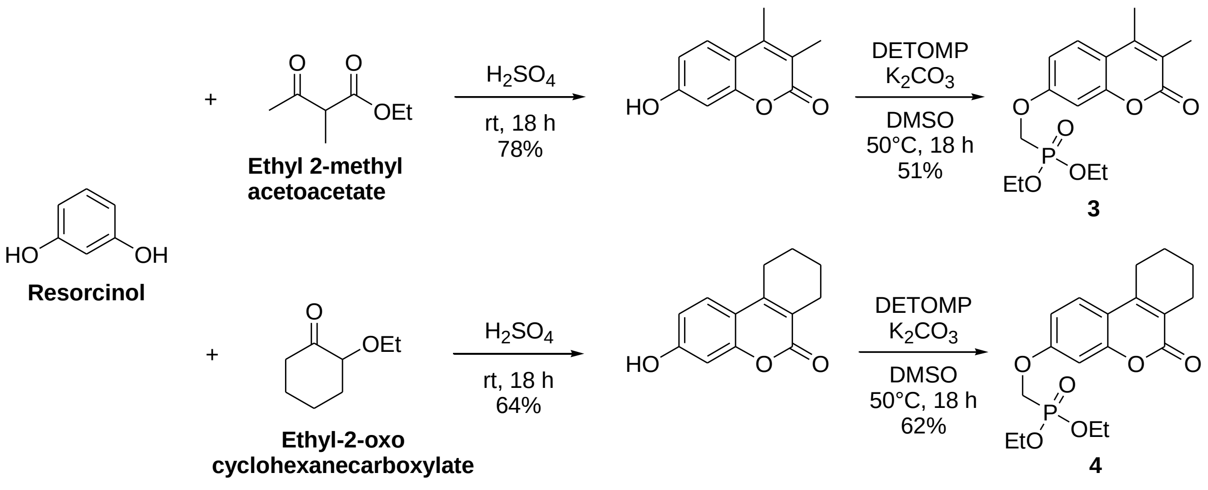
Resorcinol
Ethyl 2-methyl acetoacetate
H_2SO_4
rt, 18 h
78%
DETOMP
K_2CO_3
DMSO
50°C, 18 h
51%
3
Ethyl-2-oxo cyclohexanecarboxylate
H_2SO_4
rt, 18 h
64%
DETOMP
K_2CO_3
DMSO
50°C, 18 h
62%
4

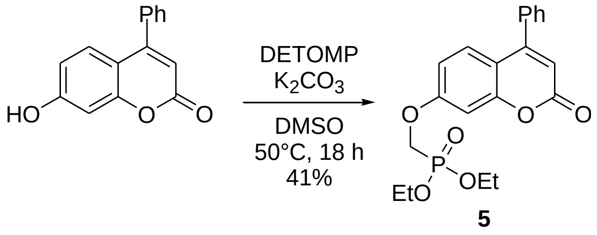
DETOMP
K_2CO_3
DMSO
50°C, 18 h
41%
5

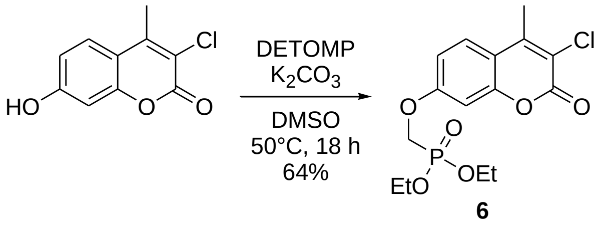
DETOMP
K_2CO_3
DMSO
50°C, 18 h
64%
6

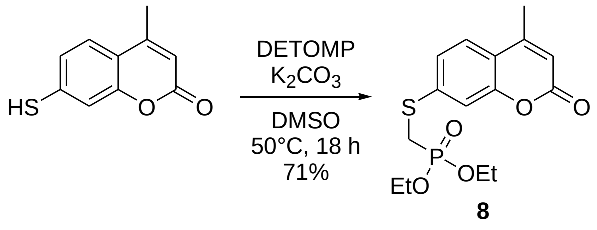
DETOMP
K_2CO_3
DMSO
50°C, 18 h
71%
8

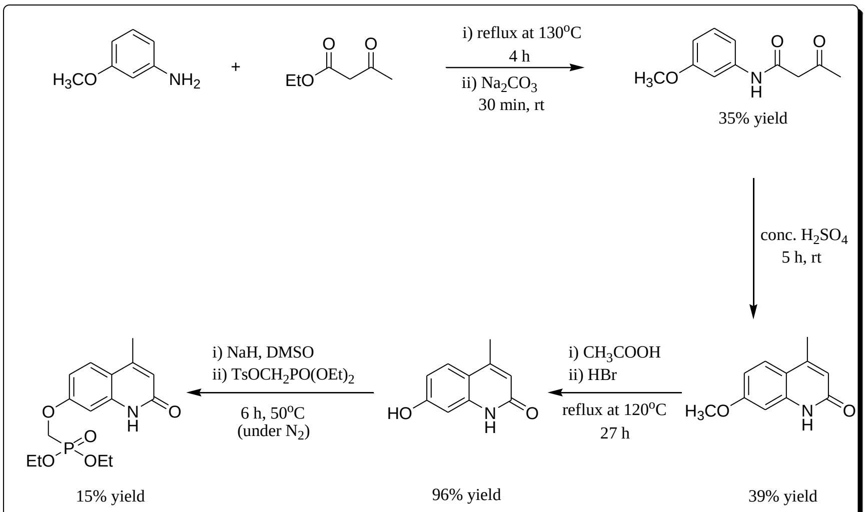

H3CO
NH2
+
EtO
O
O
i) reflux at 130oC
4 h
ii) Na2CO3
30 min, rt
H3CO
N
H
35% yield
conc. H2SO4
5 h, rt
H3CO
39% yield
i) CH3COOH
ii) HBr
reflux at 120oC
27 h
HO
96% yield
i) NaH, DMSO
ii) TsOCH2PO(OEt)2
6 h, 50oC
(under N2)
P
EtO
OEt
15% yield

7

NMR Spectra and High Resolution Mass Spectrometry

Spectra for Compound 1

a1) ^{1}H nmr

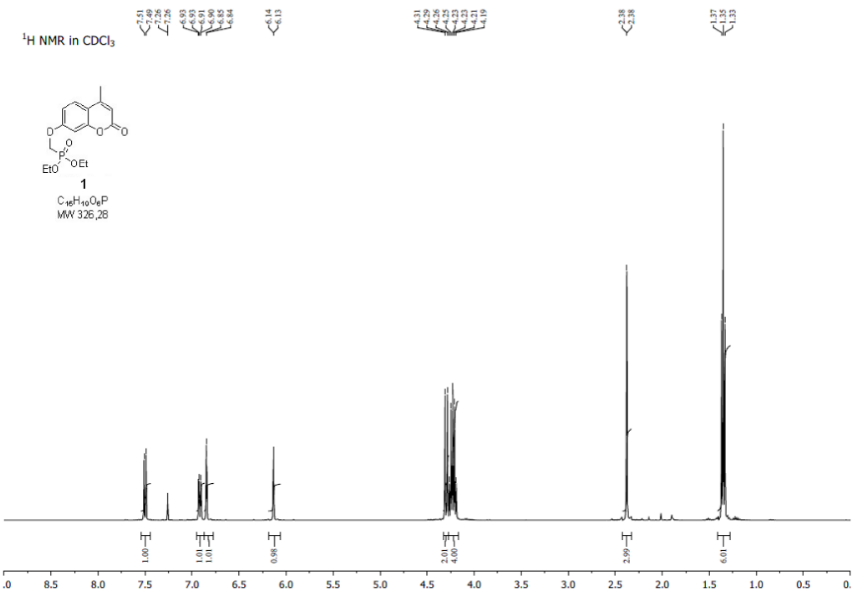

b1) ^{31}P NMR

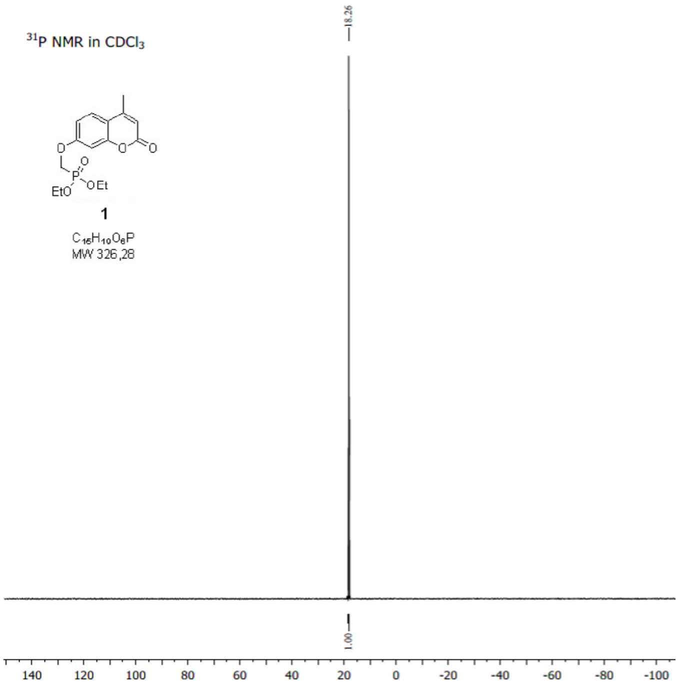

c1) ^{13}C NMR

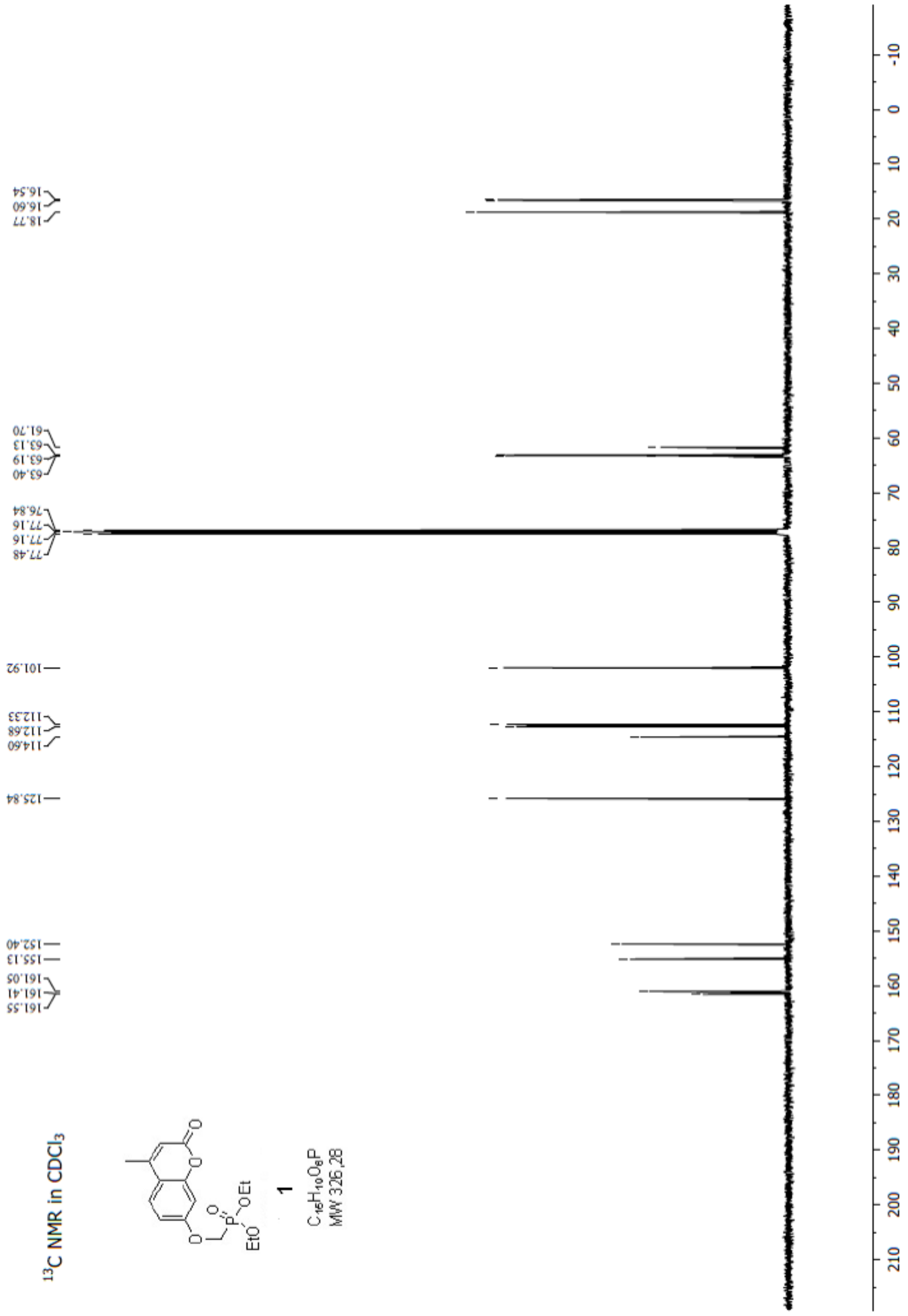

13C NMR in CDCl3
1
MW 326.28
16.54
16.60
18.77
61.70
63.13
63.19
63.40
76.84
77.16
77.48
101.92
112.33
112.68
114.60
125.84
152.40
155.13
161.05
161.41
161.55

d1) Mass spec

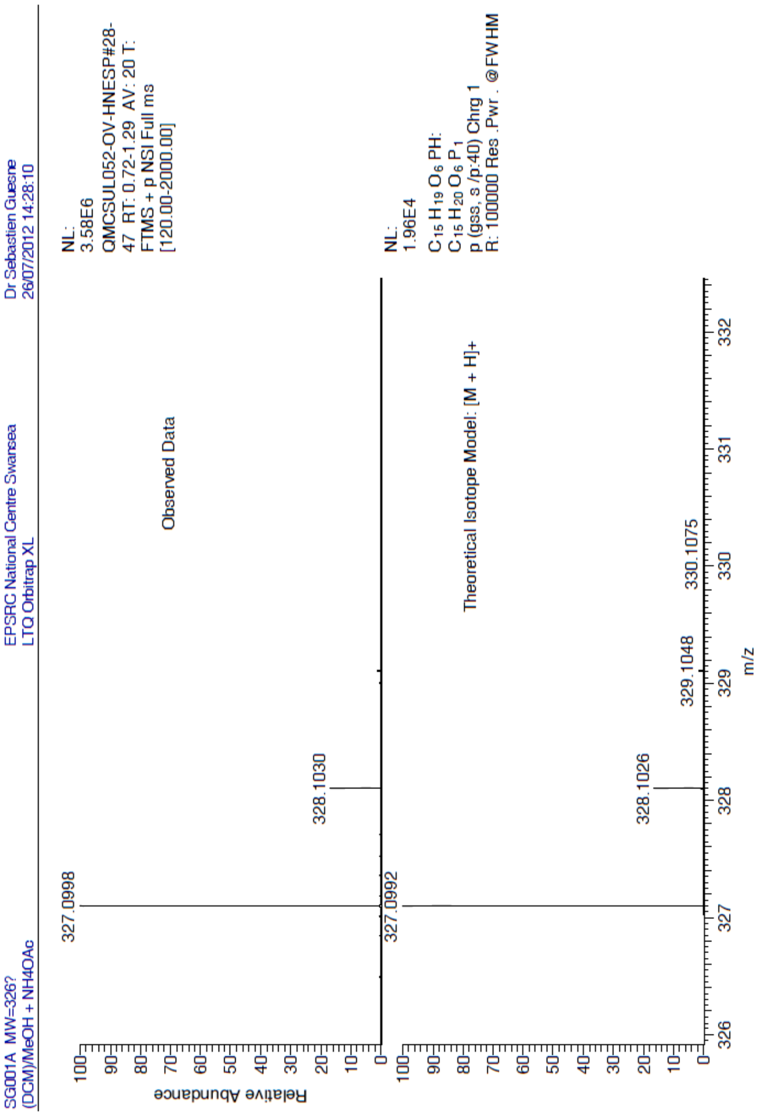

e1) IR spectrum

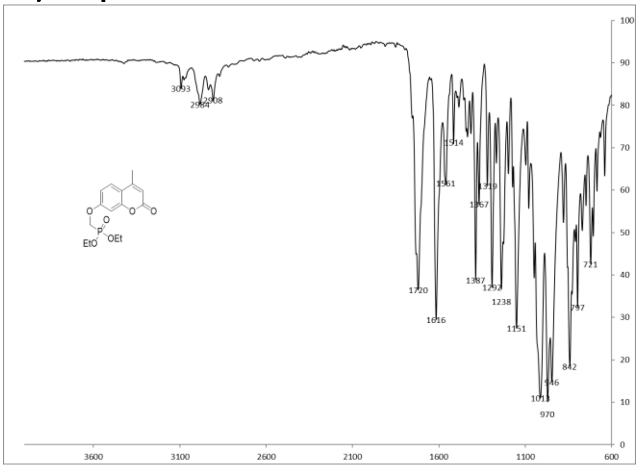

Spectra for Compound 2

a2) ^{1}H NMR

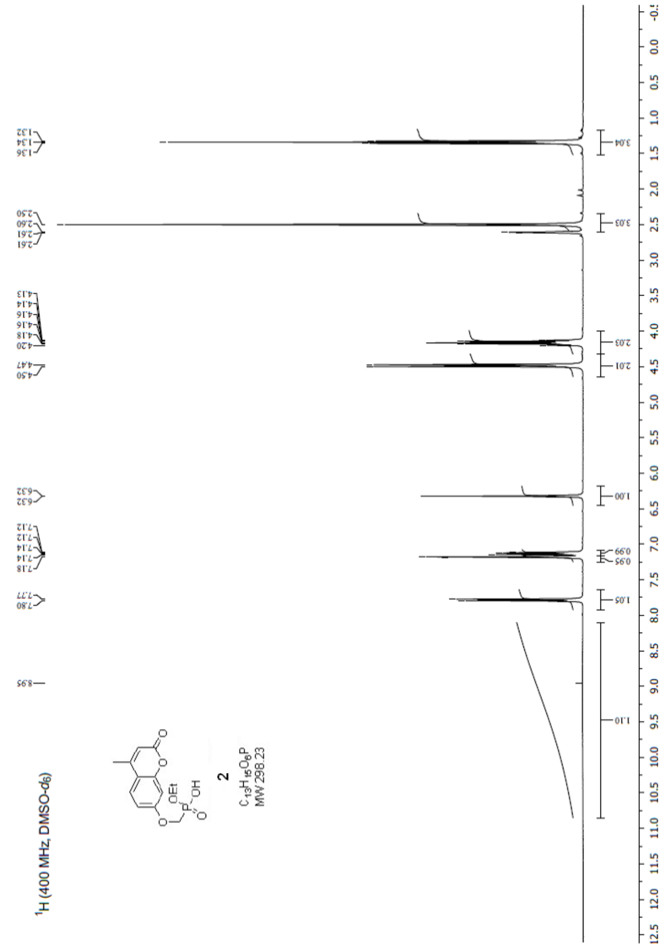

b2) ^{31}P NMR

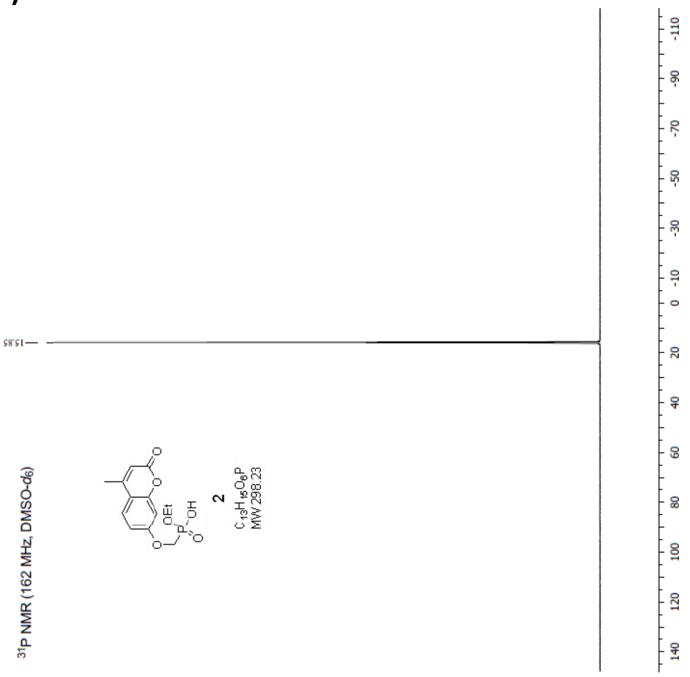

c2) ^{13}C NMR

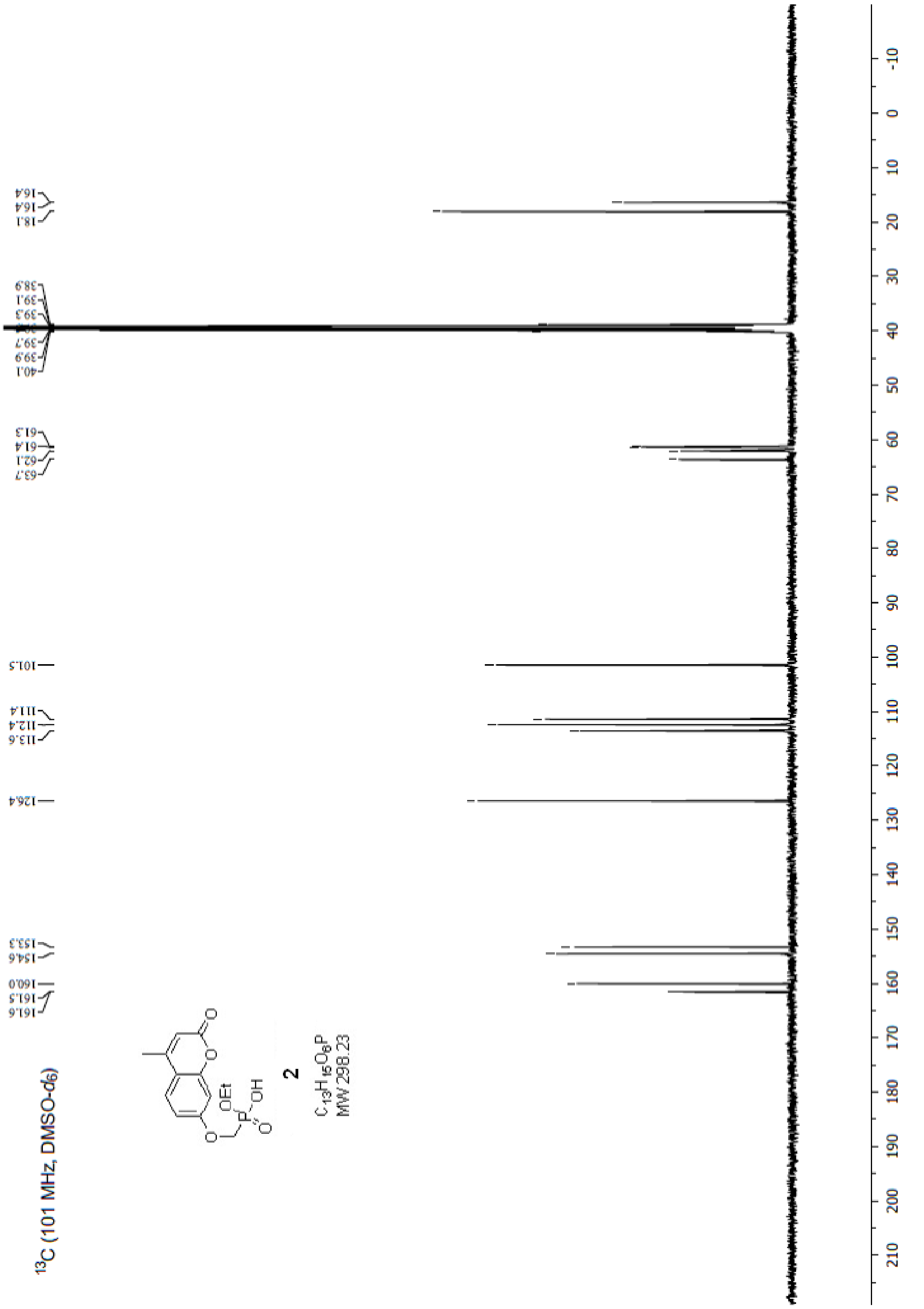

13C (101 MHz, DMSO-d6)
2
C13H15O6P
MW 298.23
OEt
OH
16.4
16.4
18.1
38.9
39.1
39.3
39.7
39.9
40.1
61.3
61.4
62.1
63.7
101.5
111.4
112.4
113.6
126.4
153.3
154.6
160.0
161.5
161.6
-10
0
10
20
30
40
50
60
70
80
90
100
110
120
130
140
150
160
170
180
190
200
210

d2) Mass spec

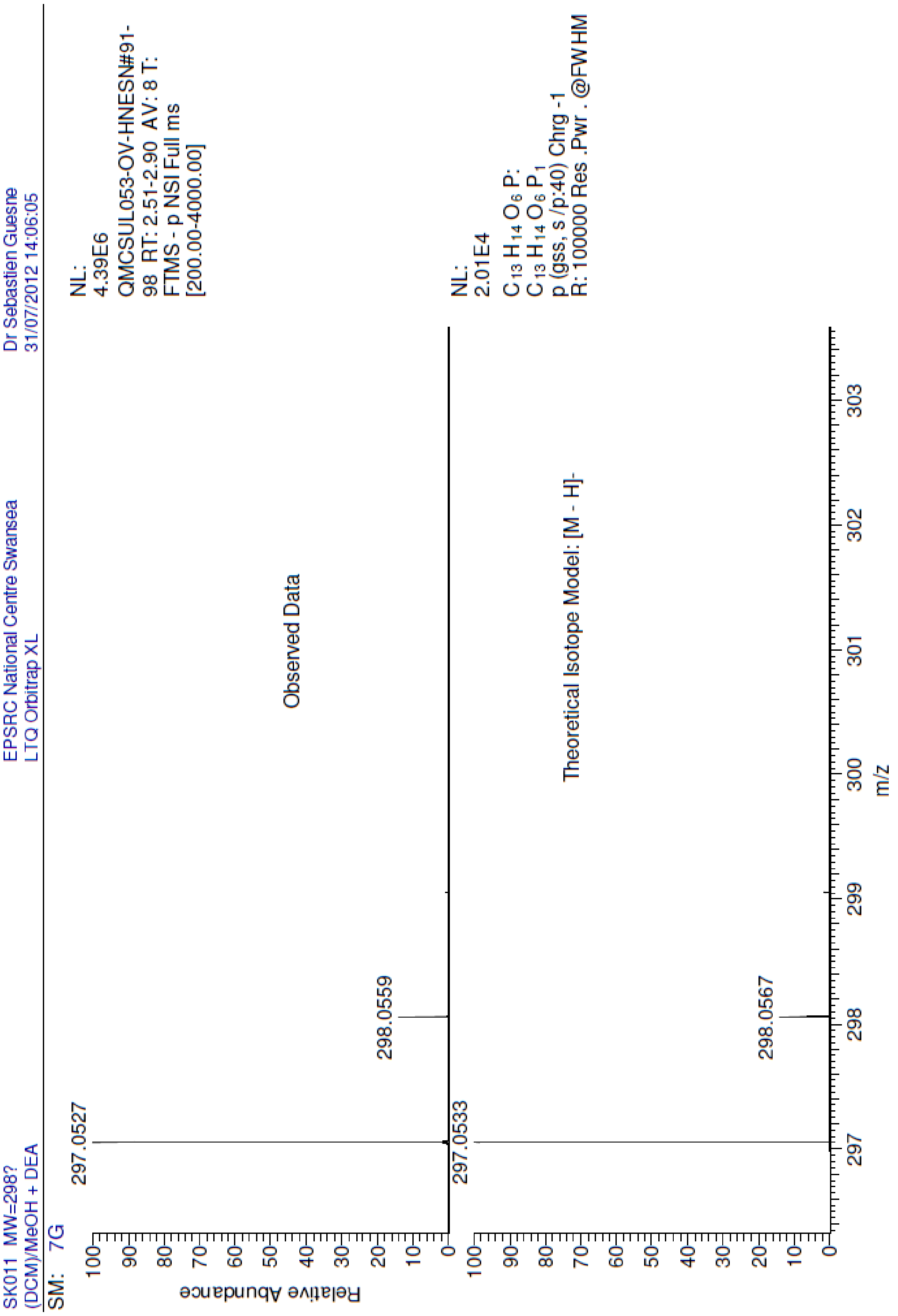
SK011 MW=298?
(DCM)/MeOH + DEA
SM: 7G
EPSRC National Centre Swansea
LTQ Orbitrap XL
Dr Sebastien Guesne
31/07/2012 14:06:05
NL:
4.39E6
QMCSUL053-OV-HNESN#91-98 RT: 2.51-2.90 AV: 8 T: FTMS - p NSI Full ms [200.00-4000.00]
Observed Data
297.0527
298.0559
NL:
2.01E4
C 13 H 14 O 6 P:
C 13 H 14 O 6 P 1
p (gss, s /p:40) Chrg -1
R: 100000 Res .Pwr . @FWHM
Theoretical Isotope Model: [M - H]-
297.0533
298.0567
Relative Abundance
m/z

e2) IR spectrum

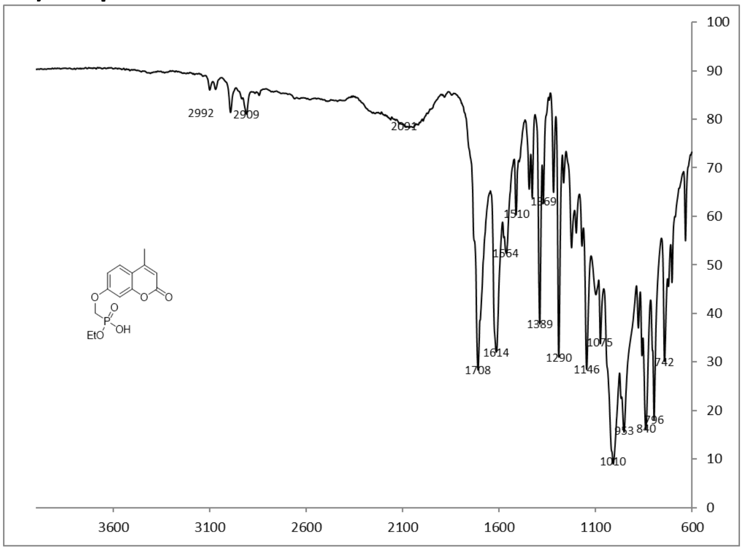

Spectra for Compound 2b

a2b) ^{1}H NMR

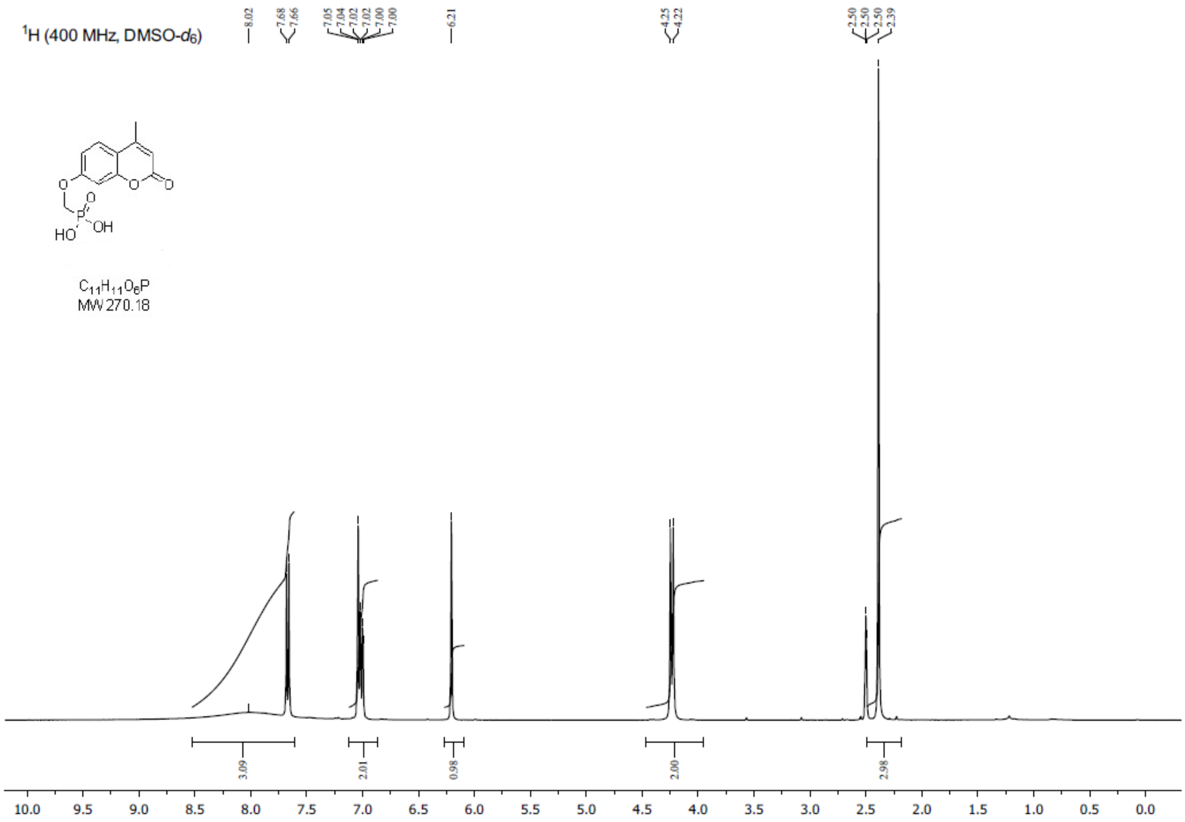

b2b) ^{31}P NMR

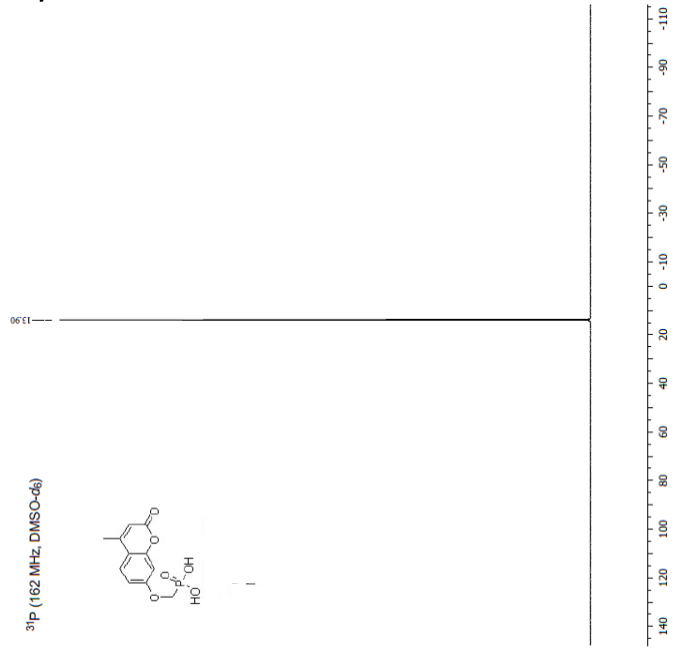

c2b) ^{13}C NMR

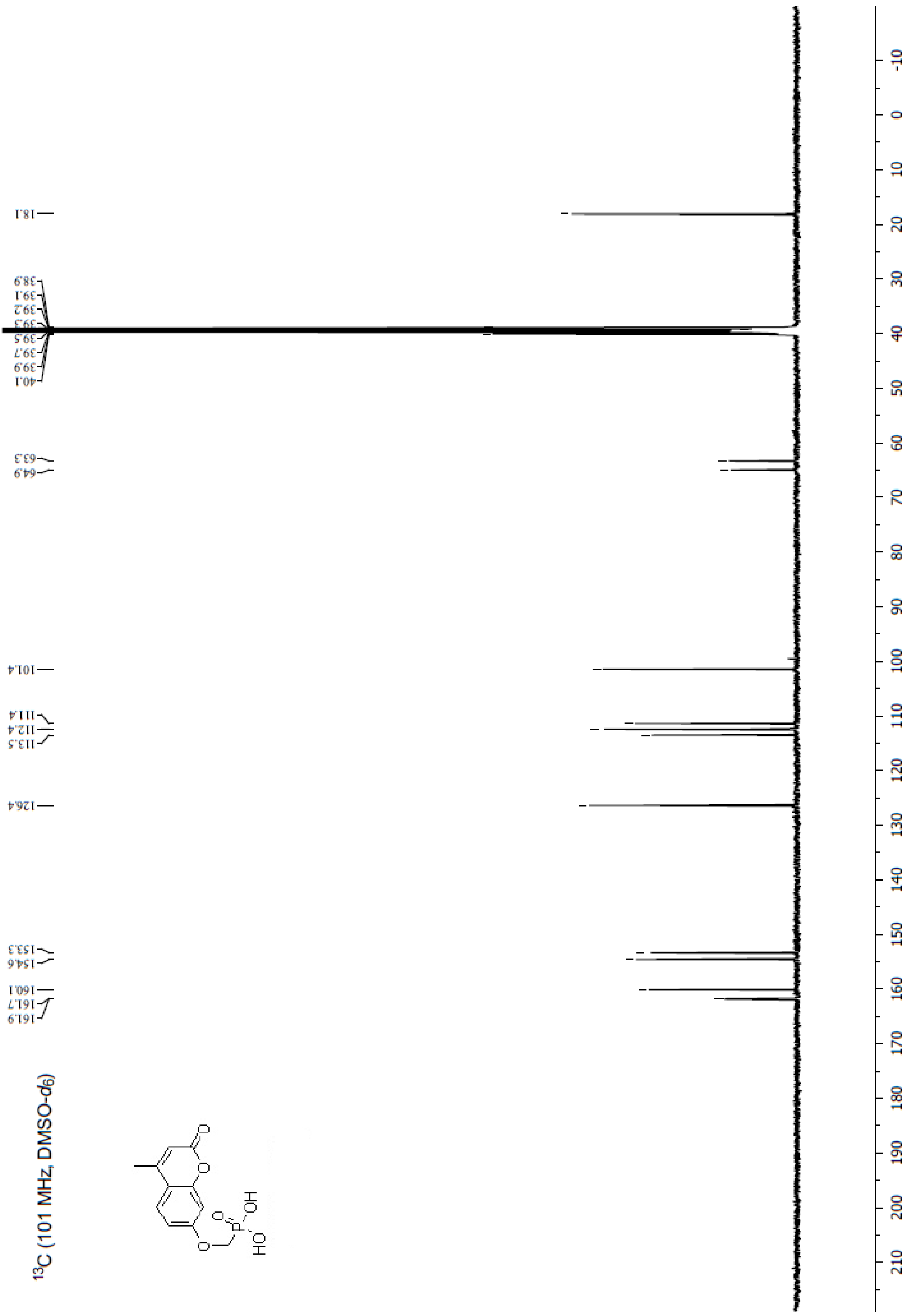
18.1
38.9
39.1
39.2
39.3
39.5
39.7
39.9
40.1
63.3
64.9
101.4
111.4
112.4
113.5
126.4
153.3
154.6
160.1
161.7
161.9
13C (101 MHz, DMSO-d6)
-10
0
10
20
30
40
50
60
70
80
90
100
110
120
130
140
150
160
170
180
190
200
210

d2b) Mass spec

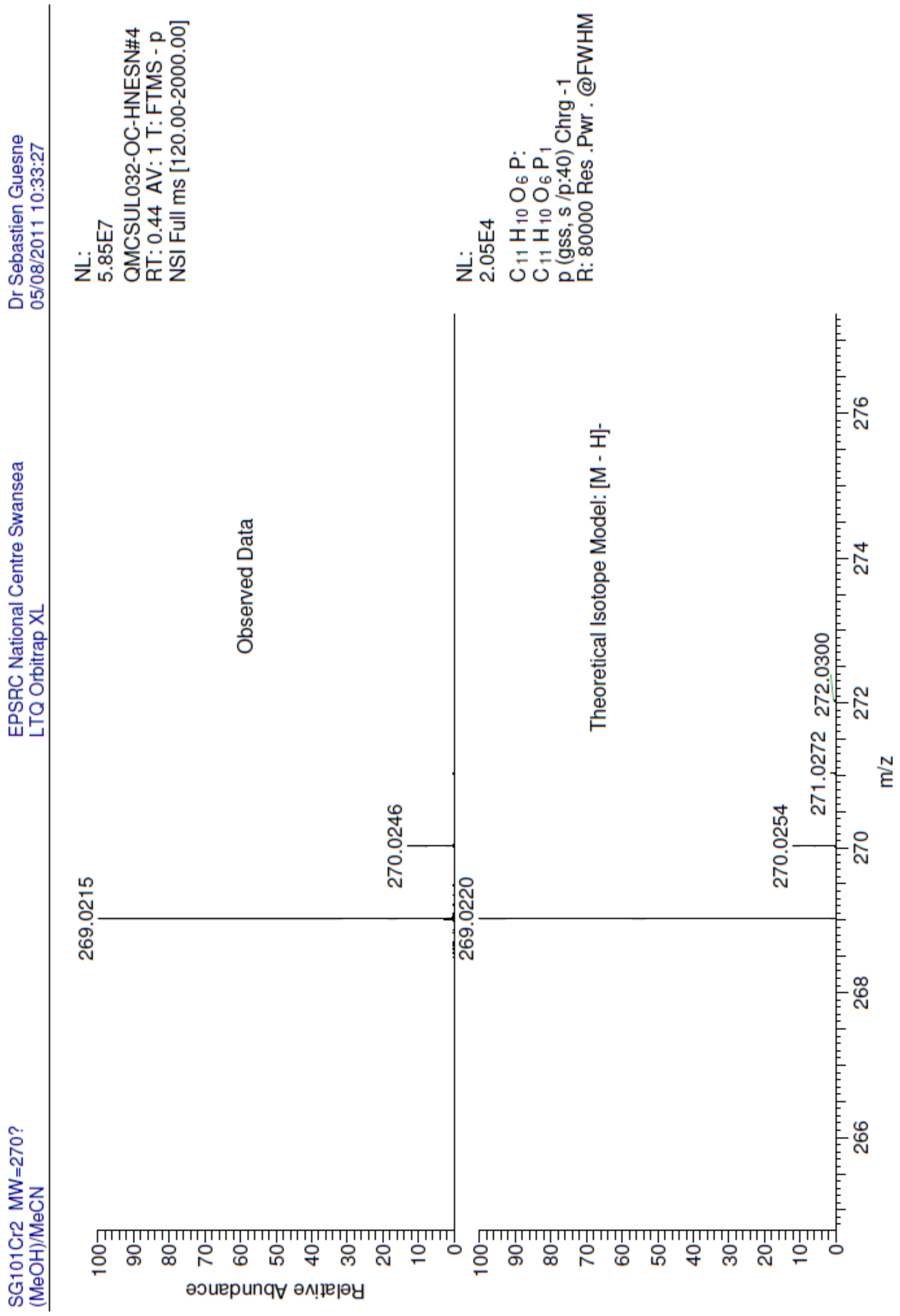

e2b) IR spectrum

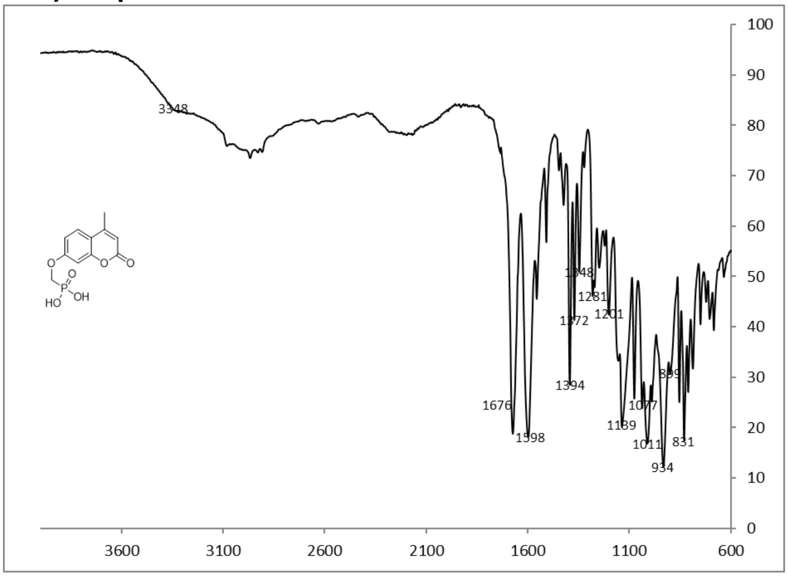

Spectra for precursor to compound 3 i) and ii) and compound 3 a3)-d3)

i) ^{1}H **NMR**

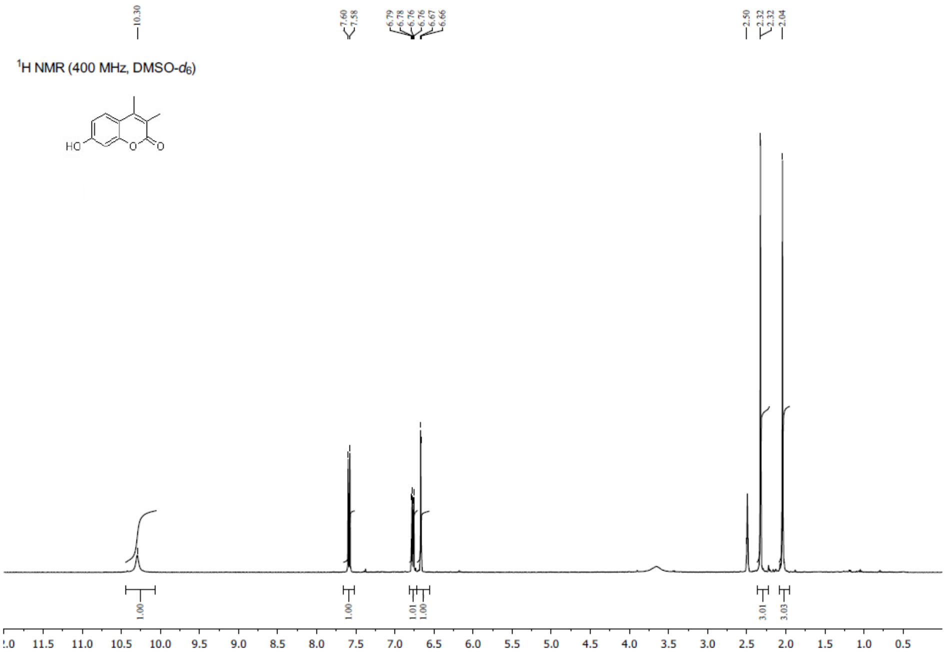

ii) ^{13}C **NMR**

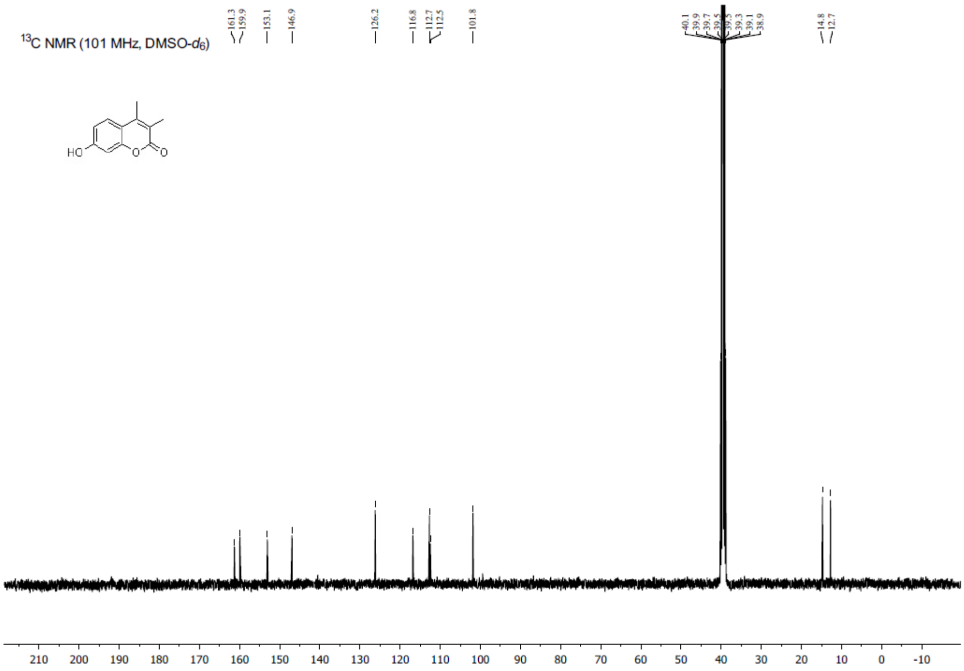

a3) ^{1}H NMR

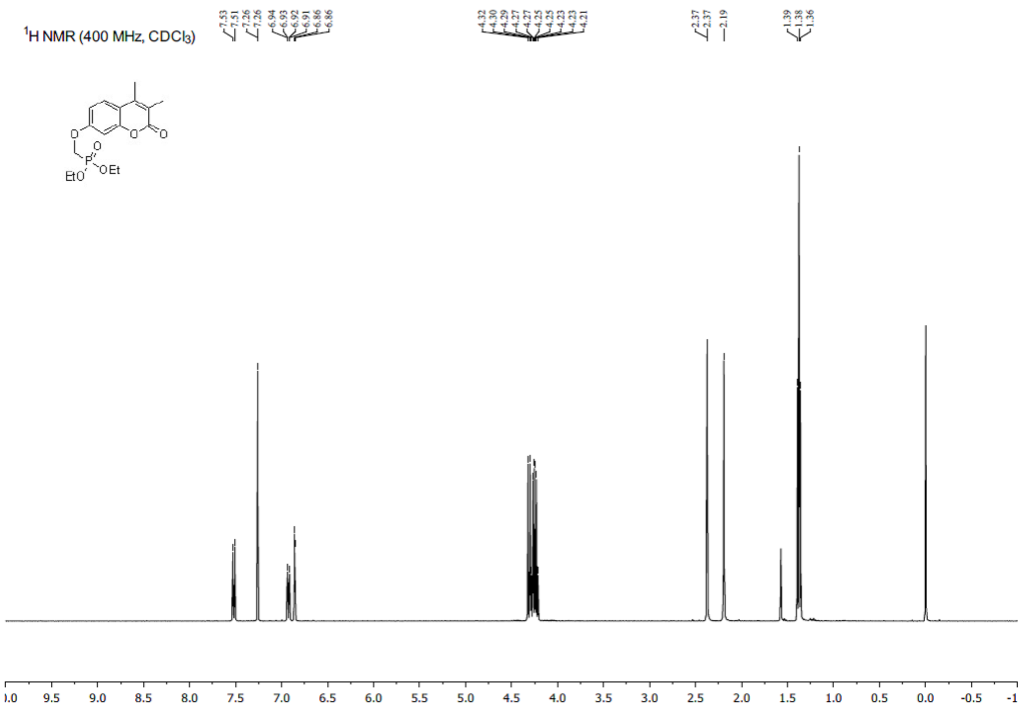

b3) ^{31}P NMR

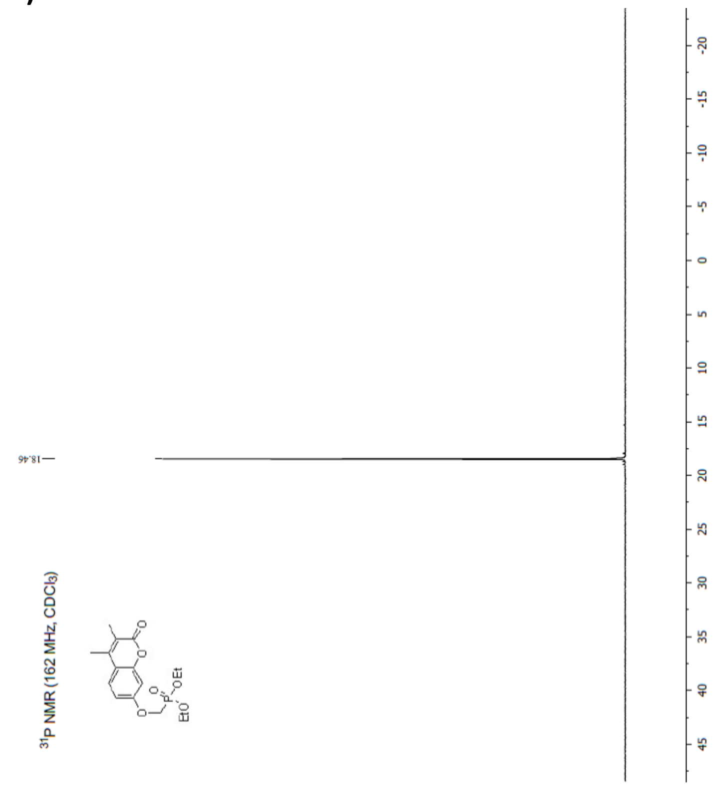

c3) ^{13}C NMR

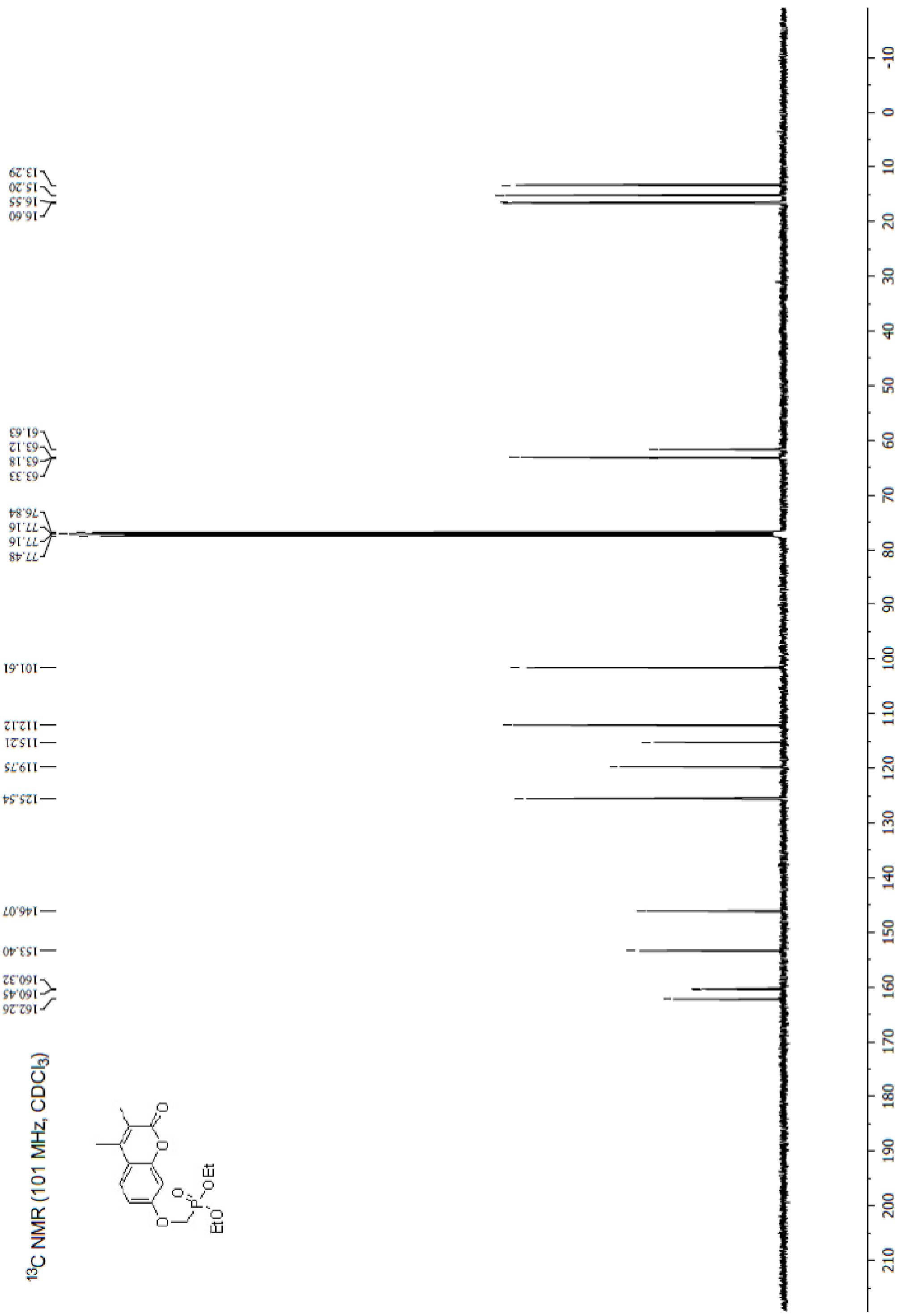

13C NMR (101 MHz, CDCl3)
13.29
15.20
16.55
16.60
61.63
63.12
63.18
63.33
76.84
77.16
77.16
77.48
101.61
112.12
115.21
119.75
125.54
146.07
153.40
160.32
160.45
162.26

d3) Mass spec

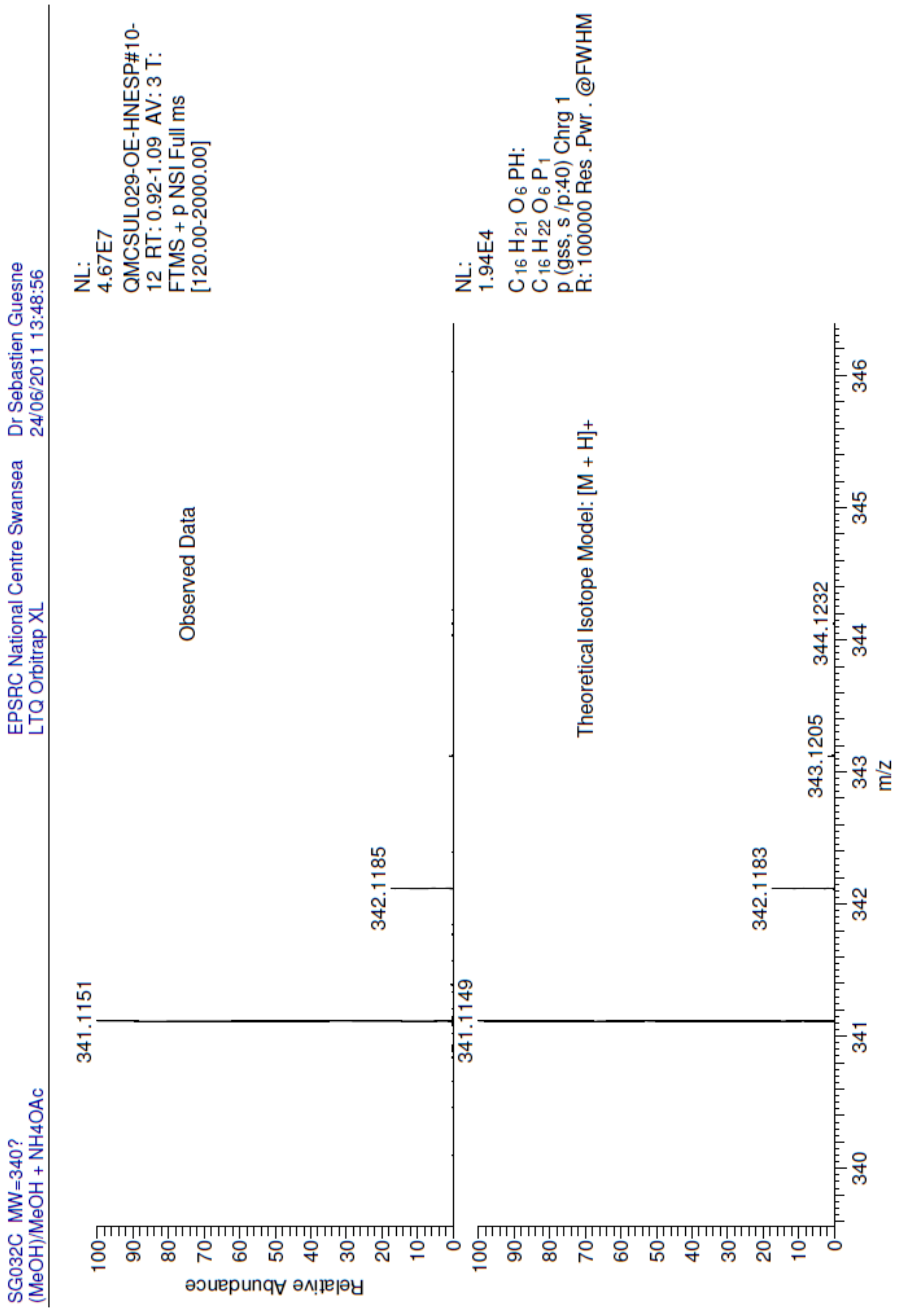
SG032C MW=340?
(MeOH)/MeOH + NH4OAc
EPSRC National Centre Swansea
LTQ Orbitrap XL
Dr Sebastien Guesne
24/06/2011 13:48:56
NL:
4.67E7
QMCSUL029-OE-HNESP#10-12 RT: 0.92-1.09 AV: 3 T: FTMS + p NSI Full ms [120.00-2000.00]
Observed Data
341.1151
342.1185
NL:
1.94E4
C 16 H 21 O 6 PH:
C 16 H 22 O 6 P 1
p (gss, s /p:40) Chrg 1
R: 100000 Res .Pwr . @FWHM
Theoretical Isotope Model: [M + H]+
341.1149
342.1183
343.1205
344.1232
Relative Abundance
m/z

e3) IR spectrum

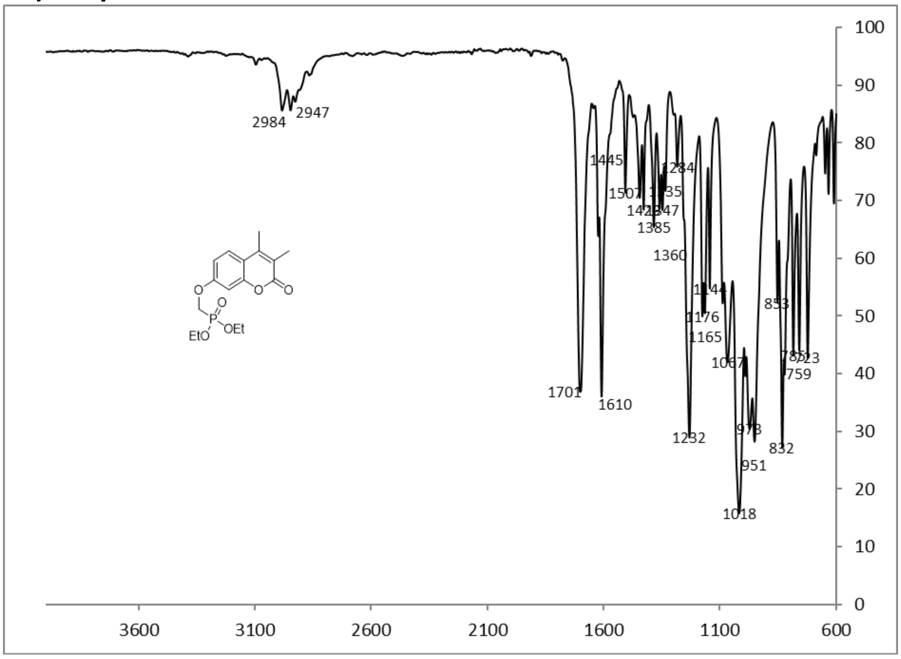

Spectra for precursor to compound 4 i) and ii) and compound 4 a4)-d4)

i) ^{1}H NMR

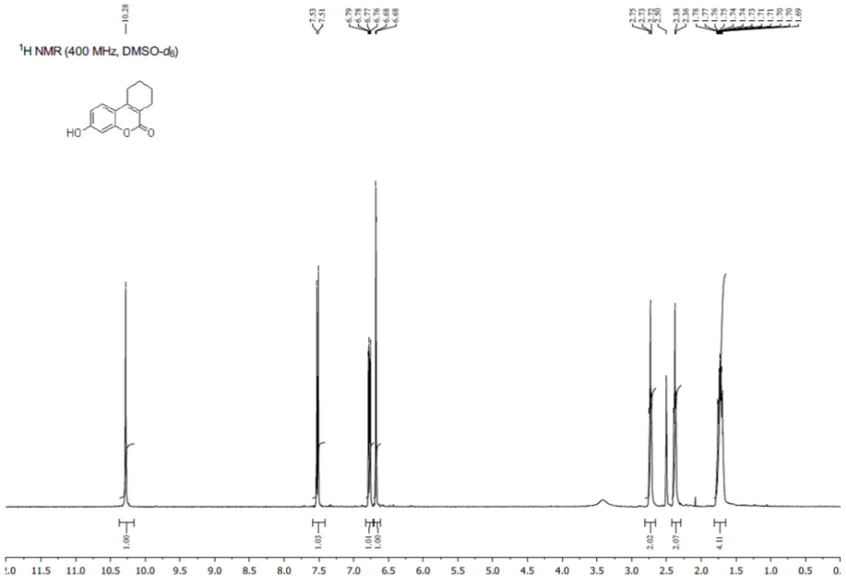

ii) 13C NMR

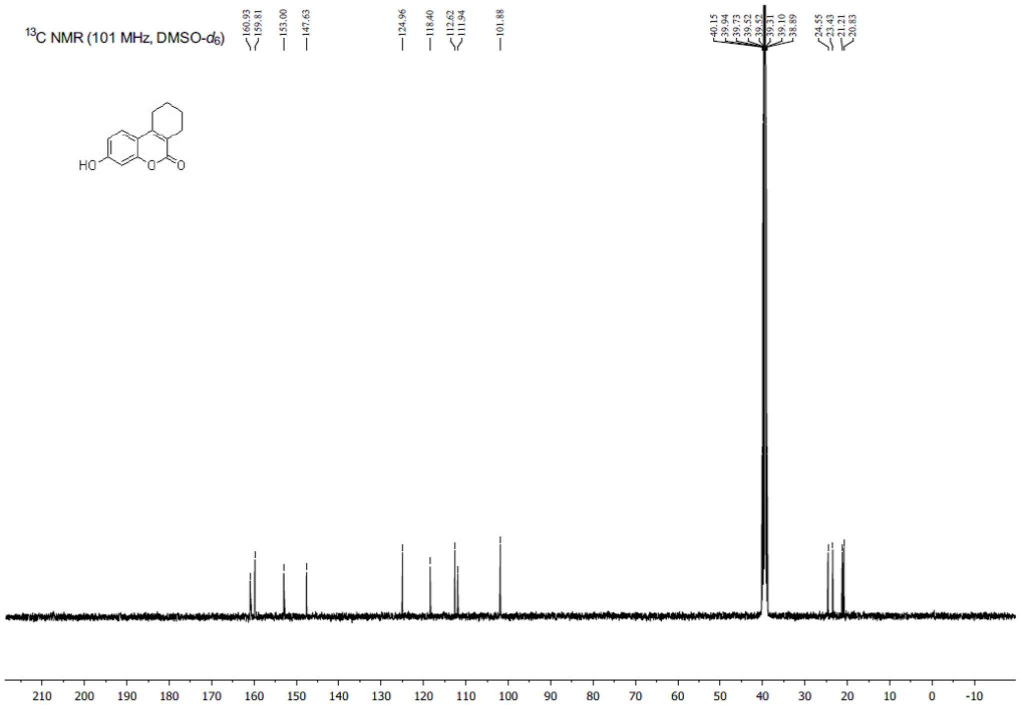

a4) ^{1}H NMR

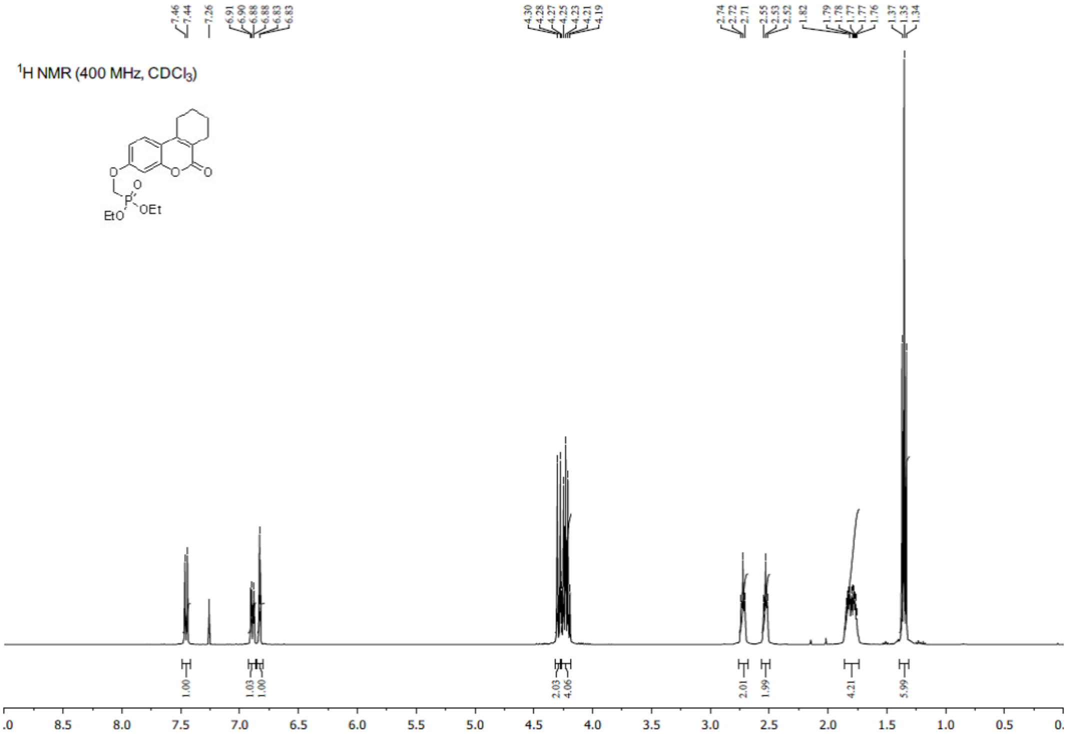

b4) ^{31}P NMR

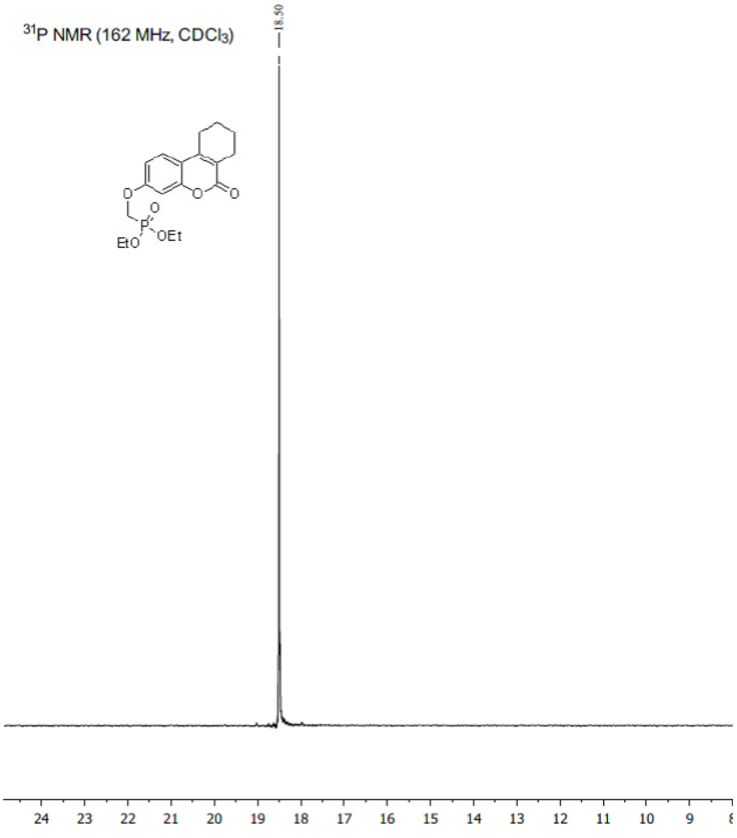

c4) ^{13}C NMR

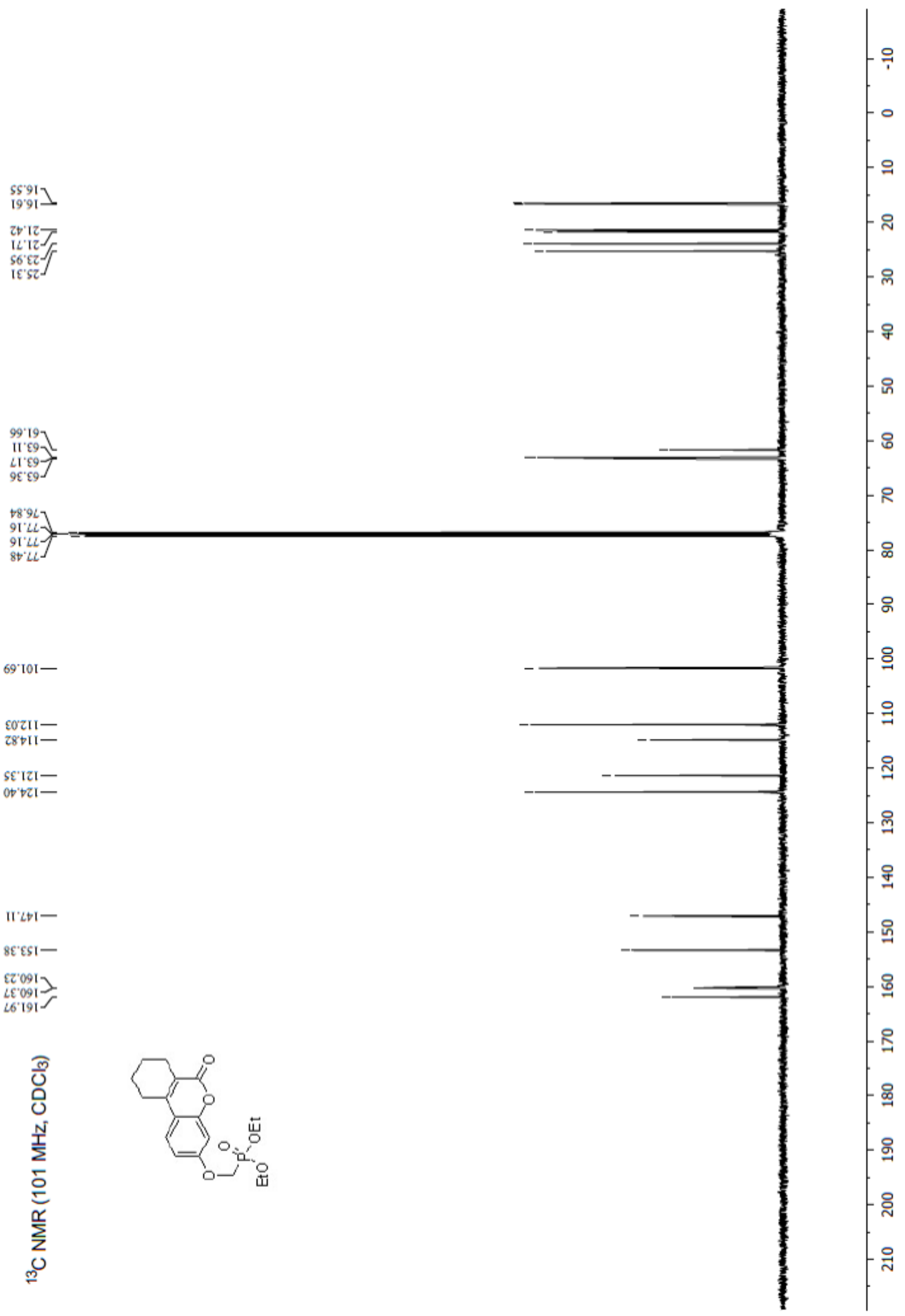
13C NMR (101 MHz, CDCl3)
16.55
16.61
21.42
21.71
23.95
25.31
61.66
63.11
63.17
63.36
76.84
77.16
77.16
77.48
101.69
112.03
114.82
121.35
124.40
147.11
153.38
160.23
160.37
161.97
EtO
OEt
-10
0
10
20
30
40
50
60
70
80
90
100
110
120
130
140
150
160
170
180
190
200
210

d4) mass spec

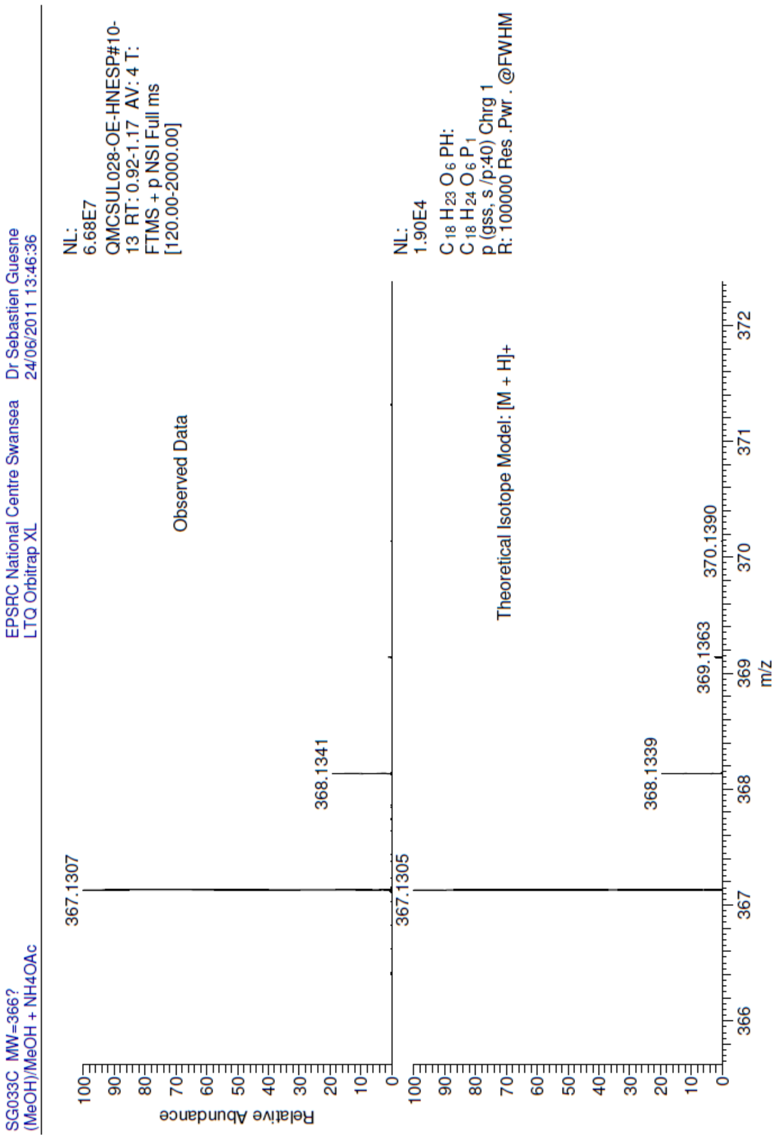
SG033C MW=366?
(MeOH)/MeOH + NH4OAc
EPSRC National Centre Swansea
LTQ Orbitrap XL
Dr Sebastien Guesne
24/06/2011 13:46:36
NL:
6.68E7
QMCSUL028-OE-HNESP#10-13 RT: 0.92-1.17 AV: 4 T: FTMS + p NSI Full ms [120.00-2000.00]
Observed Data
367.1307
368.1341
NL:
1.90E4
C 18 H 23 O 6 PH:
C 18 H 24 O 6 P 1
p (gss, s /p:40) Chrg 1
R: 100000 Res .Pwr . @FWHM
Theoretical Isotope Model: [M + H]+
367.1305
368.1339
369.1363
370.1390
Relative Abundance
m/z

e4) IR spectrum

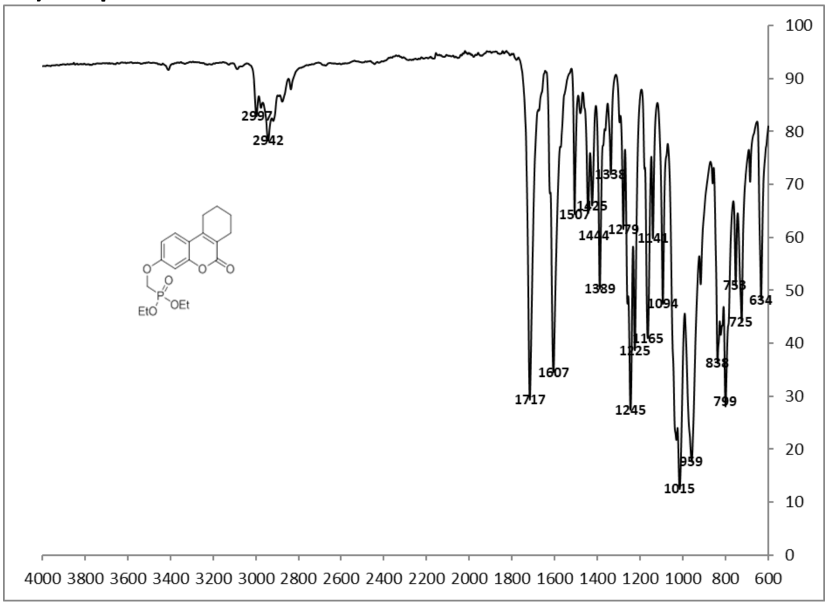

Spectra for compound 5

a5)^{1}H NMR

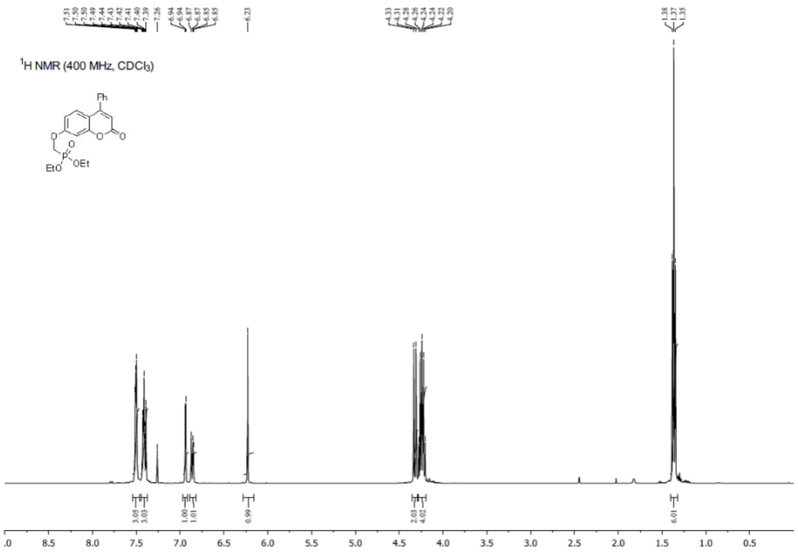

b5) ^{31}P NMR

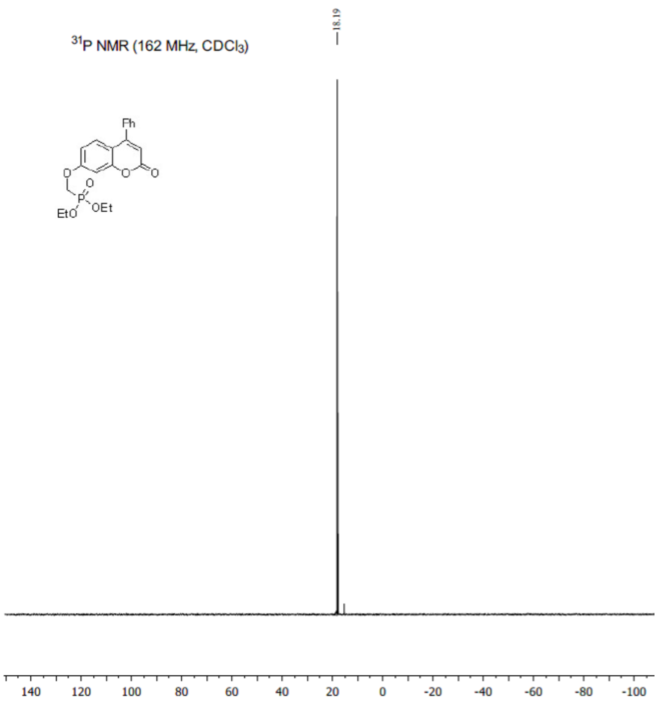

c5) ^{13}C NMR

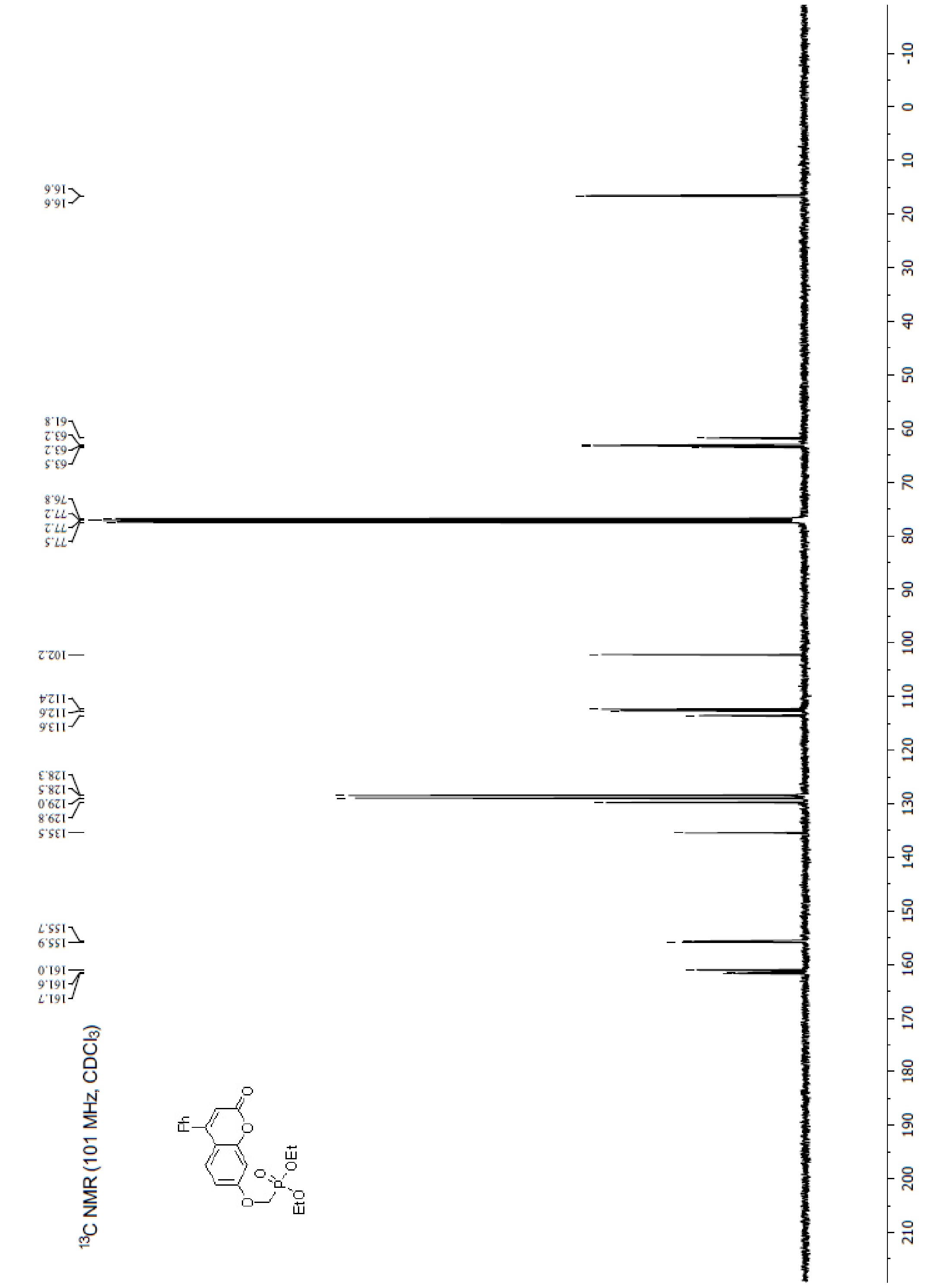

13C NMR (101 MHz, CDCl3)
16.6
16.6
61.8
63.2
63.2
63.5
76.8
77.2
77.2
77.5
102.2
112.4
112.6
113.6
128.3
128.5
129.0
129.8
135.5
155.7
155.9
161.0
161.6
161.7
Ph
O
OEt
EtO
P

d5) Mass spec

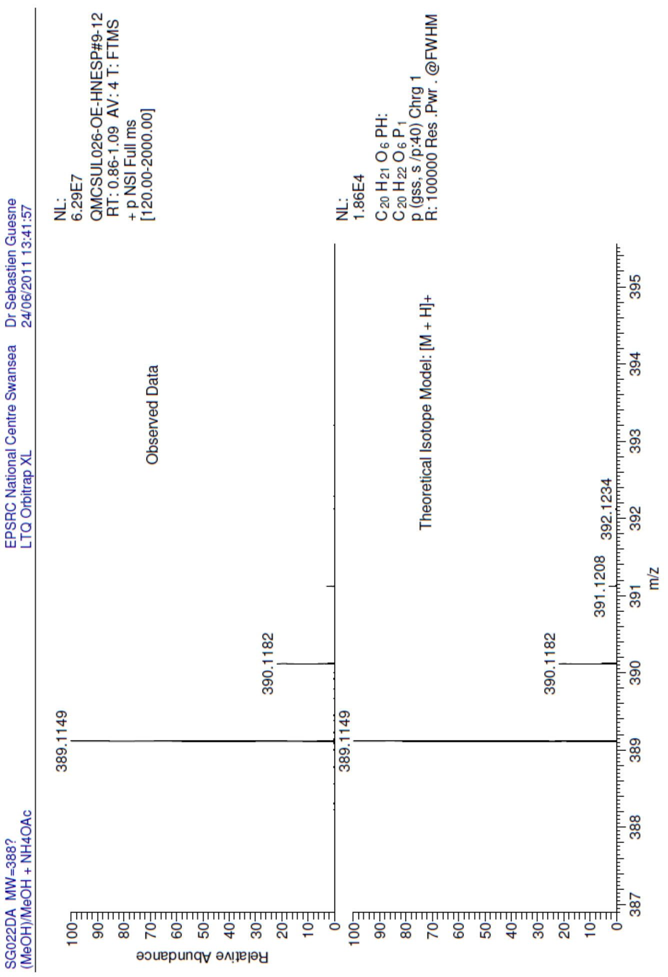

e5) IR spectrum

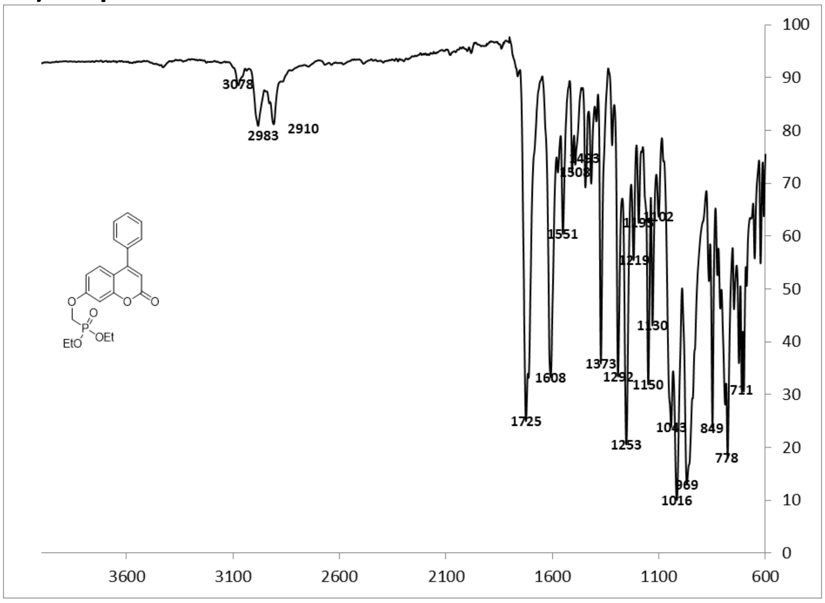

Spectra for compound 6

a6) ^{1}H NMR

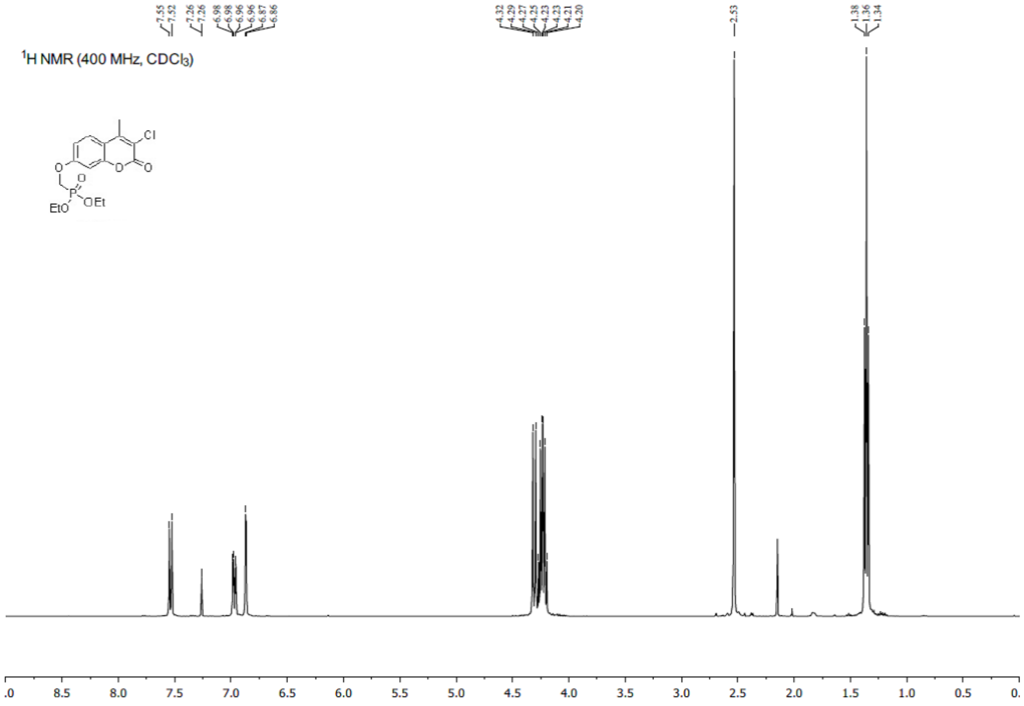

b6) ^{31}P NMR

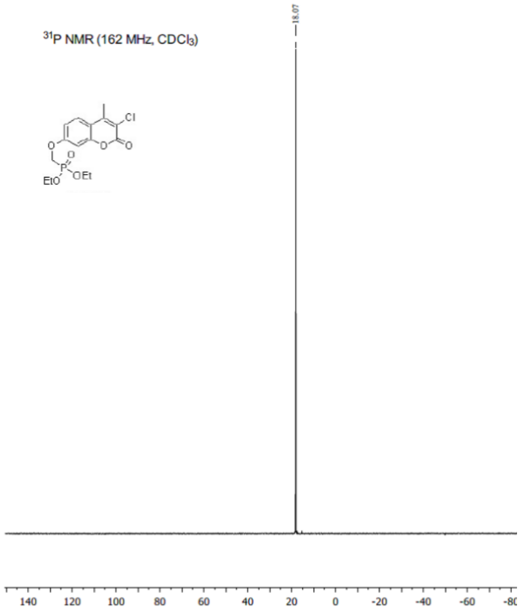

c6) ^{13}C NMR

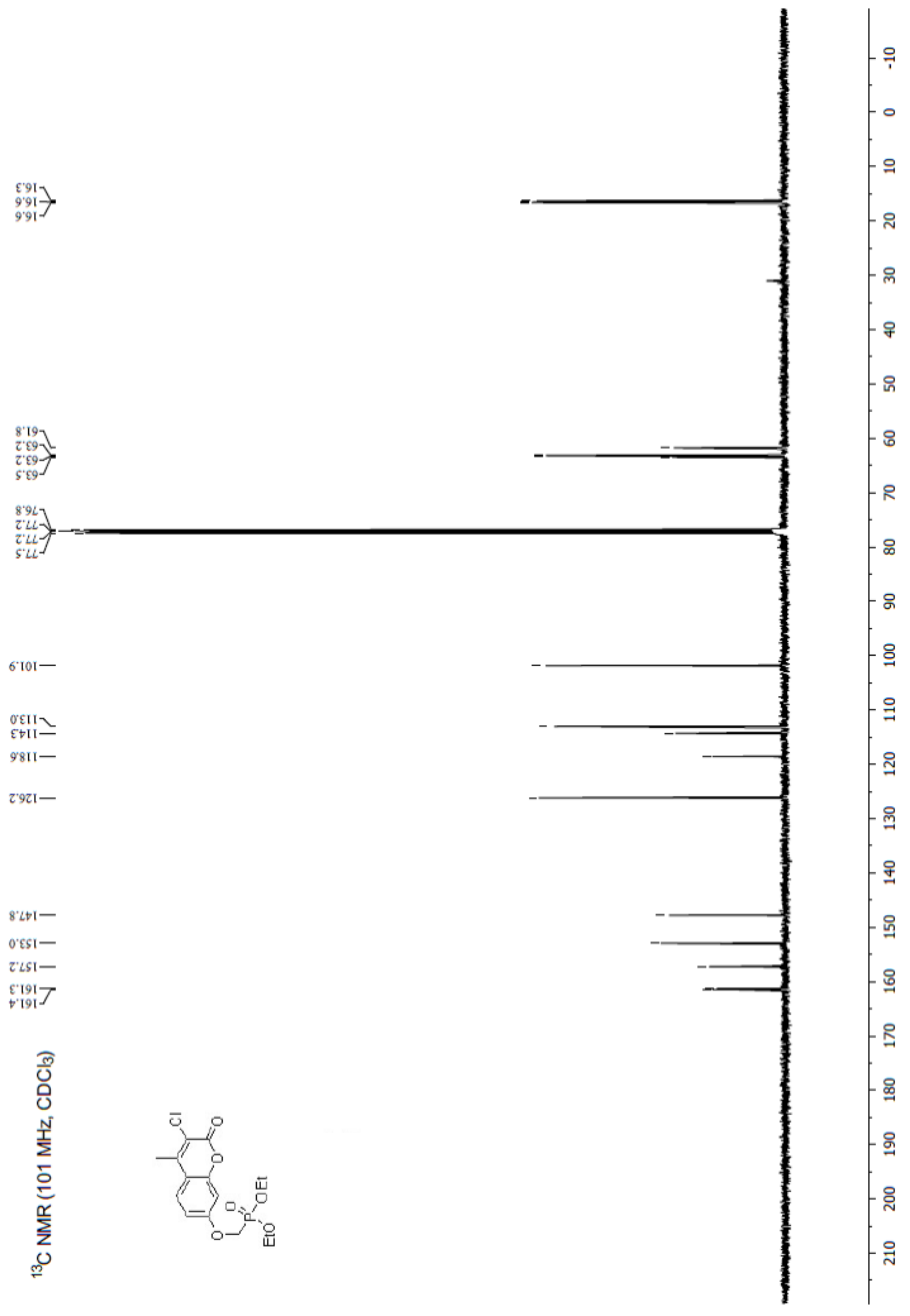

13C NMR (101 MHz, CDCl3)
16.3
16.6
16.6
61.8
63.2
63.2
63.5
76.8
77.2
77.2
77.5
101.9
113.0
114.3
118.6
126.2
147.8
153.0
157.2
161.3
161.4
Cl
O
O
O
P
EtO
OEt

d6) Mass spec

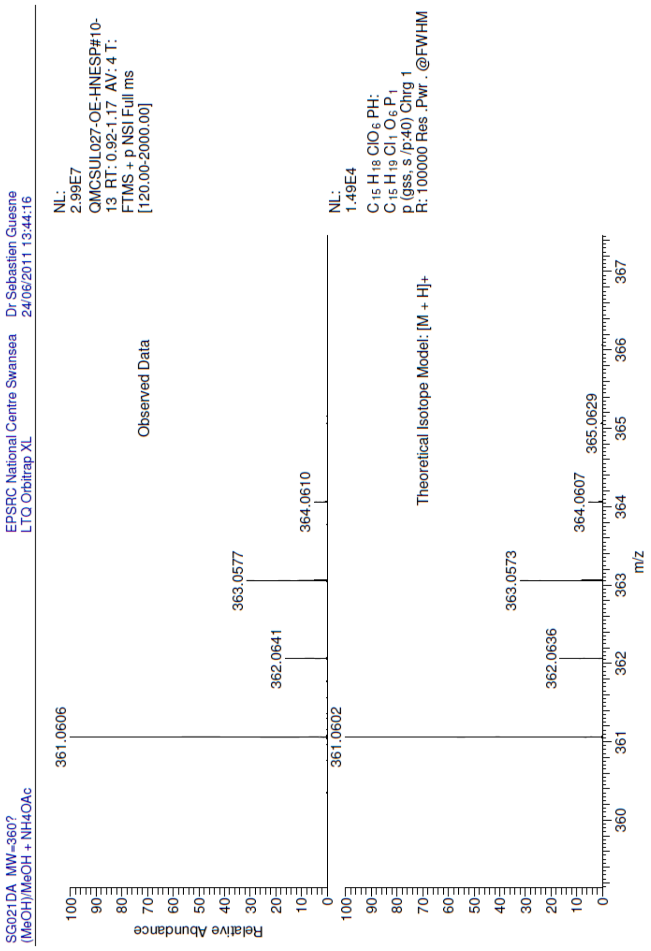

e6) IR spectrum

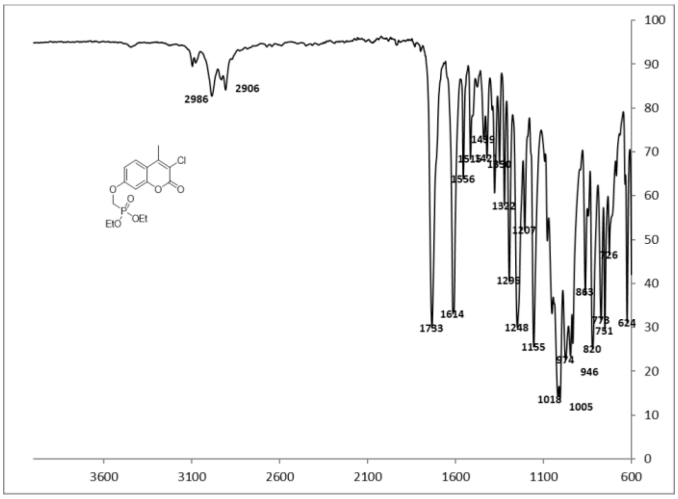

Spectra for precursor to compound 7 i) and compound 7 a7)-d7)

i) ^{1}H NMR

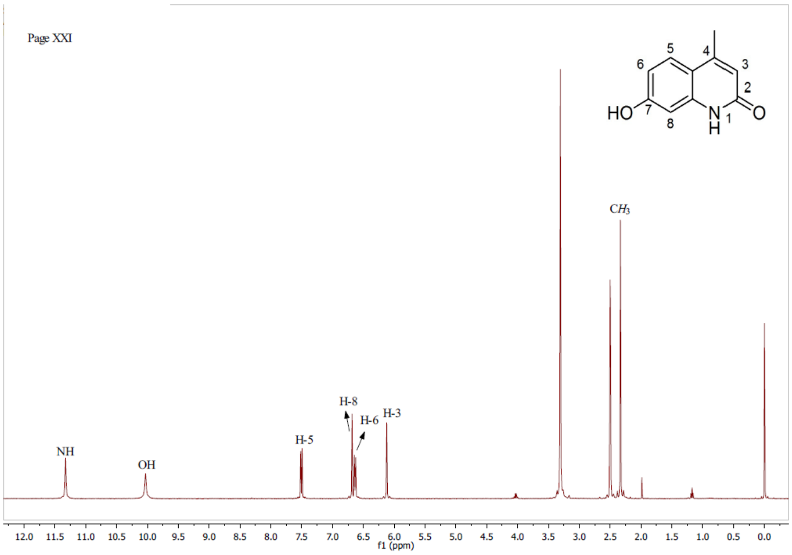

a7) ^{1}H NMR

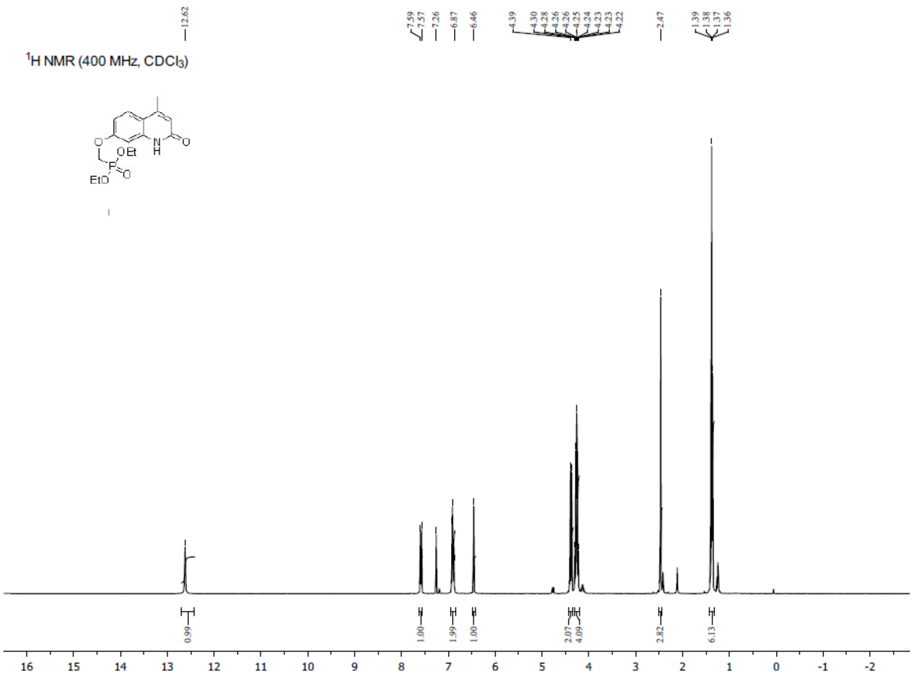

b7) ^{31}P NMR

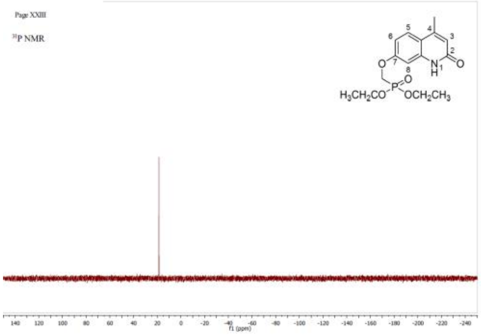

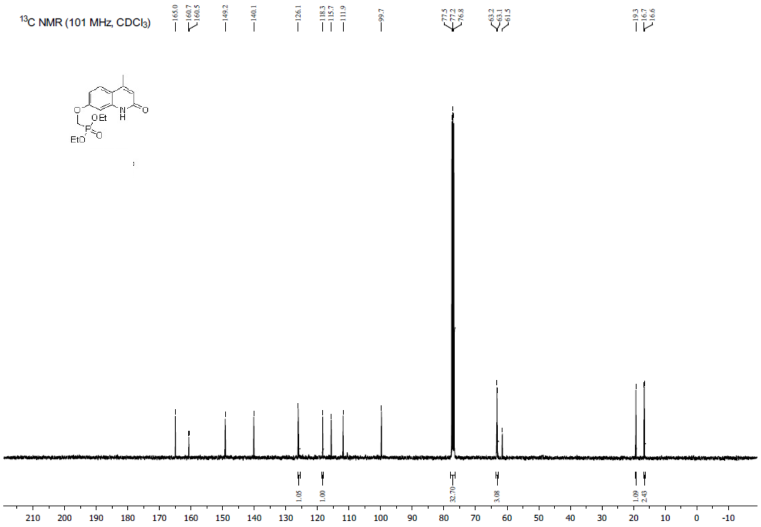

c7) ^{13}C NMR and DEPT 135

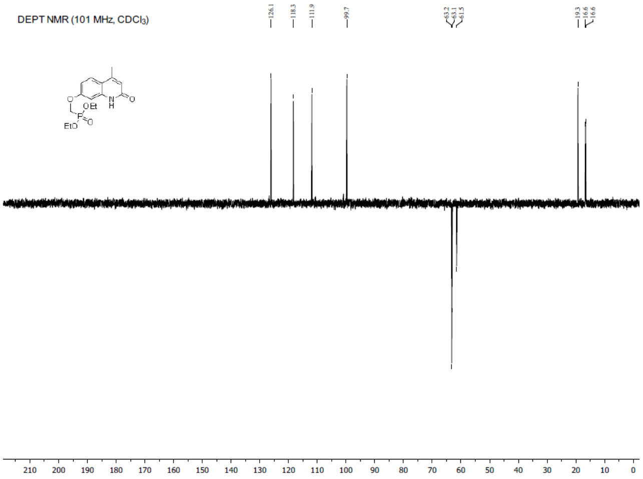

COSY spectrum of Compound 7 showing coupling interaction between protons

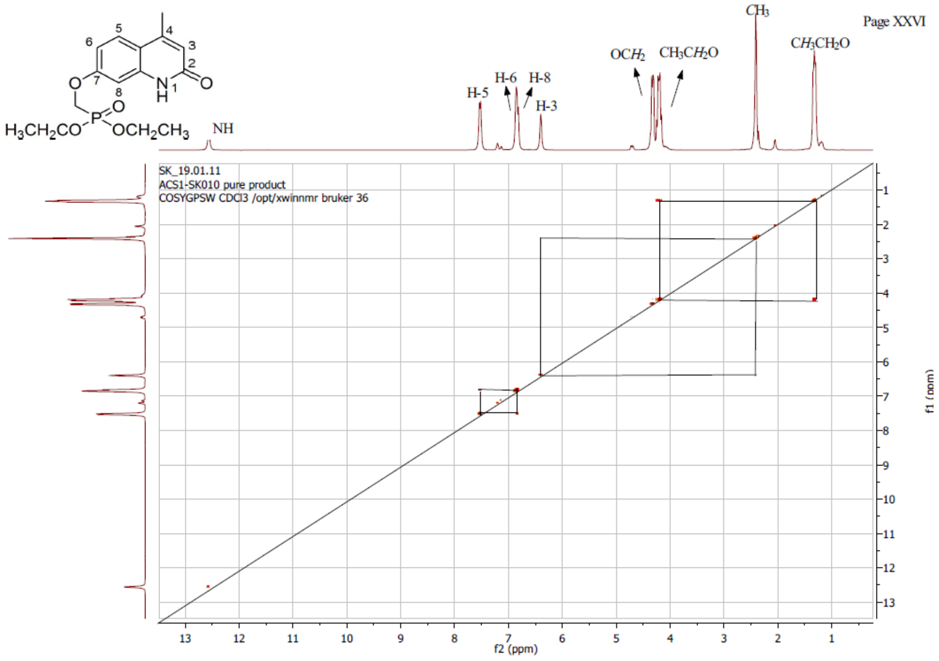

:

HSQC spectrum of compound 7 showing proton and carbon connectivity.

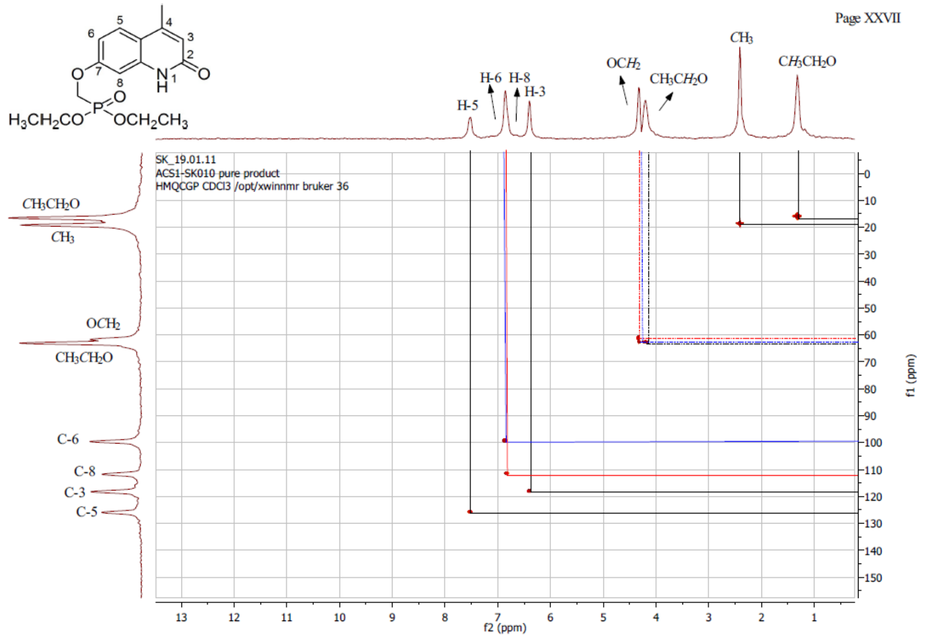

e7) IR spectrum

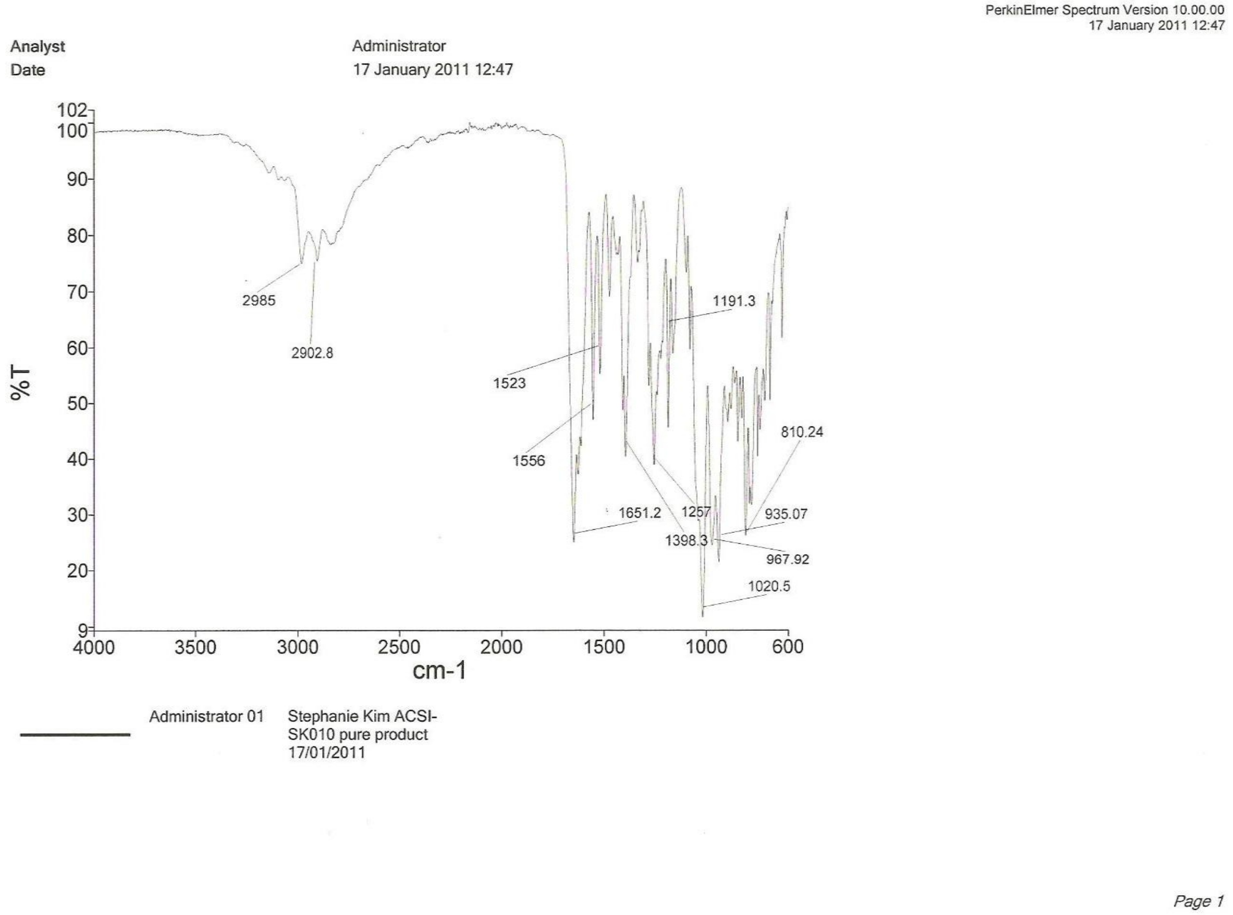

f7 Mass spectrum

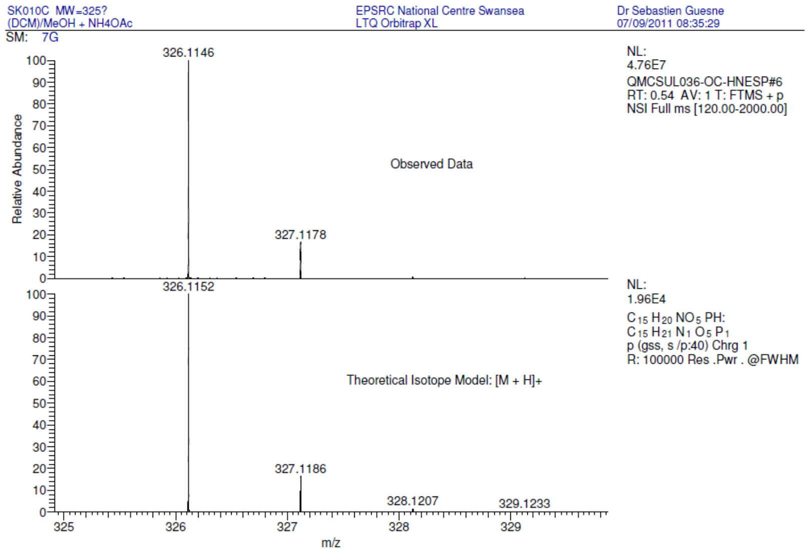

Spectra for precursor to compound compound 8

a8) ^{1}H NMR

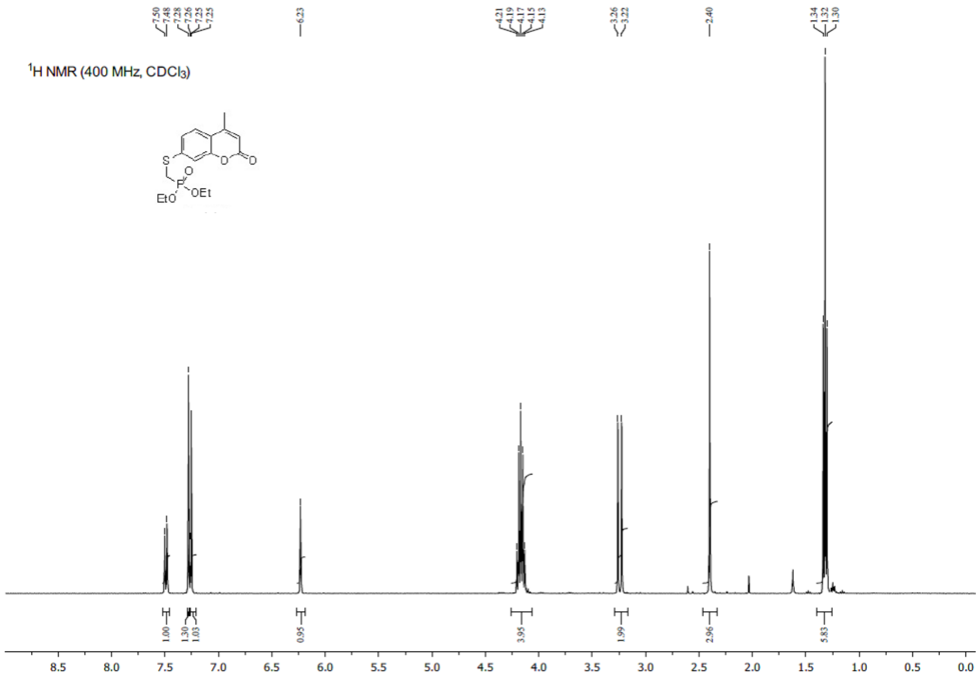

b8) ^{31}P NMR

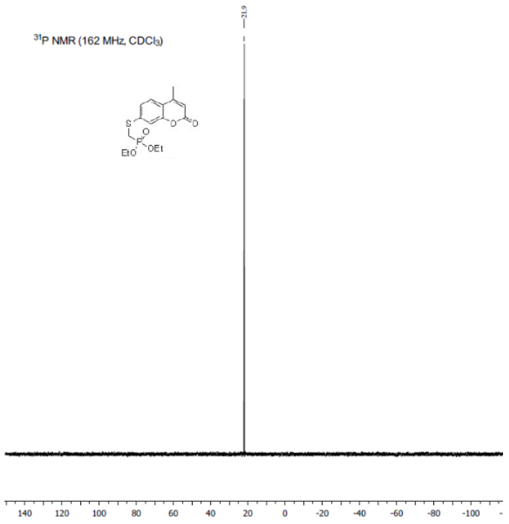

c8) ^{13}C NMR

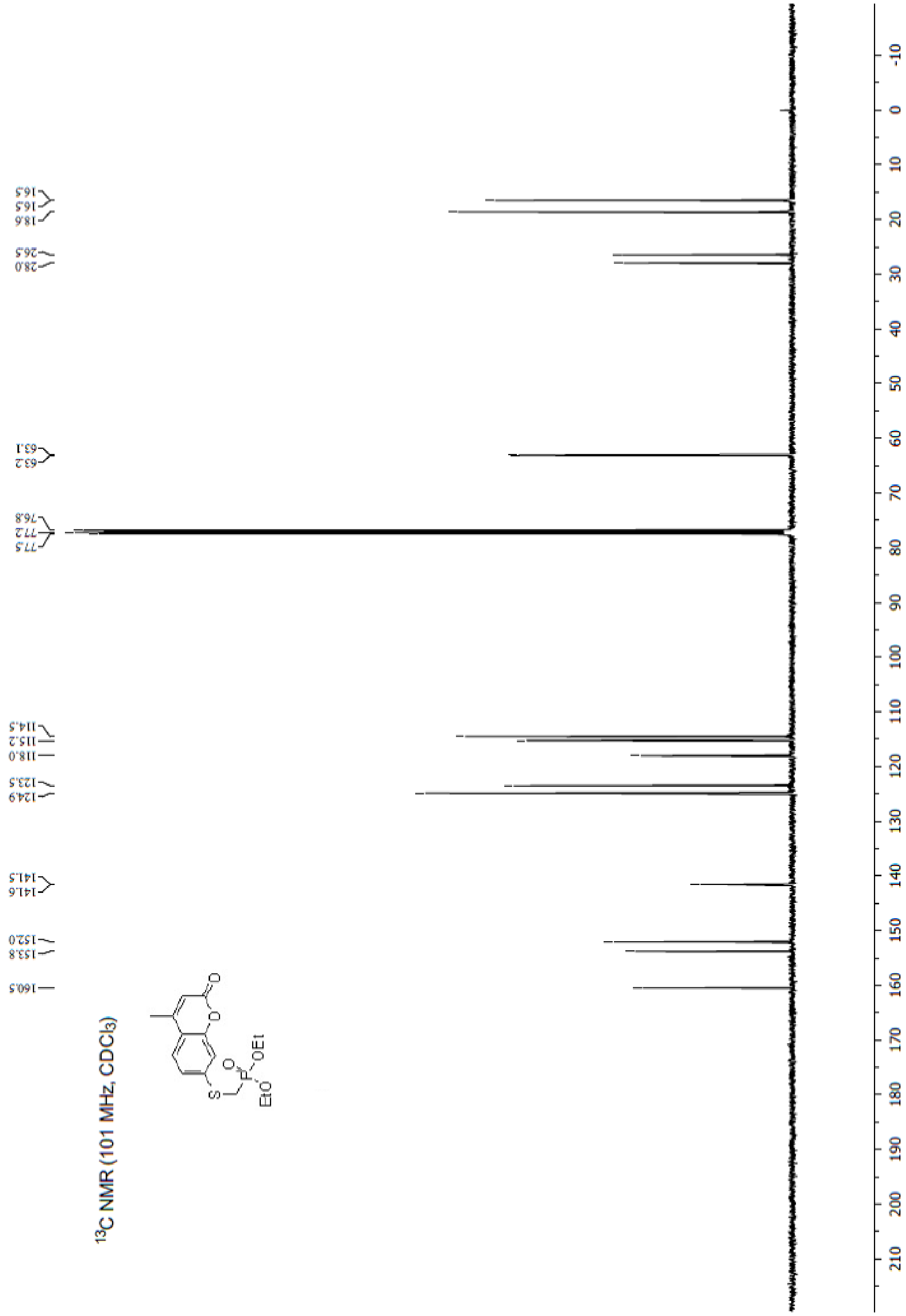
13C NMR (101 MHz, CDCl3)
16.5
16.5
18.6
26.5
28.0
63.1
63.2
76.8
77.2
77.5
114.5
115.2
118.0
123.5
124.9
141.5
141.6
152.0
153.8
160.5
EtO
OEt
S
O
P

d8) Mass spec

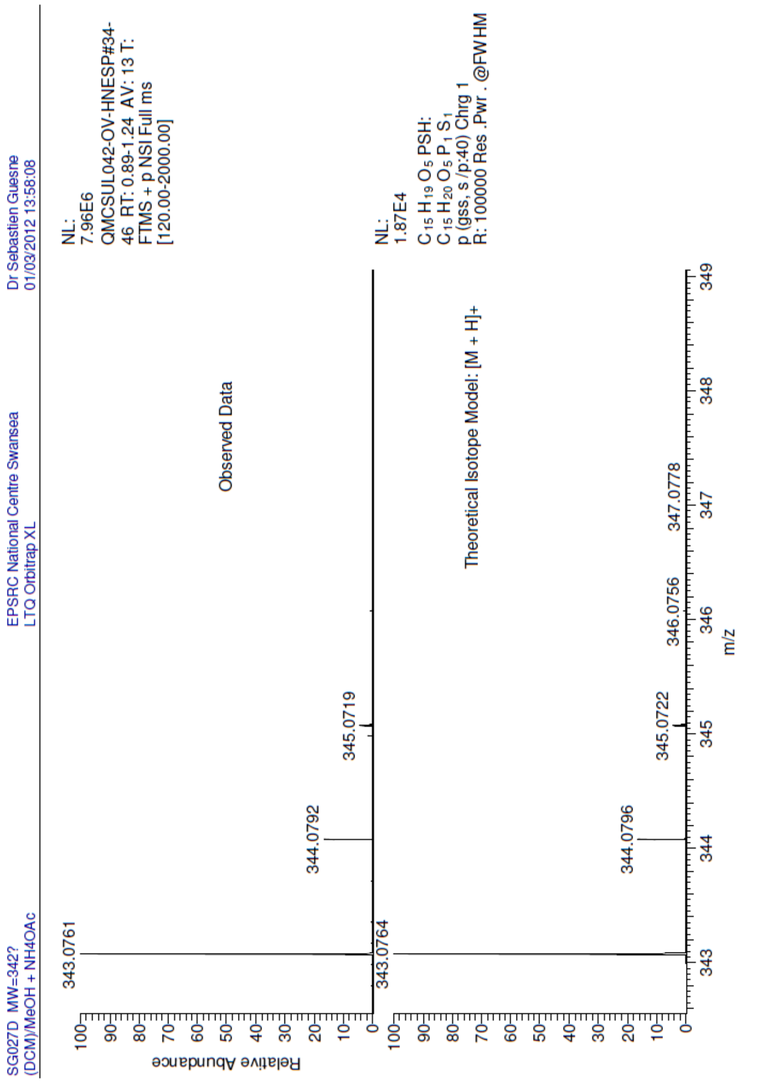

e8) IR spectrum

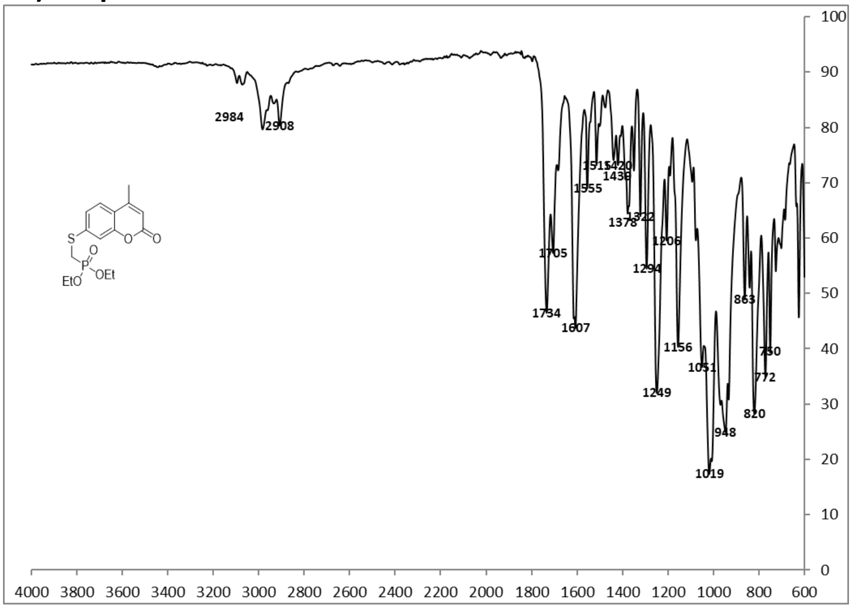

Crystallographic data

The intensity data for **1** were collected on an EnrafNonius CAD-4 diffractometer using Mo-Kα radiation(λ 0.71069 Å) with an ω-2θ scan at 160 K. The unit cell parameters were determined by least-squares refinement on diffractometer angles 9.96 ≤θ≤ 13.62° for 25 automatically centred reflections. [1] All data were corrected for absorption by semi-empirical methods (ψ scan) [2] and for Lorentz-polarization effects by XCAD4. [3] The structure was solved by direct method using SHELXS-97 [4] and refined anisotropically (non-hydrogen atoms) by full-matrix least-squares on F2 using the SHELXL-97 and SHELXL-2013 program.[4] The H atoms were calculated geometrically and refined with a riding model. The disorder affecting C(12) and C(13) was modelled and refined using SHELXL-2013. The program ORTEP-3[5] was used for drawing the molecules. WINGX[6] used to prepare material for publication.

The intensity data for **2** were collected at 120 K using a Nonius Kappa CCD area detector diffractometer mounted at the window of a molybdenum rotating anode (50 KV, 85 mA, λ=0.71073 Å). The crystal-to-detector distance was 30 mm and ϕ and Ω scans (1.0° increments, 20 s exposure time) were carried out to fill the Ewald sphere. Data collection and processing were carried out using DirAx, [7] COLLECT, [8] DENZO[9] and an empirical absorption correction was applied using SADABS. [10] The structures was solved by the heavy-atom method using the DIRDIF99 program, [11] and refined anisotropically (non-hydrogen atoms) by full-matrix least-squares on F2 using the SHELXL-97 and SHELXL-2013 programs. [4] The disorder affecting C(12) and C(13) was modelled and refined using SHELXL-2013. The programs ORTEP-3,[5] and PLATON,[12] were used for drawing the molecules. WINGX60 was used to prepare material for publication.

The intensity data for compound **2a** were measured on a On KAPPA-APEXII-DUO, APEX2 (Bruker, 2010.) A total of 1748 frames were collected. The total exposure time was 4.86 h. The frames were integrated with the Bruker SAINT software package[13] using a narrow-frame algorithm. Data were corrected for absorption effects using the multi-scan method (SADABS).[10] The structure was solved and refined using the Bruker SHELXTL Software Package and SHELXL-2018,[4] molecular graphics and software used to prepare material for publication: SHELXTL and PLATON (Spek, 2009).[5,12]

Identification code	Compound 1	Compound 2	Compound 2a
CCDC	892006	892005	892004
Empirical formula	$C_{15} H_{19} O_6 P$	$C_{13} H_{15} O_6 P$	$C_{13}H_{20}Na_3O_{10}P$
Formula weight	326.27	298.22	436.23
Temperature	160(2) K	120(2) K	100(2) K
Wavelength	0.71073 Å	0.71073 Å	0.71073 Å
	a =16.6014(10) Å	a = 7.0955(3) Å	a = 14.7973(8) Å
	b = 8.812(2) Å	b = 24.2238(10) Å	b = 6.4554(3) Å
	c = 11.132(2) Å	c = 8.2176(4) Å	c = 20.7665(11) Å
	α = 90°.	α = 90°.	α = 90°
	β = 105.12(2)°.	β = 106.643(2)°.	β = 106.7280(10)°
	γ = 90°.	γ = 90°.	γ = 90°
Space group	$P2_1/n$	$P2_1/c$	$P2_1/n$
Volume	1572.2(5) Å^3	1353.27(10) Å^3	1899.72(17)Å^3
Z	4	4	4
Density (calculated)	1.378 Mg/m^3	1.464 Mg/m^3	1.525 Mg/cm^3
Absorption coefficient	0.201 mm^{-1}	0.226 mm^{-1}	0.262 mm^{-1}
F(000)	688	624	904
Crystal size	0.4 x 0.2 x 0.2 mm^3	0.07 x 0.05 x 0.03 mm^3	0.20 x 0.24 x 0.48 mm^3
Theta range for data collection	1.987 to 27.469°.	2.996 to 26.727°.	1.51 to 27.52°
Index ranges	-1<=h<=21, 0<=k<=11, -14<=l<=13	-8<=h<=8, -30<=k<=30, -10<=l<=10	-19<=h<=19, -8<=k<=8, -26<=l<=26
Reflections collected	3800	10427	24285
Independent reflections	3585 [R(int) = 0.0130]	2846 [R(int) = 0.0601]	4355 [R(int) = 0.0448]
Completeness to theta	= 25.242° 99.1 %	= 25.242 99.1 %	99.5%
Refinement method	Full-matrix least-squares on F^2	Full-matrix least-squares on F^2	Full-matrix least-squares on F^2
Data / restraints / parameters	3585 / 38 / 221	2846 / 38 / 206	4355 / 0 / 312
Goodness-of-fit on F^2	1.051	0.949	1.049
Final R indices [I>2sigma(I)]	R1 = 0.0697, wR2 = 0.1703	R1 = 0.0705, wR2 = 0.1576	R1 = 0.0328, wR2 = 0.0754
R indices (all data)	R1 = 0.1878, wR2 = 0.2212	R1 = 0.1092, wR2 = 0.1864	R1 = 0.0490, wR2 = 0.0828
Largest diff. peak and hole	0.664 and -0.912 e.Å^{-3}	0.381 and -0.380 e.Å^{-3}	0.402 and -0.256 eÅ^{-3}

Bond lengths (Å)	Compound **1**	Compound **2**
C(1)-O(2)	1.209(5)	1.213(5)
C(1)-O(1)	1.373(6)	1.380(4)
C(1)-C(2)	1.451(7)	1.439(5)
C(2)-C(3)	1.340(6)	1.345(5)
C(2)-H(2)	0.9500	0.9500
C(5)-O(1)	1.387(5)	1.380(4)
C(9)-O(3)	1.357(6)	1.370(5)
C(11)-O(3)	1.421(4)	1.433(5)
C(12A)-O(5)	1.423(18)	-
C(12B)-O(5)	1.453(19)	-
C(12A)-O(6)	-	1.489(10)
C(12B)-O(6)	-	1.431(16)

C(12A)-C(13A)	1.46(4)	1.480(13)
C(12B)-C(13B)	1.50(3)	1.40(3)
C(11)-P(1)	1.802(5)	1.794(4)
O(4)-P(1)	1.469(3)	1.552(3)
O(5)-P(1)	1.563(4)	1.479(3)
O(6)-P(1)	1.563(4)	1.560(3)
O(4)-H(4)	-	0.95(6)

Bond angles (˚)	Compound **1**	Compound **2**
O(5)-P(1)-O(4)	115.4(2)	112.49(17)
O(5)-P(1)-O(6)	104.0(2)	114.82(19)
O(4)-P(1)-O(6)	116.5(2)	106.62(16)
O(5)-P(1)-C(11)	107.3(2)	112.31(18)
O(4)-P(1)-C(11)	111.2(2)	108.17(18)
O(6)-P(1)-C(11)	101.1(2)	101.67(17)
P(1)-O(4)-H(4)		110(3)
C(12A)-O(5)-P(1)	123.6(9)	
C(12B)-O(5)-P(1)	121.0(7)	
C(12A)-O(6)-P(1)		126.4(4)
C(12B)-O(6)-P(1)		116.7(8)
C(14)-O(6)-P(1)	120.3(3)	

Hydrogen bond data for 2 (Å and °).[a]

D-H...A	d(D-H)	d(H...A)	d(D...A)	<(DHA)
O(4)-H(4)..O(5)#1	0.95(6)	1.57(6)	2.510(4)	171(6)

[a] Symmetry transformations used to generate equivalent atoms: #1 x,-y+1/2,z-1/2.

Selected bond lengths and distances for compound 2a.

Bond lengths (Å) compound 2a			
C1-O1	1.3141(19)	C5-O2	1.3855(18)
C8-O3	1.441(2)	C10-O10	1.251(2)
C10-O7#1	1.2702(19)		
Na1-O5	2.2943(12)	Na1-O10	2.3058(13)
Na1-O8	2.3148(13)	Na1-O7	2.4004(13)
Na1-O9	2.2781(14)	Na2-O7	2.3092(12)
Na2-O2	2.4361(13)	Na2-O5	2.4543(13)
Na2-O3#2	2.5193(13)	Na2-O4#2	2.8348(15)
Na2-O6	2.3501(14)	Na3-O4	2.3578(13)
Na3-O9	2.3717(14)	Na3-O8#3	2.3887(14)
Na3-O7#1	2.4550(13)	Na3-O6#1	2.5694(15)
Na3-O10	2.8893(13)	O3-P1	1.6099(12)
O4-P1	1.4880(12)	O5-P1	1.4912(12)
**Bond angles (˚) compound 2a**			
O9-Na1-O5	104.78(5)	O9-Na1-O10	84.97(5)
O5-Na1-O10	92.82(5)	O9-Na1-O8	98.62(5)
O5-Na1-O8	154.99(5)	O10-Na1-O8	80.44(5)
O9-Na1-O7	95.94(5)	O5-Na1-O7	87.35(4)
O10-Na1-O7	178.99(5)	O8-Na1-O7	99.00(5)
O7-Na2-O6	89.30(5)		
O7-Na2-O2	156.84(5)	O6-Na2-O2	105.12(5)
O7-Na2-O5	85.75(4)	O6-Na2-O5	115.77(5)
O2-Na2-O5	71.74(4)	O4-Na3-O9	98.25(5)
O4-Na3-O10	95.23(4)	O9-Na3-O10	71.33(4)
O9-Na3-C10	95.66(5)	O4-Na3-C10	92.97(5)
O10-Na3-C10	24.35(4)	O2-C7-P1	105.69(10)
C5-O2-Na2	117.86(10)	C7-O2-Na2	121.11(9)
C8-O3-P1	121.59(11)	P1-O4-Na3	121.57(7)
P1-O5-Na1	129.14(7)	P1-O5-Na2	109.91(6)

Na1-O5-Na2	89.64(4)	Na2-O7-Na1	90.63(5)
C10-O10-Na1	148.16(10)	Na1-O9-Na3	98.14(5)
C10-O10-Na3	83.43(9)	Na1-O10-Na3	84.32(4)
O4-P1-O5	119.59(7)	O4-P1-O3	103.85(7)
O5-P1-O3	112.34(7)	O4-P1-C7	111.71(7)
O5-P1-C7	105.96(7)	O3-P1-C7	102.13(7)

[a] Symmetry transformations used to generate equivalent atoms: #1 x, y-1, z; #2 x, y+1, z; #3 -x, -y, -z; #4 x-1/2, -y+1/2, z-1/2; #5 x+1/2, -y+1/2, z+1/2

References for X-ray crystallography:

1. *Enraf-Nonius CAD-4/PC-Software. Version 1.5c* **1994**, Enraf-Nonius, Delft, The Netherlands.

2. North, A. C. T.; Phillips, D. C.; Mathews, F. S. A Semi-Empirical Method of Absorption Correction. *Acta Crystallogr. A* **1968,** *24* (3), 351–359.

3. Harms, K.; Wocadlo, S. *XCAD-4 - CAD4 Data Reduction* **1995**, University of Marburg, Marburg, Germany.

4. Sheldrick, G. M. A short history of SHELX. *Acta Crystallogr. A* **2008,** *64*, 112-122.

5. Barnes, C. L. ORTEP-3 for Windows - a version of ORTEP-III with a Graphical User Interface (GUI). *J. Appl. Crystallogr.* **1997,** *30* (1), 568-568.

6. Farrugia, L. J. WinGX suite for small-molecule single-crystal crystallography. *J. Appl. Crystallogr.* **1999,** *32* (4), 837-838.

7. (a) Duisenberg, A. J. M. Indexing in single-crystal diffractometry with an obstinate list of reflections. *J. Appl. Crystallogr.* **1992,** *25*, 92-96; (b) Duisenberg, A. J. M.; Hooft, R. W. W.; Schreursa, A. M. M.; Kroon, J. Accurate cells from area-detector images. *J. Appl. Crystallogr.* **2000,** *33* (2), 893-898.

8. Hooft, R.; Nonius, B. V. *COLLECT: a program for crystal data collection and processing user interface* **1998**.

9. Otwinowski, Z.; Minor, W. Processing of X-ray diffraction data collection and processing user interface. In *Macromolecular Crystallography, PtA*, 1997; Vol. 276, pp 307-326.

10. Sheldrick, G. M. *SADABS*, SADABS. Version 2007/2. Brucker AXS Inc.; Madison, Wisconsin, USA, 2007.

11. Beurskens, P. T.; Beurskens, G.; Bosman, W. P.; de Gelder, R.; Garcia-Granda, S.; Gould, R. O.; Israel, R.; Smits, J. M. M. *DIRDIF99: a program system* **1999**, Crystallography Laboratory, University of Nijmegen, The Netherlands.

12. Spek, A. L. *PLATON: a multipurpose crystallographic tool* **1998**, Utrecht University, Utrecht, The Netherlands.

13. *APEX2, SAINT and SADADS* **1998**, Bruker AXS Inc., Madison, Wisconsin, USA

Variation of a) intrinsic fluorescence of 1 with zinc and b) UV-Vis absorbance of 1 with zinc.

a)

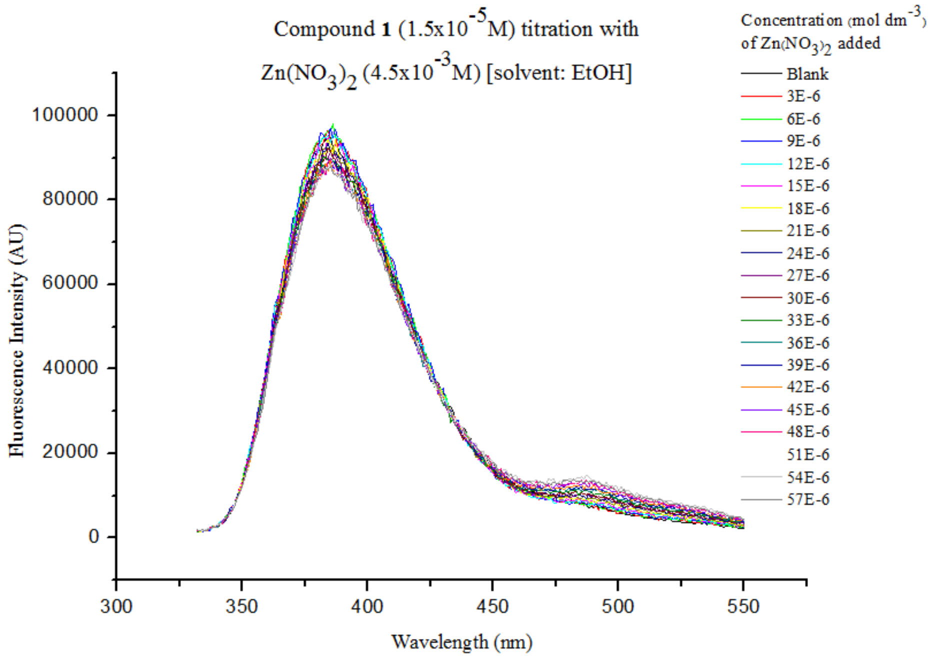

b)

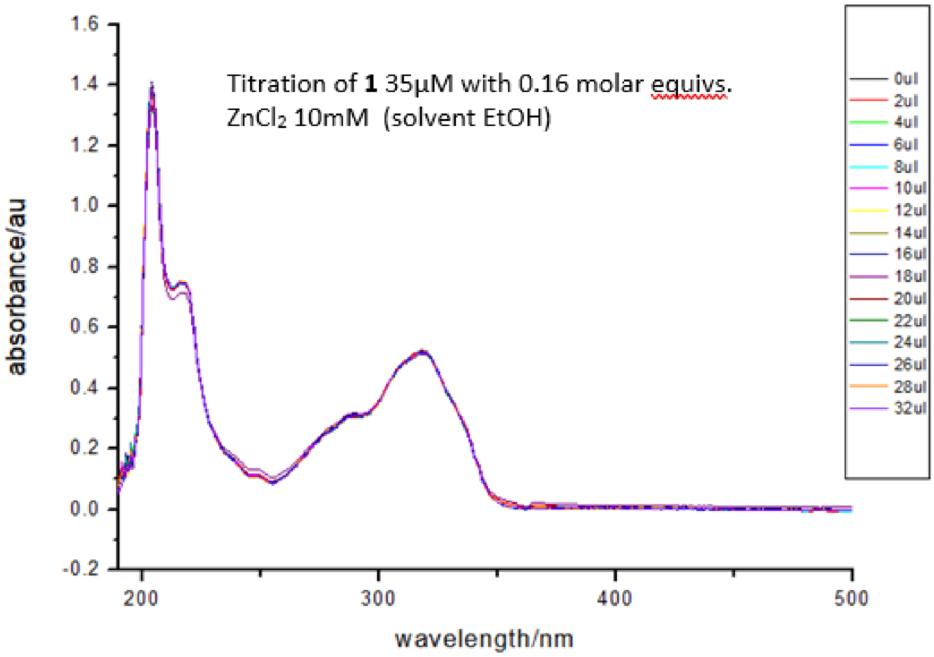

^{31}P NMR titration of compound 1 and 2 with $ZnCl_2$

(a) Spectra: top shows change in ^{31}P resonance of **1** in $CDCl_3$ (14mM) with added $ZnCl_2$ up to 0.25 equivs shown (no further change up to 1 equiv). Benesi-Hildebrand analysis shown below.
Bottom spectrum shows effect of Zn^{2+} (at 0.25 equiv) on ^{1}H NMR resonances of **1**. The oxymethyl proton resonance (Aryl-OC**H₂**P(O)(OEt)₂) shifts downfield in-keeping with Zn to phosphonyl oxygen interaction Zn—O=P(CH_2OAryl)$(OEt)_2$.

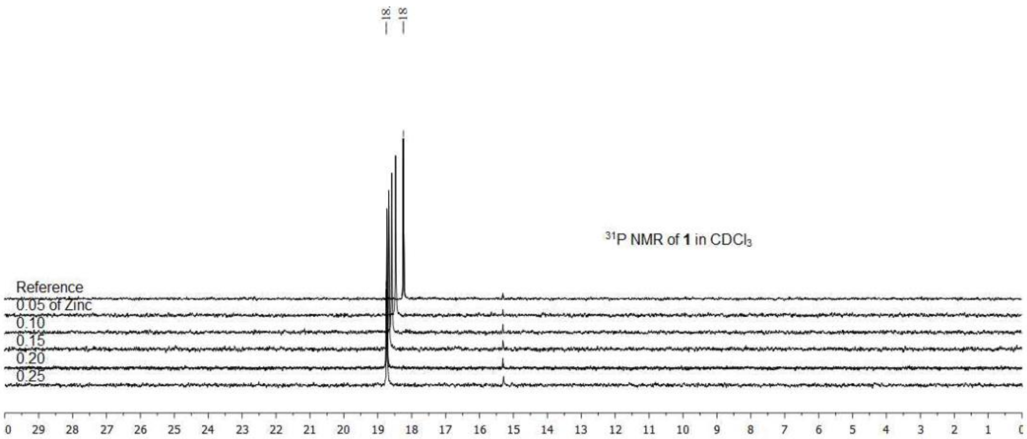

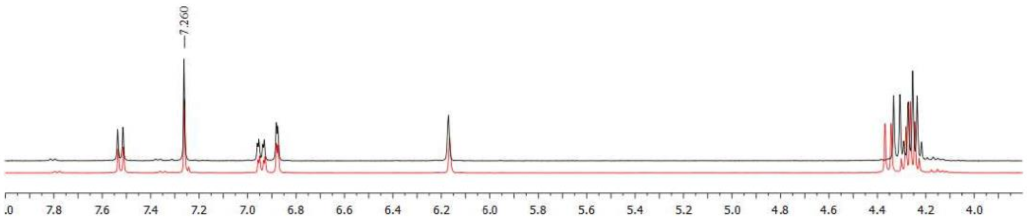

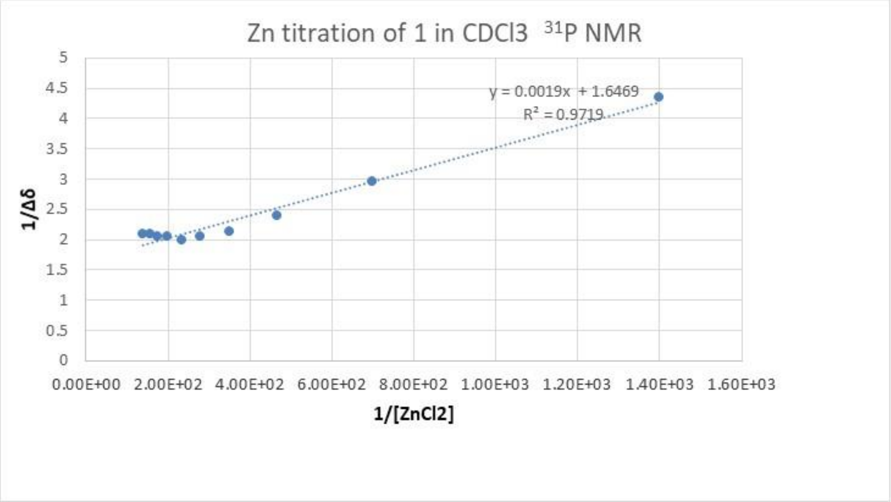

Ka 866±52 M^{-1}

(b) Spectra show change in ^{31}P resonance of **2** in $CDCl_3$ (14mM) with added $ZnCl_2$ up to 1.0 equiv . Benesi-Hildebrand analysis shown below.

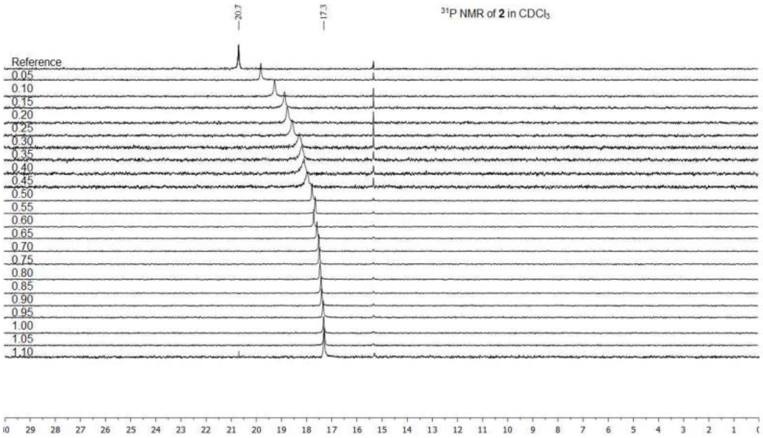

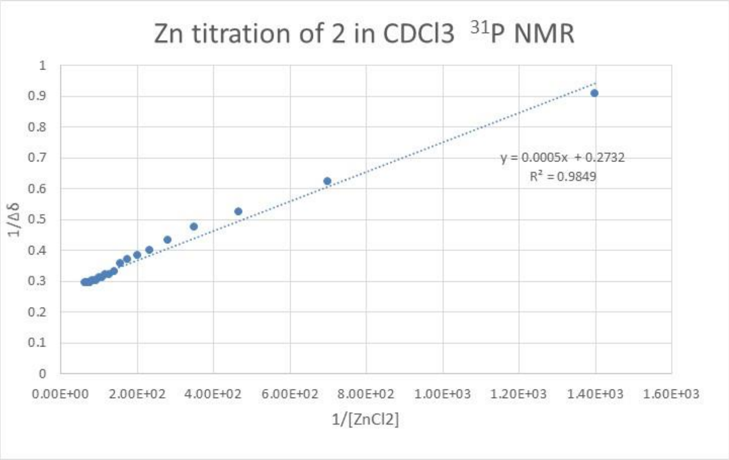

Ka 546±33 M^{-1}

AAS Calibration curves

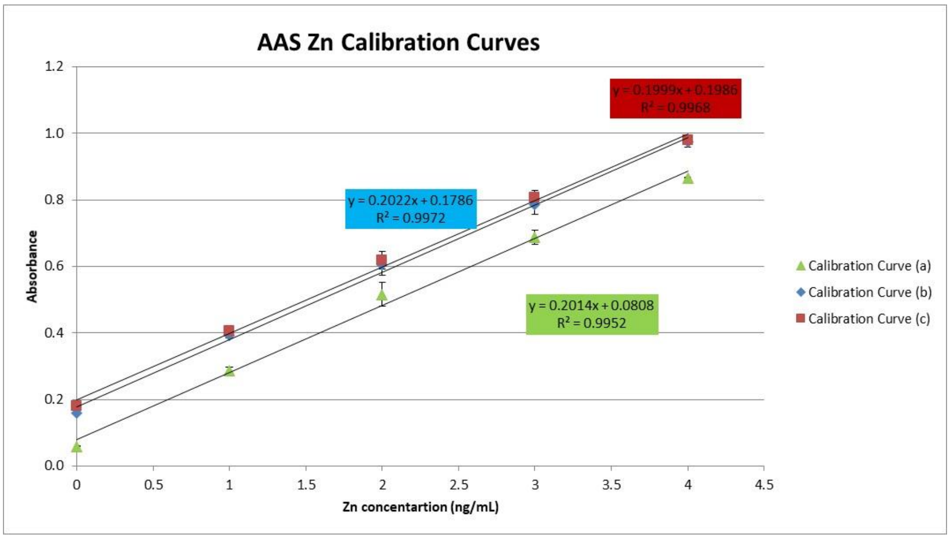

Calibration curve at low Zn concentrations (a) Zn in water (b) Zn in octanol saturated water (c) Zn in octanol saturated water spiked with DMSO solution of compound **1.** Curve (a) was reproduced with three different dilution regimes and was used to determine Zn in partition experiments.
The instrument absorbance output for Zn curve (a) is shown below.

ZnCAL ZERO 0.000 ng/mL

0.0601	0.059	0.0576	0.0606	0.0603	0.0596

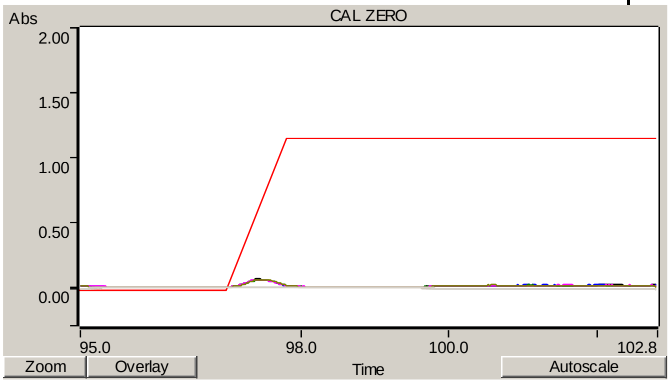

ZnSTANDARD 1 1.000 ng/mL

0.2909	0.2964	0.2764	0.2733	0.2749	0.2879

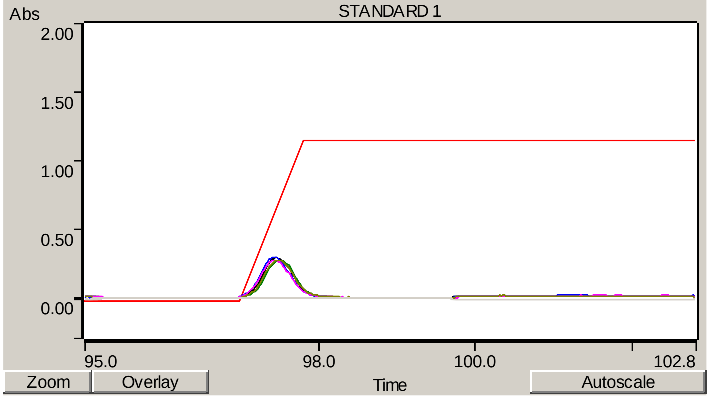

ZnSTANDARD 2 2.000 ng/mL

0.5509 0.4811 0.519 0.4845 0.4751 0.5160

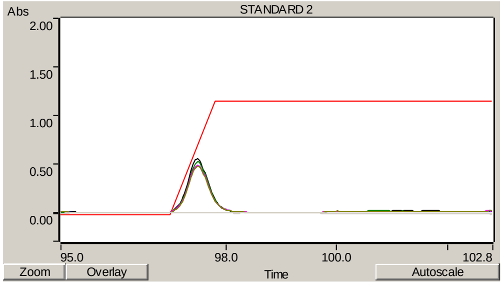

ZnSTANDARD 3 3.000 ng/mL

0.7088 0.6667 0.655 0.6488 0.6539 0.6878

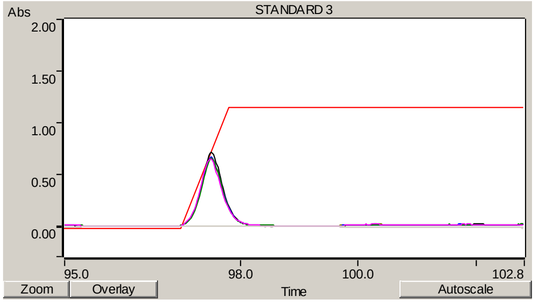

ZnSTANDARD 4 4.000 ng/mL

0.8641 0.8688 0.8358 0.8172 0.8525 0.8665

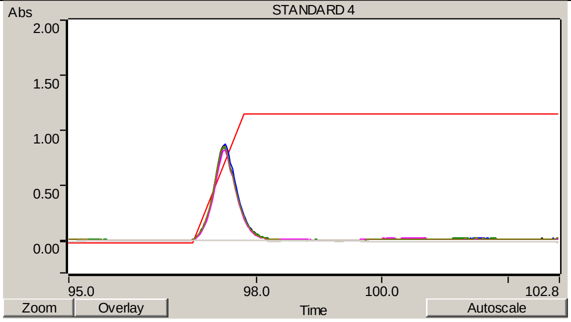

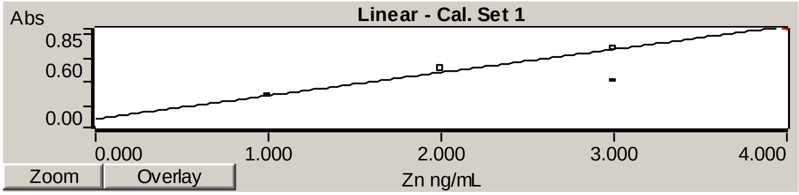

Curve Fit = Linear

Characteristic Conc = -0.386 ng/mL

r = 0.9982

Calculated Conc = -0.101 1.033 2.155 2.994 3.919

Residuals = 0.101 -0.033 -0.155 0.006 0.081

Copper Calibration Curve and related instrument output

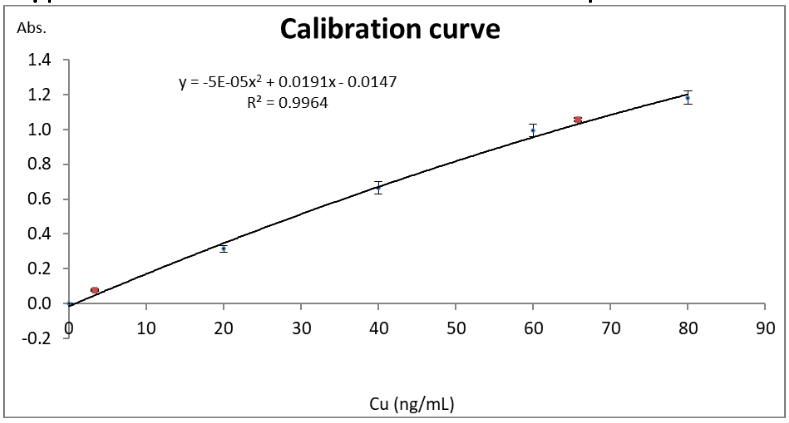

AAS Copper calibration curve (red points inserted are from Control solutions)

Instrument absorbance output for copper calibration is shown below.

```
Sample ID            Conc  ug/L     Readings     Mean Abs    StD
----------------------------------------------------------------------------
-------------------

CAL ZERO   0.00 ug/L  0.0021 0.0027 0.0021 0.0023 0.0003
```

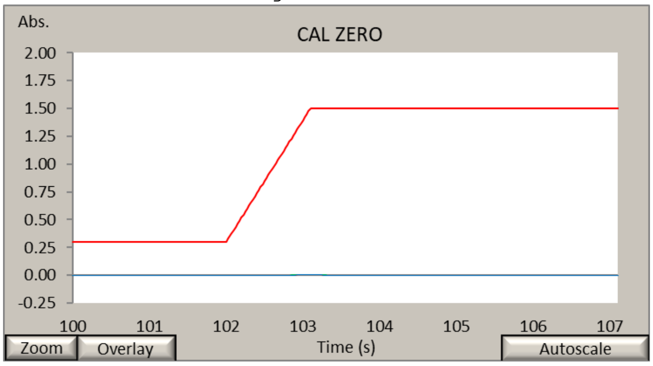


```
STANDARD 1 20.00 ug/L 0.3422 0.3049 0.2992 0.3154 0.0191
```

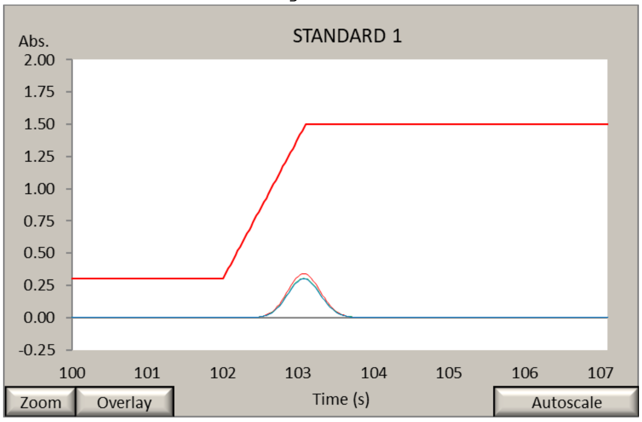

STANDARD 2 40.00 ug/L 0.7148 0.6388 0.6374 0.6637 0.0362

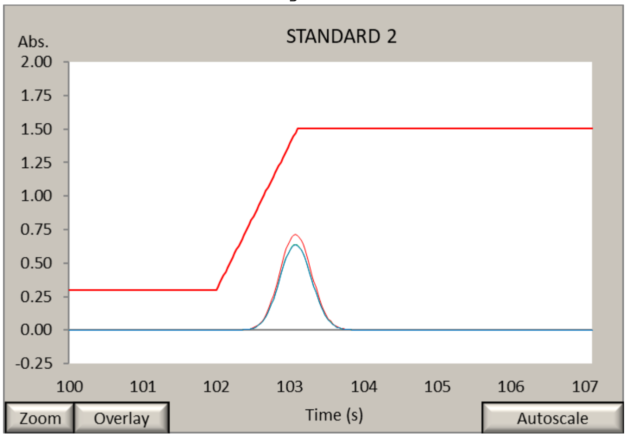

STANDARD 3 60.00 ug/L 1.0467 0.9757 0.9636 0.9953 0.0367

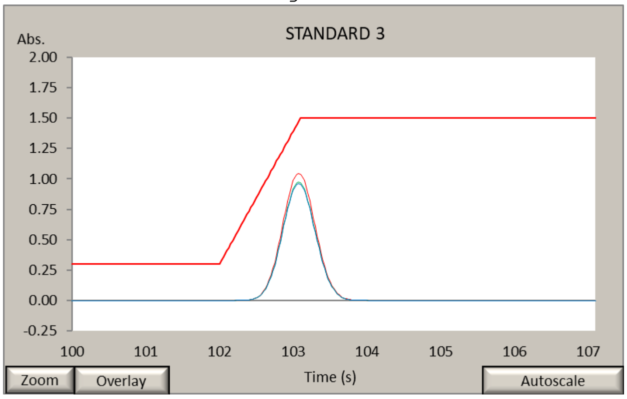

STANDARD 4 80.00 ug/L 1.2340 1.1610 1.1496 1.1815 0.0374

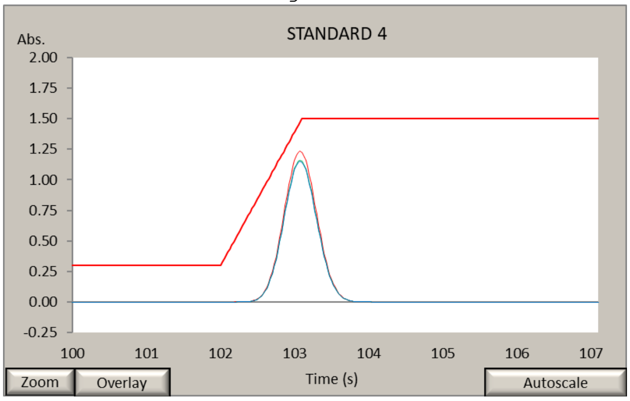

Curve Fit = Quadratic Origin
Characteristic Conc = 0.24 ug/L
r = 0.9981
Calculated Conc = 0.12 17.86 39.65
63.25 78.24
Residuals = -0.12 2.14 0.35
-3.25 1.76

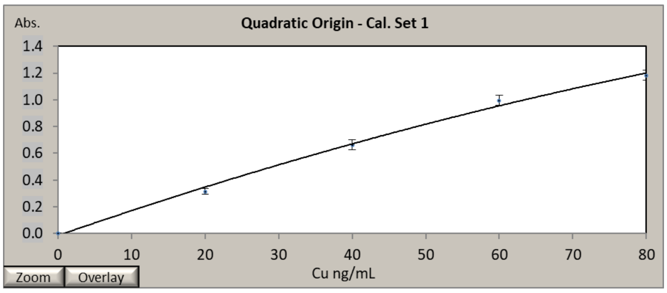

Blank 0.20 ug/L 0.0185 0.0179 0.0189 0.0184 0.0004

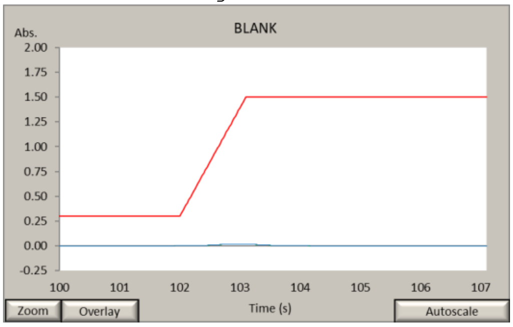

Control1 Cu 65.79 ug/L 1.0459 1.0521 1.0665 1.0548 0.0086

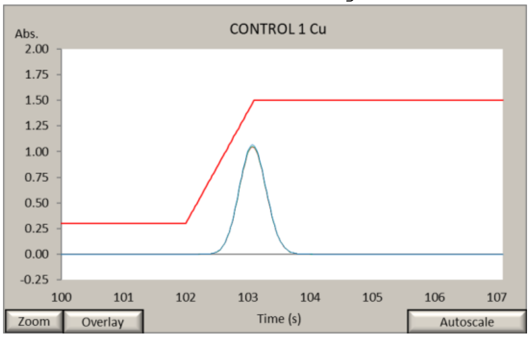

Control2 Cu 3.56 ug/L 0.0821 0.0756 0.0765 0.0780 0.0028

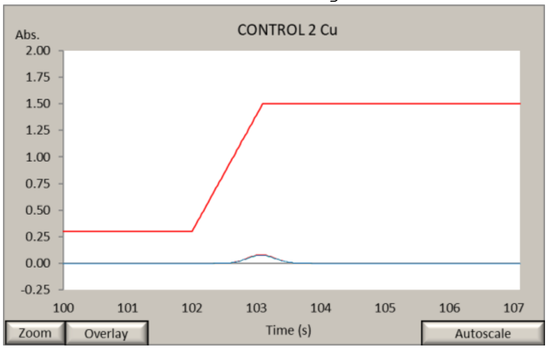

Procedure for preparation of ADDLs and fibrillated Aβ(42)

Aβ(1-42) was purchased from California Peptide Research, Inc. (California, USA). The amyloid derived diffusible ligands (ADDLs) were prepared as previously described. Briefly, 1 mg of peptide was dissolved in ice-cold hexafluoro-2-propanol (HFIP) (Sigma, Dorset, UK) to a concentration of 1 mM and kept at room temperature for 1 hour with the lid of the vial closed. The solution was then placed back on ice for 10 min. Following this, the solution was divided into 4 equal aliquots in microcentrifuge tubes (0.25 mg of peptide per aliquot). The HFIP was allowed to evaporate overnight in a fume hood at room temperature. The tubes were then transferred to a speedvac and dried down for 10 min to remove any residual HFIP and produce a thin clear film. A 5 mM Aβ stock solution was made by adding fresh anhydrous dimethyl sulfoxide (DMSO) (Sigma, Dorset, UK) to 0.25 mg peptide. The stock solution was then diluted to 100 μM using neurobasal medium without phenol red (Invitrogen, Paisley, UK). To prepare the ADDLs, the 100 μM peptide solution was stored at 4 ^{o}C for 24 h. Following this, the solution was centrifuged at 14,000 g for 10 min and the supernatant transferred to a new tube. To prepare fibrillated Aβ, the 100 μM peptide solution was incubated at 37 ^{o}C with constant agitation for 72 h.
